# Versatile approach for functional analysis of human proteins and efficient stable cell line generation using FLP-mediated recombination system

**DOI:** 10.1371/journal.pone.0194887

**Published:** 2018-03-28

**Authors:** Roman J. Szczesny, Katarzyna Kowalska, Kamila Klosowska-Kosicka, Aleksander Chlebowski, Ewelina P. Owczarek, Zbigniew Warkocki, Tomasz M. Kulinski, Dorota Adamska, Kamila Affek, Agata Jedroszkowiak, Anna V. Kotrys, Rafal Tomecki, Pawel S. Krawczyk, Lukasz S. Borowski, Andrzej Dziembowski

**Affiliations:** 1 Laboratory of RNA Biology and Functional Genomics, Institute of Biochemistry and Biophysics, Polish Academy of Sciences, Warsaw, Poland; 2 Institute of Genetics and Biotechnology, Faculty of Biology, University of Warsaw, Warsaw, Poland; Friedrich-Loeffler-Institute, GERMANY

## Abstract

Deciphering a function of a given protein requires investigating various biological aspects. Usually, the protein of interest is expressed with a fusion tag that aids or allows subsequent analyses. Additionally, downregulation or inactivation of the studied gene enables functional studies. Development of the CRISPR/Cas9 methodology opened many possibilities but in many cases it is restricted to non-essential genes. Recombinase-dependent gene integration methods, like the Flp-In system, are very good alternatives. The system is widely used in different research areas, which calls for the existence of compatible vectors and efficient protocols that ensure straightforward DNA cloning and generation of stable cell lines. We have created and validated a robust series of 52 vectors for streamlined generation of stable mammalian cell lines using the FLP recombinase-based methodology. Using the sequence-independent DNA cloning method all constructs for a given coding-sequence can be made with just three universal PCR primers. Our collection allows tetracycline-inducible expression of proteins with various tags suitable for protein localization, FRET, bimolecular fluorescence complementation (BiFC), protein dynamics studies (FRAP), co-immunoprecipitation, the RNA tethering assay and cell sorting. Some of the vectors contain a bidirectional promoter for concomitant expression of miRNA and mRNA, so that a gene can be silenced and its product replaced by a mutated miRNA-insensitive version. Our toolkit and protocols have allowed us to create more than 500 constructs with ease. We demonstrate the efficacy of our vectors by creating stable cell lines with various tagged proteins (numatrin, fibrillarin, coilin, centrin, THOC5, PCNA). We have analysed transgene expression over time to provide a guideline for future experiments and compared the effectiveness of commonly used inducers for tetracycline-responsive promoters. As proof of concept we examined the role of the exoribonuclease XRN2 in transcription termination by RNAseq.

## Introduction

Deciphering a protein’s function requires investigating its subcellular localisation, identifying its binding partners, and performing multiple functional assays. There are many ways to achieve these goals, with different amounts of required time and effort as well as variable biological relevance of the results obtained. The usual course of action is to express the protein of interest with a fusion tag, a short peptide or a domain, that aids or allows biochemical, cellular or functional analysis. Study of one protein often leads to follow-up experiments that involve other proteins, which can quickly multiply the amount of work required to comprehensively answer the original question. Consequently, straightforward methods or tools that can provide answers to a number of questions are called for.

Ectopic expression is widely used for investigations of human proteins. It can be achieved by transient or stable transfection of cultured cells with a plasmid or virus. Alternatively, one can perform targeted genomic manipulation to engineer the gene of interest in its natural locus. This used to be difficult and time-consuming for most vertebrate cell lines before the advent of CRISPR-based approaches [1, 2, 3]. Genome editing has the crucial advantage in that the studied gene is expressed at its natural levels and naturally responds to all stimuli. However, this approach can prove to be problematic if control of gene expression is required or specific alleles are to be tested. On the other hand, transfection, transient or stable, offers a lot of flexibility in transgene sequence, allowing for the use of stronger, weaker, or even regulated promoters.

Of the two transfection modes–transient and stable–the first one is obviously easier and faster but suffers from low reproducibility and heterogeneity of cell populations. Also, cells are analysed shortly after transfection, and can still be suffering from stress induced by transfection procedure. Generating stable cell lines eliminates such caveats but requires a considerable amount of time, especially if creating the DNA constructs and cell selection following transfection run into unforeseen problems. These two steps can be streamlined with some careful planning and creating an overarching strategy.

The first step is choosing a reliable cloning method. The traditional one, involving digestion with restriction enzymes and ligation with DNA ligase strongly depends on the target’s sequence; the efficiency of the procedure is highly variable and establishing a universal protocol is quite difficult. Sequence-independent or recombination based cloning methods like In-Fusion, Gibson assembly, PIPE (polymerase incomplete primer extension) or SLIC (sequence and ligation independent cloning) overcome these difficulties [4, 5, 6, 7, 8, 9].

The second step is to use parental cell lines that have been pre-engineered to improve genomic integration of the transgene [10, 11, 12]. One commonly used solution is site-specific recombination employing the FLP recombinase (S1 Fig) [13, 14, 15]. In this approach, which is commercially available from Thermo Fisher Scientific as the Flp-In System, the genome of the parental cells contains an FRT sequence (FLP recombination target), which is recognized by the yeast FLP recombinase. The gene of interest is cloned into a plasmid that contains a non-functional antibiotic resistance gene, devoid of a promoter and the initiation codon. Upon FLP-mediated recombination, the plasmid DNA is inserted into the chromosome in such a way that the coding sequence of the new selection marker is substituted for that of the old one. As a result the cells lose one antibiotic resistance and gain another (S1 Fig).

When generating transgenic cell lines, a common concern is the cells’ isogenicity, *i*.*e*. having the transgene integrated in the same locus. With random integration of a plasmid an isogenic cell line is obtained by selecting a single cell and growing it for an appropriate time. This leads to a monoclonal cell line. Usually several clones must be expanded and tested to ensure that the integration event results in expression of the desired protein and that no relevant side effects of the integration have arisen. Since parental Flp-In cells are isogenic to begin with, the FRT site occupying the same locus in all cells, clonal selection can be omitted, which greatly simplifies the procedure. Furthermore, monoclonal lines can suffer from occasional genome rearrangements that occur in cells cultured *in vitro*, which could lead to substantial differences between lines [16, 17]; in polyclonal cell lines the effect is diluted across the population. In all, establishing of stable cell lines with the help of the Flp-In approach is much more straightforward than the traditional approach, where a plasmid is randomly integrated into the genome.

Several cell lines compatible with the Flp-In system are available [15, 18, 19, 20], including commercial ones. Among them are HeLa and 293 [21, 22, 23, 24], which are the most frequently used non-primary cell lines in basic science and biotechnology [25]. Plenty of detailed transcriptomic and genomic data have been acquired with HeLa and 293 cells, providing rich context for interpretation of new results [25, 26, 27, 28, 29, 30, 31]. However, it should be noted that the Flp-In system is not limited to currently available Flp-In competent cells, an FRT sequence can be introduced into genomes other cell lines.

Importantly, expression of the transgene can be driven by inducible promoters, allowing a degree of control over the level, time of expression and its onset. Several inducible systems are used in mammalian cells [32, 33], including ones which respond to ponasteron A (ecdysone analogue) [34, 35, 36], IPTG [37, 38, 39] or tetracycline [40]. The system that we implement, commercially available from Invitrogen (T-REx), utilizes parts of the bacterial tetracycline resistance operon: the repressor protein (TetR) and the operator element found in the promoter of the operon’s structural genes [41]. The operator sequence is inserted into the transgene promoter, where it recruits the repressor protein. This results in constitutive repression of transcription, which is alleviated by tetracycline [42, 43, 44]. Other tetracycline-dependent systems employ fusions of TetR with a transcriptional activator or repressor and allow for positive (TetON) as well as negative (TetOFF) regulation of transgene expression [45].

We have established a straightforward method to generate Flp-In-based cellular models for functional studies of human proteins. We have created and validated a robust series of vectors designed for an efficient cloning strategy that enables cheap and easy generation of DNA constructs, and we have combined the efficient cloning with the Flp-In T-REx system for stable generation of inducible cell lines. The cloning procedure has been successfully applied to more than 500 DNA constructs, most of which were obtained in the first attempt. Our vectors facilitate localization studies, protein purification, *in vitro* and *in vivo* protein interaction studies (co-IP, BiFC, FRET), protein dynamics studies (FRAP, photoactivation, etc.) and studies that involve RNA tethering. We have analysed transgene expression over time in order to provide a guideline for future experiments. We also compare the utility of two commonly used inducers for tetracycline-responsive promoters, namely tetracycline and doxycycline.

A subset of our vectors enables inducible downregulation of the endogenous gene of interest and concomitant expression of its protein product from an ectopic allele. This approach can be useful *e*.*g*. for functional validation of newly identified mutations. We used these vectors to establish a cellular model for investigation of the 5’-3’ exoribonuclease XRN2. Deep RNA sequencing analysis of cells devoid of the XRN2 ribonucleolytic activity revealed that this activity is required for transcriptional termination. Our detailed protocols will allow smooth transfer of this strategy to other laboratories.

## Materials and methods

### Vector construction

pKK and pKK-RNAtag vectors were constructed by modifying pcDNA5/FRT/TO (Thermo Fisher Scientific); pKK-BI16, pKK-RNAi, pKK-BiFC and pKK-FRET vectors were constructed by modifying BI16 (a kind gift from Ed Grabczyk), which is in turn derived from pcDNA5/FRT/TO [46]. Standard cloning and SLIC methods were used. Coding sequences of fluorescent proteins and BirA^R118G^ were PCR-amplified from plasmids acquired from Addgene (ID 22010, 22011, 27793, 27795, 27798, 36047, 56172, 62383, 74252, 74279), deposited by: Kyle Roux, Jonathan Weissman, Steven Vogel, Michael Lin, Michael Davidson, Chang-Deng Hu. The MBP tag sequence came from MS2MBP plasmid [47]. The 3xMS2 stem-loop sequence came from pAdML-M3 [48] and the 24xMS2 stem-loop sequence came from pCR24MS2SL–a kind gift of Witold Filipowicz. The N-terminator peptide sequence came from plasmids described previously [49]. All vector sequences are presented in S1 Supporting Information (annotated GenBank format) as well as will be available on our lab’s website, http://adz.ibb.waw.pl (SnapGene format). Vectors will be made available from Addgene.

### Sequence and ligation independent cloning (SLIC)

A simplified SLIC protocol was applied [7]. Detailed description of the SLIC protocol and instructions for using the pKK-RNAi backbone can be found in S2 and S3 Supporting Information, respectively. Sequences of synthetic DNA (primers and miRNA cassettes) used for construction of plasmids applied in validation experiments are shown in S4 Supporting Information.

### Cell culture

HeLa Flp-In T-REx (a kind gift from Matthias Hentze) [19] and 293 Flp-In T-REx cells (R78007, Thermo Fisher Scientific) were cultured in Dulbecco’s Modified Eagle’s Medium (Gibco) supplemented with 10% fetal bovine serum (FBS; standard FBS hereafter) (Gibco) at 37°C in a humidified 5% CO_2_ atmosphere. Where indicated, certified tetracycline-free FBS (Clontech and Biochrom GmbH) was used instead of standard FBS. The identity of cells was confirmed by DSMZ (Germany).

### Gene expression inducers

Tetracyline (550205, Thermo Fisher Scientific) or doxycycline (D9891, Sigma) was added to 96% ethanol at a concentration of 1–5 mg/ml, rotated for 30 minutes at room temperature and incubated overnight at -20°C. On the following day the solutions were rotated again for 30 minutes at room temperature, filtered (0.22 μm) and diluted with ethanol to a final concentration of 0.1 mg/ml. This stock solution (0.1 mg/ml) was stored at -20°C.

### Stable cell line generation

Parental cells were plated onto 6-well plates and cultured for 24 hours. On the next day cells were co-transfected using 2 μl of TransIT-2020 reagent (Mirus) with 0.3 μg of gene-of-interest construct and 1.0 μg of pOG44 (Thermo Fisher Scientific). Twenty four hours after transfection, cells were replated to 60 mm dishes and subjected to selection with hygromycin B (50 and 175 μg/ml for 293 and HeLa cells, respectively) (Thermo Fisher Scientific) and blasticidin (10 μg/ml) (Invivogen) for up to a month. A detailed day-by-day protocol is described in S5 Supporting Information. Where applicable, colonies were stained with crystal violet (0.5% w/v).

### Western blot

Total protein cell extracts were prepared as described previously [50]. Protein concentration was determined by the Bradford method. The protein extracts, 20 μg per well, were separated by sodium dodecyl sulfate polyacrylamide gel electrophoresis (SDS–PAGE) and transferred to a nitrocellulose membrane (Protran, Whatman GmbH). Western blotting was performed according to standard protocols using the following primary antibodies: anti-EGFP (dilution 1:1000, sc-9666, Santa Cruz Biotechnology), anti-THOC5 (dilution 1:1500, ab86070, Abcam), anti-XRN2 (dilution 1:1000, sc-365258, Santa Cruz Biotechnology). Appropriate horseradish peroxidase-conjugated secondary antibodies (dilution 1:10000, 401393, 401215, Calbiochem) were detected by enhanced chemiluminescence (170–5061, BioRad) according to the manufacturer’s instructions.

### Measurement of luciferase activity

293 Flp-In T-REx cells were stably transfected with the BI16 vector. Cells were plated on a 96-wells plate at 5,000 cell per well. Luciferase activity was measured with Dual-Glo Luciferase Assay System (E2920, Promega) according to the manufacturer’s instructions. DTX880 plate reader (Beckman Coulter) was used for measurement of luminescence. Luciferase activity was normalized to the number of cells, which was assessed using AlamarBlue (see next section).

### Cell viability assay

The assay was performed using the AlamarBlue reagent (DAL1100, Thermo Fisher Scientific) according to the manufacturer’s instructions. Briefly, AlamarBlue (1/10 of culture volume) was added to cell culture which was subsequently continued to grow for one hour. Fluorescence was measured with a DTX880 plate reader (Beckman Coulter) using 535/25 and 595/35 filters (excitation and emission, respectively).

### Flow cytometry

#### Comparison of gene expression inducers

293 stable cell lines were plated onto 6-well plates at 250,000 cells per well. 24 hours later the cells were treated with indicated concentrations of tetracycline or doxycycline. 24 hours after the cells were detached by trypsin treatment, washed with PBS and subjected to western blot analysis. 293 EGFP-THOC5 were also subjected to flow cytometry analysis using a BD FACSCalibur flow cytometer (BD Biosciences). A gate was applied to the FSC/SSC plot to exclude dead cells and debris. 10,000 events were collected. Data were analysed using the Flowing Software *(*www.flowingsoftware.com). Mean fluorescent intensity of EGFP-positive cells was calculated.

#### Expression kinetics

HeLa stable cell lines were plated onto 24-well plates at 50,000 cells per well. 24 hours later the cells were induced with tetracycline at a final concentration of 50 ng/ml in 6 hour intervals. 24 hours after the first induction the cells were detached by trypsin treatment, diluted with PBS, and analysed an Attune NxT flow cytometer equipped with 488 nm and 561 nm laser diodes (Thermo Fisher Scientific). A gate was applied to the FSC/SSC plot to exclude dead cells and debris. Doublet discrimination was performed based on the FSC-A/FSC-H plot. 20,000 events were collected. Data were analysed using Attune NxT software. Mean fluorescent intensity of EGFP-positive cells was calculated.

#### Validation of XRN2 stable cell lines

Cells were plated onto 6-well plates at 500,000 cells per well and induced upon plating with tetracycline (25 ng/ml). 24 hours later the cells were detached by trypsin treatment, washed with PBS, and analysed with an Attune NxT flow cytometer equipped with 488 nm and 561 nm laser diodes (Thermo Fisher Scientific). A gate was applied to the FSC/SSC plot to exclude dead cells and debris. Doublet discrimination was performed based on the FSC-A/FSC-H plot. 20,000 events were collected. Data were analysed using Attune NxT software.

### Fluorescence microscopy

Stable cell lines expressing XRN2 were analysed by the following protocol: 24 hours prior to imaging cells were seeded on poly-L-lysine-coated (see below) 8-well Lab-Tek II Chambered Coverglass culture vessels (155409, Thermo Fisher Scientific) at 30,000 per well and induced with tetracycline (25 ng/ml). Before imaging, Hoechst 33342 dye was added to the medium (2 ng/ml) for 30 minutes to stain cell nuclei. After staining medium was replaced. Images were collected using a FluoView1000 Olympus confocal system with a PLANAPO 60x/1.40 oil immersion lens. Live cell imaging was performed in a temperature (37°C) and CO_2_ (5%) incubator.

All other stable cell lines were analyzed as follows: cells were plated onto 8-well Lab-Tek II Chambered Coverglass culture vessels coated with poly-L-lysine at 7,000 (HeLa) or 10,000 (293) cells per well. Tetracycline at a final concentration of 25 ng/ml was added to the medium upon plating. On the following day the cells were stained with Hoechst 33342 for 30 minutes (final concentration of 50 ng/ml). Live imaging was done with an FV10i system (Olympus), maintaining the cells at 37°C in a humidified 5% CO_2_ atmosphere delivered from a The Brick gas mixer (Life Imaging Services). Fluorescence was excited with 405 and 483 laser diodes and collected with a SuperApochromat 60x/1.2 water immersion lens. Where applicable, Z stacks were collected with 350 nm spacing.

### Preparation of poly-L-lysine-coated coverglasses

Poly-L-lysine hydrobromide (P1274, Sigma) was dissolved in sterile water to 0.01% (w/v). The solution was sterilized with a 0.22 micron filter before freezing aliquots at -20°C. Before use, the solution was thawed and 200 μl was added to each well of 8-well Lab-Tek II Chambered Coverglass to fully coat the surface of each well. Coating was performed for 1 hour at 37°C, after that the solution was removed and coverglasses were dried at room temperature for 20 minutes under a laminar flow hood. The poly-L-lysine solution was collected and stored at -20°C for repeated use. Dried coverglasses were stored at room temperature or directly used for cell seeding.

### RNA isolation, library construction and deep-sequencing

Total RNA was isolated with the TRI Reagent (T9424, Sigma) according to the manufacturer’s instructions. DNA contamination from 2 μg of nucleic acids was removed by 2 U of TURBO DNase (AM2238, Ambion) in 20 μl of the supplied buffer in 37°C for 30 min. RNA was extracted with phenol-chloroform, precipitated with ethanol and resuspended in RNase free water. Concentration was measured with NanoDrop 2000 Spectrophotometer (Thermo Fisher Scientific). Prior to library preparation, to provide an internal performance control for further steps, 1 μg of RNA was mixed with 4 μl of 1:99 diluted ERCC RNA Spike-In Control Mix 1 (4456740, Ambion). Subsequently, rRNA was depleted using Ribo-Zero Gold rRNA Removal Kit (MRZG12324, Human/Mouse/Rat, Illumina) according to the manufacturer’s protocol.

RNA-seq libraries were constructed as previously described in Sultan et al., 2012 [51] with minor modifications. Fragmentation and first strand cDNA synthesis were performed as in TruSeq RNA Library Prep kit v2 protocol (Illumina, RS-122-2001, instruction number 15026495 Rev. D), using SuperScript III reverse transcriptase (18080–085, Thermo Fisher Scientific). For second strand synthesis, reaction mixtures were supplemented with 1 μl of 5x First Strand Synthesis Buffer (18080–085, Thermo Fisher Scientific), 15 μl 5x Second Strand Synthesis Buffer (10812–014, Thermo Fisher Scientific), 0.45 μl 50 mM MgCl, 1 μl 100 mM DTT, 2 μl of 10 mM dUNTP Mix (dATP, dGTP, dCTP, dUTP, 10 mM each, R0182, R0133, Thermo Fisher Scientific), water to 57 μl, 5 U *E*. *coli* DNA Ligase (M0205L, NEB), 20 U *E*. *coli* DNA Polymerase I (NEB, M0209L), 1 U RNase H (18021–071, Thermo Fisher Scientific), and incubated at 16°C for 2h. Further steps: purification, end-repair, A-tailing and adapter ligation were performed as described in the previously mentioned TruSeq kit protocol with one modification: the first purification eluate was not decanted from the magnetic beads and subsequent steps were performed with the beads in solution. Instead of a new portion of magnetic beads, an equal volume of 20% PEG 8000 in 2.5 M NaCl was added and the DNA bound to the beads already present in the mixture. After the second clean up procedure after adapter ligation, the supernatant was separated from the beads and treated with USER Enzyme (M5505L, NEB) in 1x UDG Reaction Buffer (M0280S, NEB) at 37°C for 30 min. The digestion step ensures that the second strand synthetized with dUTP instead of dTTP is removed from cDNA, resulting in strand-specific libraries. The product was amplified using 1 U of Phusion High-Fidelity DNA Polymerase (F530L, Thermo Fisher Scientific) in 1x HF Buffer supplemented with 0.2 mM dNTP Mix, and the following primers: PP1 (5’-AATGATACGGCGACCACCGAGATCTACACTCTTTCCCTACACGA-3’), PP2 (5’-CAAGCAG AAGACGGCATACGAGAT-3’). TruSeq kit protocol temperature scheme with 12 amplification cycles and subsequent purification procedure was applied. Enriched library quality was verified using 2100 Bioanalyzer and High Sensitivity DNA kit (5067–4626, Agilent). The libraries’ concentration was estimated by qPCR means with KAPA Universal Library Quantification Kit (KK4824, Kapa Biosystems), according to the supplied protocol. Sequencing was carried out on an Illumina NextSeq 500 sequencing platform, using NextSeq 500 High Output Kit (150 cycles) (FC-404-1002, Illumina) and standard libraries denaturation and pair-end sequencing procedures (Instructions: 15048776 Rev. D, 15046563 Rev. F) of 2x75 cycles.

### Analysis of deep sequencing data

Strand-specific RNAseq libraries (dUTP RNA) were prepared in triplicate for each condition and sequenced in the 75-nt paired-end mode to the average depth of 10 million reads (GEO accession number: GSE99421, security token: ghmheeqytvkfbof, https://www.ncbi.nlm.nih.gov/geo/query/acc.cgi?acc=GSE99421). Reads were mapped to the reference human genome (GRCh38) using the STAR short read aligner [52]. Quality control, read processing and filtering, visualization of the results and counting of reads for the Genecode v22 comprehensive annotation were performed using custom scripts using elements of the RSeQC, BWtools, BEDtools and SAMtools packages. Transcripts were annotated using StringTie [53]. The merged unmodified 293 Flp-In T-REx cells annotation was used to perform meta-gene analysis of the transcriptional read-through in wild-type and mutant XRN2 cells. Cumulative, strand-specific signal was calculated across 250 nt windows placed directly downstream to 3' ends of highly expressed (TPM > 10), spliced transcripts and normalized to the signal within the last 250 nt of the analyzed transcripts.

## Results and discussion

### Construction of SLIC-competent vectors

**Fig 1:**
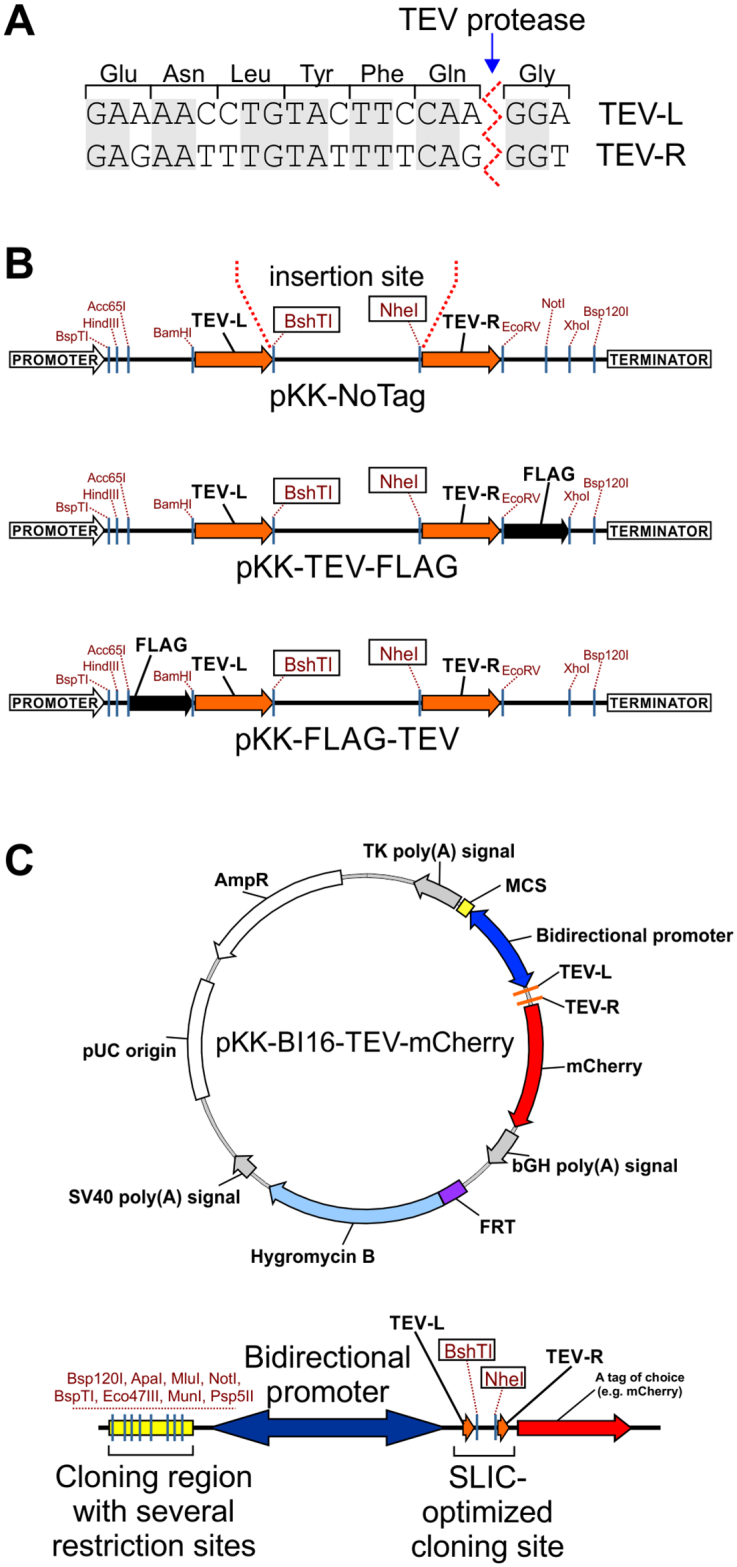
The pKK vector series. (A) Nucleotide sequences of the TEV-L and TEV-R. Translation to protein and TEV protease cleavage site are shown. Shaded letters indicate nucleotides common to both sequences. (B) Cloning sites of selected pKK vectors. Potentially useful unique restriction sites are marked. For all pKK vectors BshTI and NheI restriction enzymes are used for vector linearization before DNA cloning with the help of our universal SLIC protocol. All pKK vectors have promoters with the TetR repressor binding site. (C) Example of a pKK-BI16 vector. Map of pKK-BI16-TEV-mCherry vector and its cloning region (bottom diagram). Useful unique restriction sites are marked. The tetracycline operator sequences are present in all vectors of pKK-BI16 series, thus, transcription in both directions is regulated by the tetracycline repressor.

A new set of vectors was designed to fulfil four requirements: 1) compatibility with sequence-independent, straightforward and efficient cloning; 2) minimal number of primers required for cloning into different vectors; 3) compatibility with FLP-mediated stable cell line generation; 4) regulated transgene expression. To achieve these goals we modified the pcDNA5/FRT/TO vector (Thermo Fisher Scientific) that enables FLP-mediated cell line generation and contains a tetracycline-regulated CMV promoter to drive expression of the cloned CDS (S1 Fig). The aim of our modification was to make the vector suitable for the SLIC approach (sequence and ligation independent cloning) [4]. The multiple cloning site of the vector was modified by introducing two 21-nucleotide long sequences, the SLIC arms (Fig 1). These fragments are complementary to the ends of the linear DNA fragment to be cloned. They encompass TEV-L and TEV-R sequences which encode a 7 amino acid long peptide which is recognized by the tobacco etch virus (TEV) protease [54] (Fig 1A).

Depending on the vector, the SLIC arms lie upstream or downstream of a tag coding sequence (Fig 1B; maps of all reported vectors are presented in S6 Supporting Information). Several vectors with different tags were constructed: 1) short tags, *e*.*g*. FLAG; 2) fluorescent proteins, *e*.*g*. EGFP; 3) humanized biotin ligase–BirA, which can be used for identification of proximal and interacting proteins; 4) other proteins, *e*.*g*. protein A or maltose binding protein (MBP), for which several molecular tools are described (vectors pKK, Table 1 and S7 Supporting Information). The coding sequence of the protein of interest is inserted between the SLIC arms. For the N-terminal fusions the initiation codon originates from the vector and the termination codon is introduced within the cloned fragment, and vice-versa in the case of C-terminal fusions. The same forward primer can be used to create both N- and C-terminal fusions, however, a specific reverse primer is required (with or without the stop codon). Thus, only 3 primers are sufficient to prepare constructs encoding a protein of interest in N- or C-terminal fusion with different tags and each tag encoded by pKK-series vectors can be cleaved off with the TEV protease. Importantly, synonymous codons were used for designing of TEV-L and TEV-R sequences to mitigate the possibility of intramolecular homologous DNA recombination.

**Table 1:** List of created vectors.

Vector name	Tag position	Features encoded	References
**pKK Vector Series**			
pKK-NoTag	---	only SLIC arms (TEV-L and TEV-R)	---
pKK-FLAG-TEV	N-terminus	FLAG tag	[55]
pKK-HA-TEV	N-terminus	HA tag derived from a fragment of human influenza virus hemagglutinin	[56]
pKK-FLAG-BirA-TEV	N-terminus	humanized biotin ligase, FLAG-tagged	[57], [58]; Addgene ID: 36047
pKK-MBP-TEV	N-terminus	maltose binding protein	[47], [59], [60]
pKK-mTagBFP-TEV	N-terminus	blue fluorescent protein (λ_Exc_ = 402 nm, λ_Em_ = 457 nm)	[61], [62] Addgene ID: 62383
pKK-mCerulean-TEV	N-terminus	cyan fluorescent protein (λ_Exc_ = 433 nm, λ_Em_ = 475 nm)	[63], [64], [65] Addgene ID: 27795
pKK-mVenus-TEV	N-terminus	green fluorescent protein (λ_Exc_ = 515 nm, λ_Em_ = 528 nm)	[64], [65], [66] Addgene ID: 27793
pKK-mAmber-TEV	N-terminus	non-absorbing, non-emitting Venus mutant used as negative control for FRET measurements	[64], [65] Addgene ID: 27798
pKK-EGFP-TEV	N-terminus	green fluorescent protein (λ_Exc_ = 488 nm, λ_Em_ = 507 nm)	[67], [68]
pKK-mEGFP-TEV	N-terminus	green fluorescent protein (λ_Exc_ = 488 nm, λ_Em_ = 507 nm)	[69], [70]
pKK-mClover3-TEV	N-terminus	green fluorescent protein (λ_Exc_ = 506 nm, λ_Em_ = 518 nm)	[71] Addgene ID: 74252
pKK-mCherry-TEV	N-terminus	red fluorescent protein (λ_Exc_ = 587 nm, λ_Em_ = 610 nm)	[72]
pKK-mRuby3-TEV	N-terminus	red fluorescent protein (λ_Exc_ = 558 nm, λ_Em_ = 592 nm)	[71] Addgene ID: 74252
pKK-mCardinal-TEV	N-terminus	red-shifted fluorescent protein (λ_Exc_ = 604 nm, λ_Em_ = 659 nm)	[73] Addgene ID: 56172
pKK-CyOFP1-TEV	N-terminus	orange-red fluorescent protein which has large Stokes shift λ_Exc_ = 497, 523 nm, λ_Em_ = 589 nm (excitation is quite efficient in the 485–525 range)	[74] Addgene ID: 74279
pKK-Dendra2N-TEV	N-terminus	green fluorescent protein that can be irreversibly photoconverted to a red (mCherry-like) state by irradiation with 405 nm or 440 nm light green state: λ_Exc_ 490 = nm, λ_Em_ = 507 nm; red state: λ_Exc_ 553 = nm, λ_Em_ = 573 nm	[75], [76], [77]
pKK-TEV-FLAG	C-terminus	see above	
pKK-TEV-3XFLAG	C-terminus	see above	[78], [79], [80]
pKK-TEV-HA	C-terminus	see above	
pKK-TEV-BirA-FLAG	C-terminus	see above	
pKK-TEV-MBP	C-terminus	see above	
pKK-TEV-ProteinA	C-terminus	two IgG binding domains of *Staphylococcus aureus* Protein A	[81], [82]
pKK-TEV-mTagBFP	C-terminus	see above	
pKK-TEV-mCerulean	C-terminus	see above	
pKK-TEV-mVenus	C-terminus	see above	
pKK-TEV-mAmber	C-terminus	see above	
pKK-TEV-EGFP	C-terminus	see above	
pKK-TEV-mEGFP	C-terminus	see above	
pKK-TEV-mClover3	C-terminus	see above	
pKK-TEV-mCherry	C-terminus	see above	
pKK-TEV-mRuby3	C-terminus	see above	
pKK-TEV-mCardinal	C-terminus	see above	
pKK-TEV-CyOFP1	C-terminus	see above	
pKK-TEV-Dendra2N	C-terminus	see above	
**pKK-BI16 Vector Series**			
pKK-BI16-TEV-mCherry	C-terminus	one protein is expressed with mCherry fusion tag, cleavable by TEV protease; second tag introduced by user	
pKK-BI16-FLAG-3C-ORF1_mClover3-TEV-ORF2	both at N-terminus	one protein expressed with FLAG fusion tag, cleavable by 3C protease (PreScission) or enterokinase; second protein expressed with mClover3 fusion tag, cleavable by TEV protease	
pKK-BI16-ORF1-3C-mRuby3_ORF2-TEV-mClover3	both at C-terminus	one protein expressed with mRuby3 fusion tag, cleavable by 3C protease (PreScission); second protein expressed with mClover3 fusion tag, cleavable by TEV protease	
pKK-BI16-ORF1-3C-FLAG_ORF2-TEV-mClover3	both at C-terminus	one protein expressed fusion with FLAG fusion tag, cleavable by 3C protease (PreScission); second protein expressed with mClover3 fusion tag, cleavable by TEV protease	
**pKK-BiFC Vector Series**			
pKK-BiFC-Venus	C-terminus	fragments of Venus (VN173, VC155) become fluorescent upon reconstitution	[83], [84], [85] Addgene ID: 22010 and 22011
**pKK-FRET Vector Series**			
pKK-FRET-ORF1-3C-Cerulean_ORF2-TEV-Venus	both at C-terminus	one protein expressed with Cerulean fusion tag, cleavable by 3C protease (PreScission); second protein expressed with Venus fusion tag, cleavable by TEV protease	
pKK-FRET-ORF1-3C-Cerulean_ORF2-TEV-Amber	both at C-terminus	one protein expressed with Cerulean fusion tag, cleavable by 3C protease (PreScission); second protein expressed with Amber fusion tag, cleavable by TEV protease	
**pKK-RNAi Vector Series**			
pKK-RNAi-nucEGFPmiR-FLAG-TEV	N-terminus	miRNA expression cassette with nuclear EGFP marker; N-terminal FLAG tag	
pKK-RNAi-nucEGFPmiR-mCherry-TEV	N-terminus	miRNA expression cassette with nuclear EGFP marker; N-terminal mCherry tag	
pKK-RNAi-nucCHERRYmiR-FLAG-TEV	N-terminus	miRNA expression cassette with nuclear mCherry marker; N-terminal FLAG tag	
pKK-RNAi-nucCHERRYmiR-EGFP-TEV	N-terminus	miRNA expression cassette with nuclear mCherry marker; N-terminal EGFP tag	
pKK-RNAi-nucEGFPmiR-TEV-FLAG	C-terminus	miRNA expression cassette with nuclear EGFP marker; C-terminal FLAG tag	
pKK-RNAi-nucEGFPmiR-TEV-mCherry	C-terminus	miRNA expression cassette with nuclear EGFP marker; C-terminal mCherry tag	
pKK-RNAi-nucCHERRYmiR-TEV-FLAG	C-terminus	miRNA expression cassette with nuclear mCherry marker; C-terminal FLAG tag	
pKK-RNAi-nucCHERRYmiR-TEV-EGFP	C-terminus	miRNA expression cassette with nuclear mCherry marker; C-terminal EGFP tag	
**pKK-RNAtag Vector Series**			
pKK-RNAtag-3UTR-24MS2SL	3'-terminus	24 repeats of the MS2 stem-loops to be attached to the 3' end of RNA expressed from the plasmid. RNA can be localized in cells upon cotransfection with fusion of MS2 with fluorescent protein.	
pKK-RNAtag-nTER-HA-TEV	N-terminus	short arginine-rich N-terminal domain (amino acids 1–22) of the bacteriophage λ transcriptional antiterminator protein N, a 12 amino-acid linker and HA peptide	[49]

A full description of the vectors can be found in S7 Supporting Information. In all vectors BshTI and NheI restriction enzymes are used for vector linearization before SLIC cloning according to our universal protocol. Expression of transgenes in all vectors is under control of a tetracycline repressor regulated promoter.

To extend the variety of possible functional analyses we also devised a vector with a bidirectional promoter which enables simultaneous, inducible expression of two introduced genes (Fig 1C). To this end we used the BI16 plasmid [46], a derivative of pcDNA5/FRT/TO with the original CMV promoter duplicated back-to-back. The tetracycline operator sequence was maintained so that transcription in both directions is regulated by the tetracycline repressor. We removed the luciferase coding sequences from BI16 and redesigned the cloning sites. As a result, we created a series of pKK-BI16 vectors (Table 1 and S7 Supporting Information). They utilize the cloning strategy described above on one side of the bidirectional promoter and have a traditional multiple cloning site on the other (Fig 1C, bottom diagram). These vectors should significantly simplify construction of plasmids for expression of two independent coding sequences. The pKK-BI16 vectors were further modified to obtain the pKK-RNAi, pKK-BiFC, and pKK-FRET vector series (Table 1 and S7 Supporting Information), which are intended for functional studies and *in vivo* protein interaction studies using bimolecular fluorescence complementation or Förster resonance energy transfer approach.

### Efficiency of the cloning procedure

**Fig 2:**
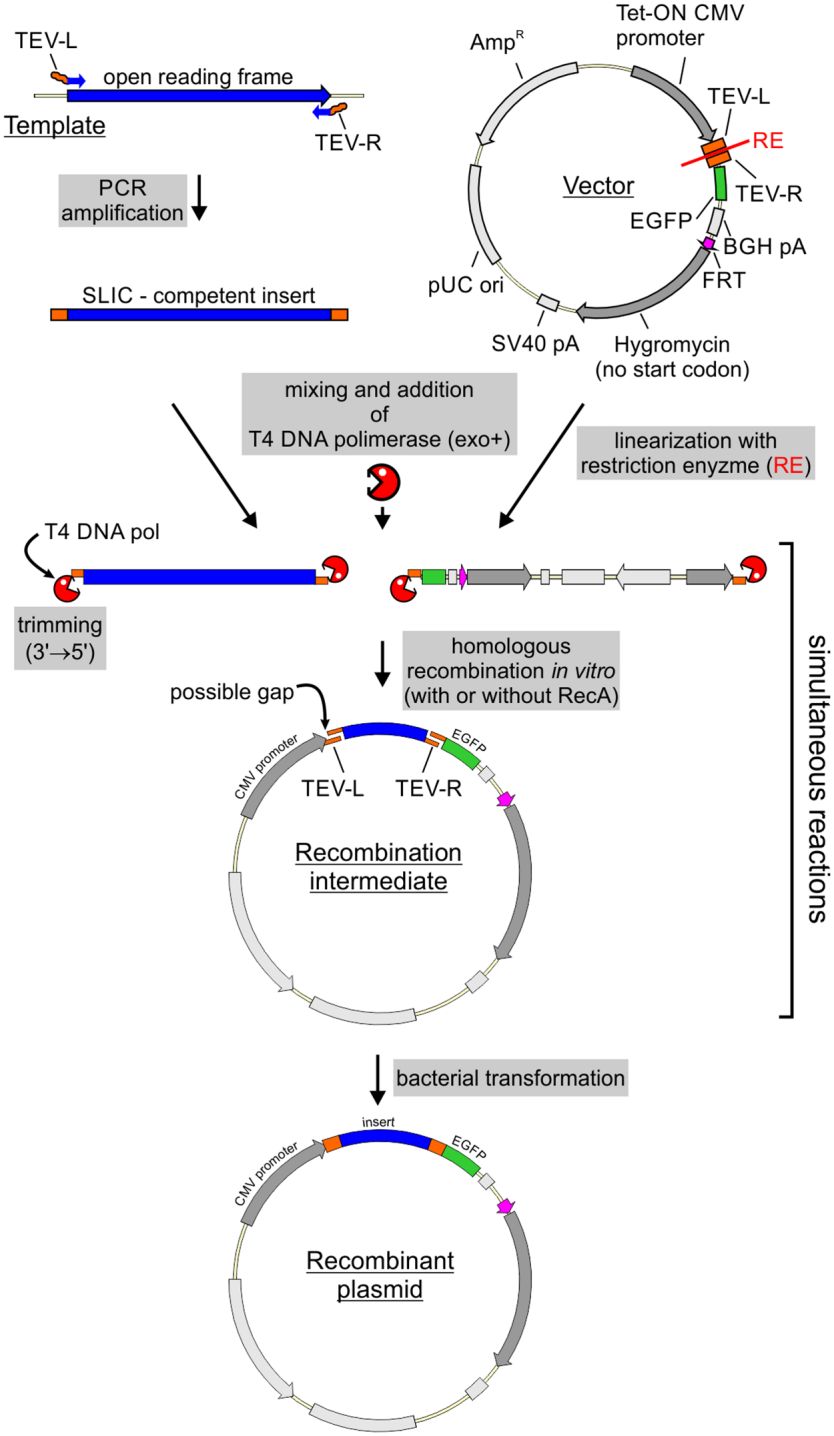
SLIC-based DNA cloning strategy. See main text for detailed description. RE–restriction enzymes used for vector linearization. These are BshTI and NheI in our protocol for universal SLIC. EGFP is an example of tag that can be used. A detailed protocol for the SLIC procedure can be found in S2 Supporting Information.

The cloning procedure starts with PCR amplification of a DNA fragment to be cloned using primers with overhangs complementary to the SLIC arms in the vector (Fig 2). The PCR product has to be purified from nucleotides, which inhibit the subsequent SLIC reaction. The purification method depends on the specificity of the PCR reaction; all unspecific PCR products must be removed. After purification the product is mixed with a linearized vector and treated with T4 DNA polymerase. In the absence of nucleotides the polymerase trims 3’ ends of DNA, producing sticky ends both in the vector and the PCR product (Fig 2). The sticky ends hybridize and form a nicked, potentially gapped, DNA molecule. After introduction to bacteria such lesions are repaired by the host system (Fig 2).

In the course of our studies we used this strategy to clone 155 different protein coding sequences to pKK series vectors, some with several different tags, bringing the total number of constructed plasmids to 456, a number high enough to assess the efficiency of our strategy. In the analysis of cloning efficiency we considered the kind of PCR template (plasmid versus cDNA) and the number of constructs produced that we were able to obtain easily. The first attempt was successful in 99% of cases with a plasmid template and in 75% of cases with a cDNA template. Overall 84% of constructs were successfully obtained on the first attempt and the remaining 16% were not obtained at all. This failure resulted mostly from unsuccessful PCR amplification of the insert (59 cases out of 72 unsuccessful cloning attempts). Thus, some constructs require additional optimization steps to produce the insert. Nevertheless, the analysis demonstrates the high efficiency of our SLIC strategy and points to insert preparation as the limiting step of the procedure.

### New vectors are competent for stable cell line generation

**Fig 3:**
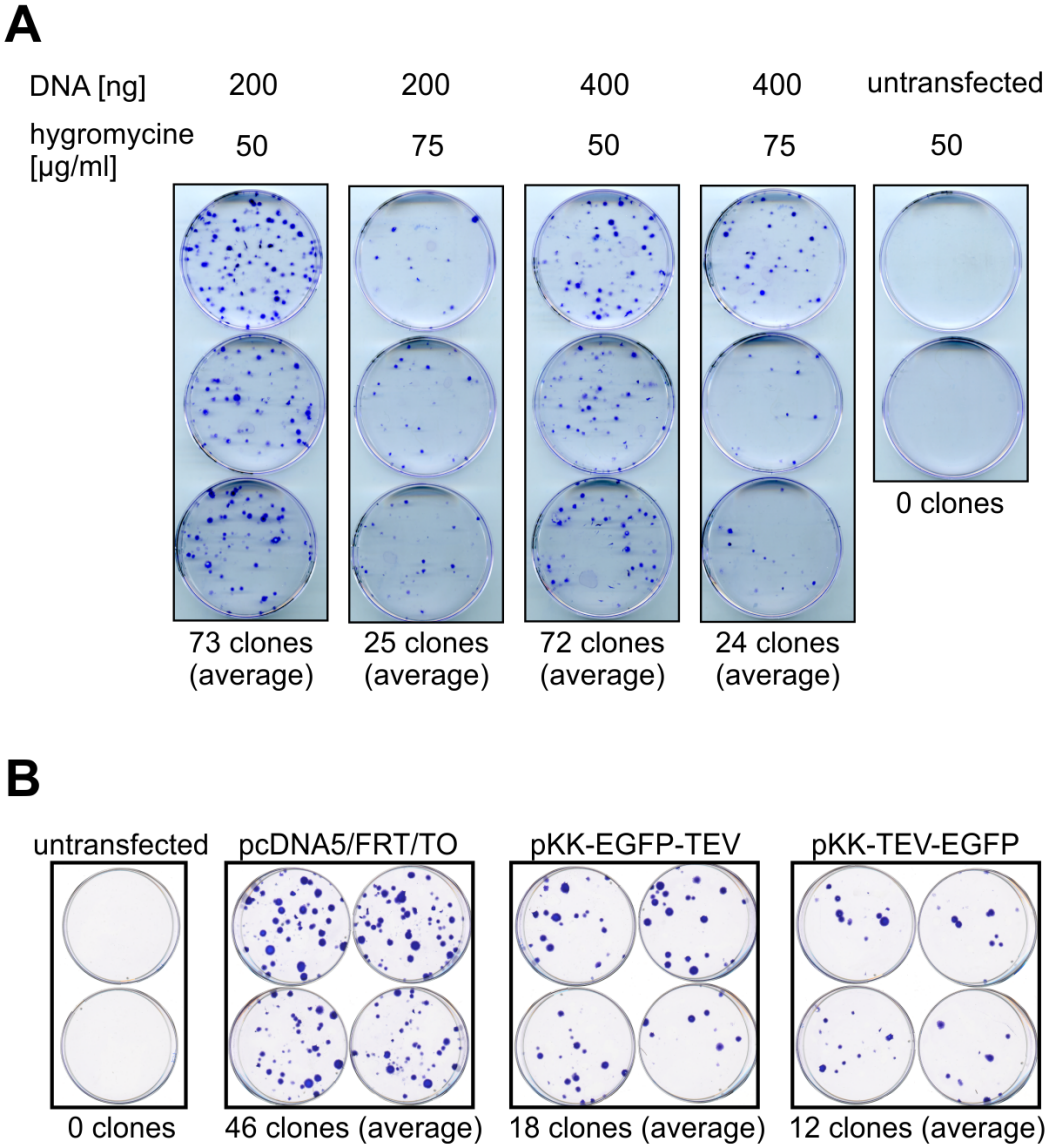
Efficiency of stable cell line generation. (A) Influence of plasmid quantity and selection stringency on the number of colonies obtained following stable transfection of 293 Flp-In T-REx cells. 1.0 μg of pOG44 was mixed with the indicated amounts of pcDNA5/FRT/TO and used for transfection. Cells were selected by treatment with the indicated concentration of hygromycin B and constant concentration of blasticidin S (10 μg/ml). Colonies were stained with crystal violet. (B) Comparison of stable transfection efficiency with pcDNA5/FRT/TO or its pKK derivatives. Cells were transfected with 300 ng of indicated plasmids and 1.0 μg of pOG44 and subjected to selection with hygromycin B (50 μg/ml) and blasticidin S (10 μg/ml).

We set out to examine whether the modifications to the pcDNA5/FRT/TO backbone interfere with stable cell line generation. In order to establish a baseline for FLP-mediated integration efficiency, we transfected the 293 Flp-In T-REx parental cell line with the original pcDNA5/FRT/TO vector and counted the number of colonies that originated from cells that had undergone FRT-targeted plasmid integration and became resistant to the selection antibiotic. We tested a range of selection antibiotics concentrations as well as the amounts of the targeting vector used for transfection (Fig 3A). We found that decreasing the antibiotic concentration yields more colonies, while providing enough selective pressure to kill off non transfected cells (Fig 3A). As for DNA amount, we found that there was no effect on the number of colonies obtained between the two tested concentrations (Fig 3A).

Having optimized transfection and selection conditions, we compared stable transfection efficiency between the original vector and our vectors bearing N- or C-terminal EGFP tags (pKK-EGFP-TEV and pKK-TEV-EGFP, respectively). We found that our vectors produced a lower number of colonies (Fig 3B), however, this number is enough to establish functional stable cell lines, as evidenced by our further experiments (see below). We also observed that the number of colonies obtained for a given plasmid can vary significantly from transfection to transfection (72 versus 46 colonies for pcDNA5/FRT/TO in Fig 3A and 3B, respectively), which was not due to any obvious reasons like different plasmid preparations or number of cells subjected to transfection.

**Fig 4:**
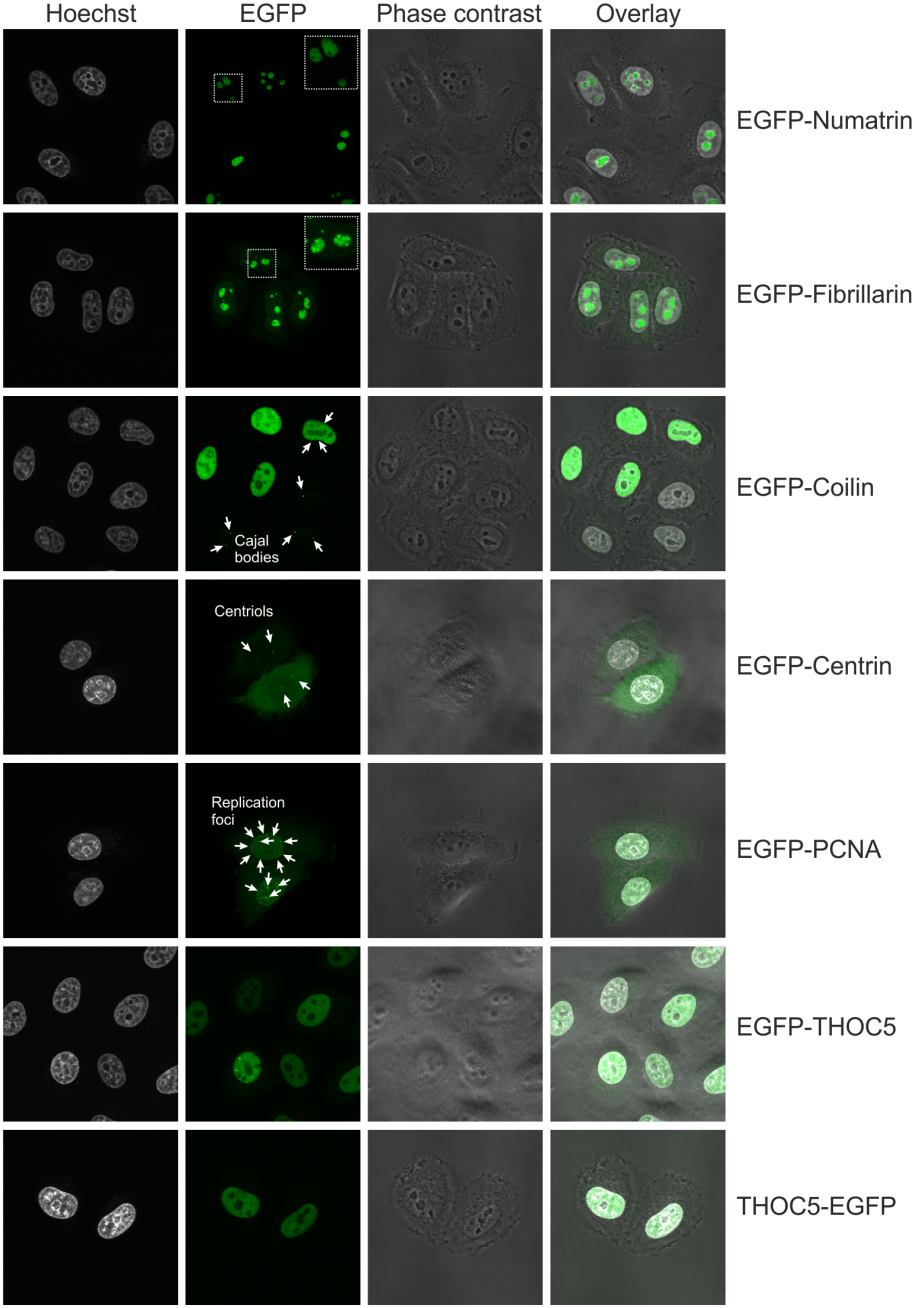
Intracellular localization of EGFP tagged proteins. Live cell imaging of HeLa-derived stable cell lines expressing EGFP fusions of the indicated proteins. Nuclei were stained with Hoechst 33342.

Subsequently, we verified that DNA constructs created from our vectors are suitable for stable transfection. For this purpose, we obtained several constructs encoding EGFP-tagged proteins with different subcellular localization and used them for stable transfection of 293 and HeLa parental cell lines. We studied the localization of the tagged proteins by live cell imaging (Fig 4 for HeLa and S2 Fig for 293). In agreement with previous reports [86], [87], [88], numatrin and fibrillarin each localized to nucleoli (Fig 4 and S2 Fig). Potential differences in the respective sub-nucleolar localizations exhibited by each protein were consistent with previous reports [86], [87], [88]. Coilin produced foci within the nucleus (Fig 4 and S2 Fig) consistent with the expected localization of the protein to Cajal bodies [89], [90]. In cells that express the fusion to higher levels a diffuse nuclear localization was observed which is in agreement with previous report showing that overexpression of coilin disrupts Cajal bodies [91]. A punctate signal in the cytoplasm was observed for centrin (Fig 4 and S2 Fig), a known component of the centrosome [92], [93]. The proliferating cell nuclear antigen protein (PCNA), which functions as a scaffold for the DNA replication machinery, localized to replication foci (Fig 4 and S2 Fig), as expected [94]. THOC5, a component of the THO complex involved in transcription and RNA export [95], was found to localize to the nucleus regardless of the tagged end (Fig 4 and S2 Fig).

### Regulation of transgene expression

**Fig 5:**
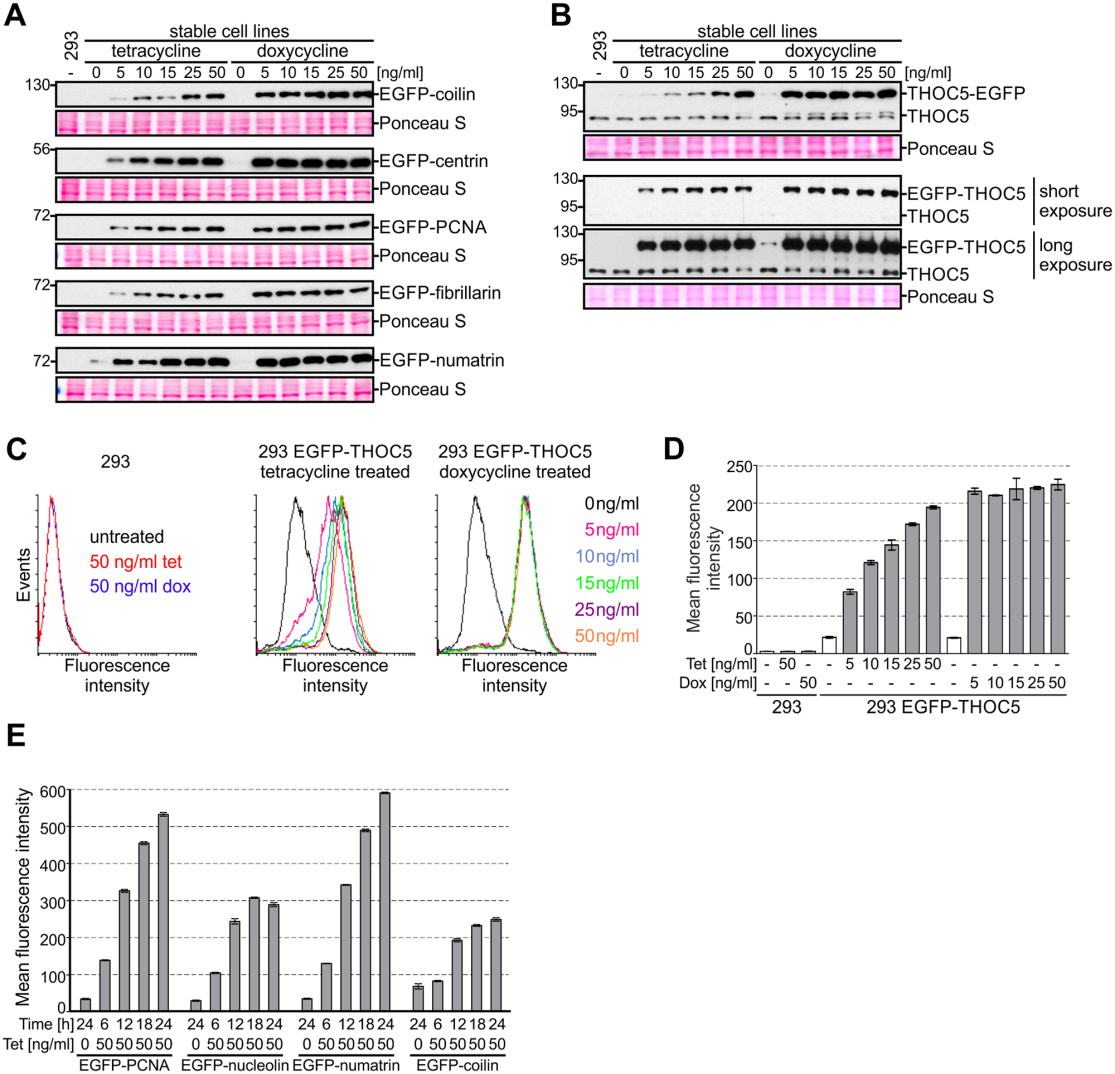
Comparison of gene expression inducers. (A-D) Cells were treated with different concentrations of tetracycline or doxycycline and gene expression was monitored by western blot (A: anti-EGFP, B: anti-THOC5 antibodies, Ponceau S staining of the membrane was performed as a loading control) or flow cytometry (C, D: EGFP fluorescence). (D) Quantitative representation of data shown in panel C. Data are represented as mean ± SD (n = 3). (E) Analysis of the kinetics of expression of the indicated transgenes. Cells were treated with tetracycline, collected after indicated time and analyzed by flow cytometry. Mean fluorescent intensity of EGFP positive cells is shown (mean ± SD, n = 3).

The strong CMV promoter, often used to drive transgene transcription and present in our vectors, ensures high transcription levels. This in turn can lead to massive overexpression [96], which may result in artefacts such as protein mislocalization; hence it is of great importance to be able to regulate transgene expression. Here, we use one of the most common inducible gene expression systems, wherein transcription is controlled by elements of the tetracycline resistance operon, which relies on tetracycline or its derivative, doxycycline, as inducers [97]. Notably, this regulatory system not only allows switching the transgene on or off, it also grants a certain degree of quantitative control [96], [97]. To determine to what extent gene expression can be regulated, we induced transgenes with a range of concentrations of tetracycline and doxycycline as they are both widely used in the literature (Fig 5).

Stable 293 cell lines producing different proteins fused to EGFP at the N-terminus were treated with the inducer for 24 hours and collected for western blot analysis. The levels of fusion proteins were assessed using anti-EGFP antibodies (Fig 5A). We found that the level of the protein of interest was the same at all doxycycline concentrations tested, whereas tetracycline yielded a dose-dependent, albeit not always linear response (Fig 5A). The maximal level of expression induced with tetracycline was similar to that observed for cells treated with the lowest concentration of doxycycline (Fig 5A). This suggested that within the tested concentrations range, tetracycline enables better fine-tuning of transgene expression. To examine this issue further, we analysed expression of THOC5 in N- or C-terminal fusion with EGFP (Fig 5B–5D).

We performed western blot analysis with THOC5- and EGFP-specific antibodies to compare levels of endogenous and tagged protein (Fig 5B). In addition, we took advantage of the fluorescent tag to measure transgene expression with flow cytometry (Fig 5C and 5D). Unlike western blot, where the measured signal reflects the population average, flow cytometry gives quantitative output at the single cell level, and therefore can extract information on population homogeneity and discern whether the overall increase in steady-state levels of a protein results from a small increase across the whole population or a large increase in a fraction of cells. The cytometry results were in line with the western blot data: a dose-dependent response was observed for tetracycline but not doxycycline (Fig 5B–5D). Importantly, we found that expression changes on a per cell basis rather than per population basis, that is the growing concentration of tetracycline causes each cell to express more protein rather than causing a growing fraction of the population to turn on expression at maximum capacity (Fig 5C and 5D).

Within the tested concentration range, our results indicate that tetracycline is superior to doxycycline for studies that require adjustment of transgene expression. On the other hand, if massive overproduction is needed, doxycycline has the advantage. It is worth noting that for some transgenes it may be difficult to tune their expression to the levels comparable to their endogenous counterparts. For example, we were able to achieve the endogenous level of expression for THOC5-EGFP but not for EGFP-THOC5 (Fig 5B).

The difference in biological activity of the examined inducers can be related to their different affinity to TetR [43] and/or their different stability [98]. We tested differently aged tetracycline solutions and found that while induction efficiency deteriorates with prolonged storage, reliable, reproducible results can be obtained with solutions as old as 40 days (S3 Fig). Moreover, we examined a wide range of tetracycline and doxycycline concentrations to see if they affect 293 cell viability (S4 Fig). We did not found deleterious effect of tetracycline or doxycycline treatment, even at concentrations as high as 10 μg/ml, which is two orders of magnitude above the normal working range of up to 0.1 μg/ml (S4 Fig).

Next, we performed a time-course experiment in order to monitor transgene expression over time. To this end, we used HeLa stable cell lines expressing EGFP-tagged proteins and monitored them using flow cytometry, so that population homogeneity could also be tracked. Four different transgenes were analysed (Fig 5E). Expression of all studied transgenes is evident after 6 hours of induction and increases with time until the maximum is achieved at about 24 hours after induction (Fig 5E). During that time cells respond at different rates, *i*.*e*. population homogeneity can vary, but by the time maximum expression is achieved, maximum homogeneity is as well. Notably, for all tested cells lines, a fraction of cells that do not express the transgene exists. This fraction appears to depend solely on the transgene under investigation and can be minimized by cell sorting, but rebuilds over time (data not shown).

**Fig 6:**
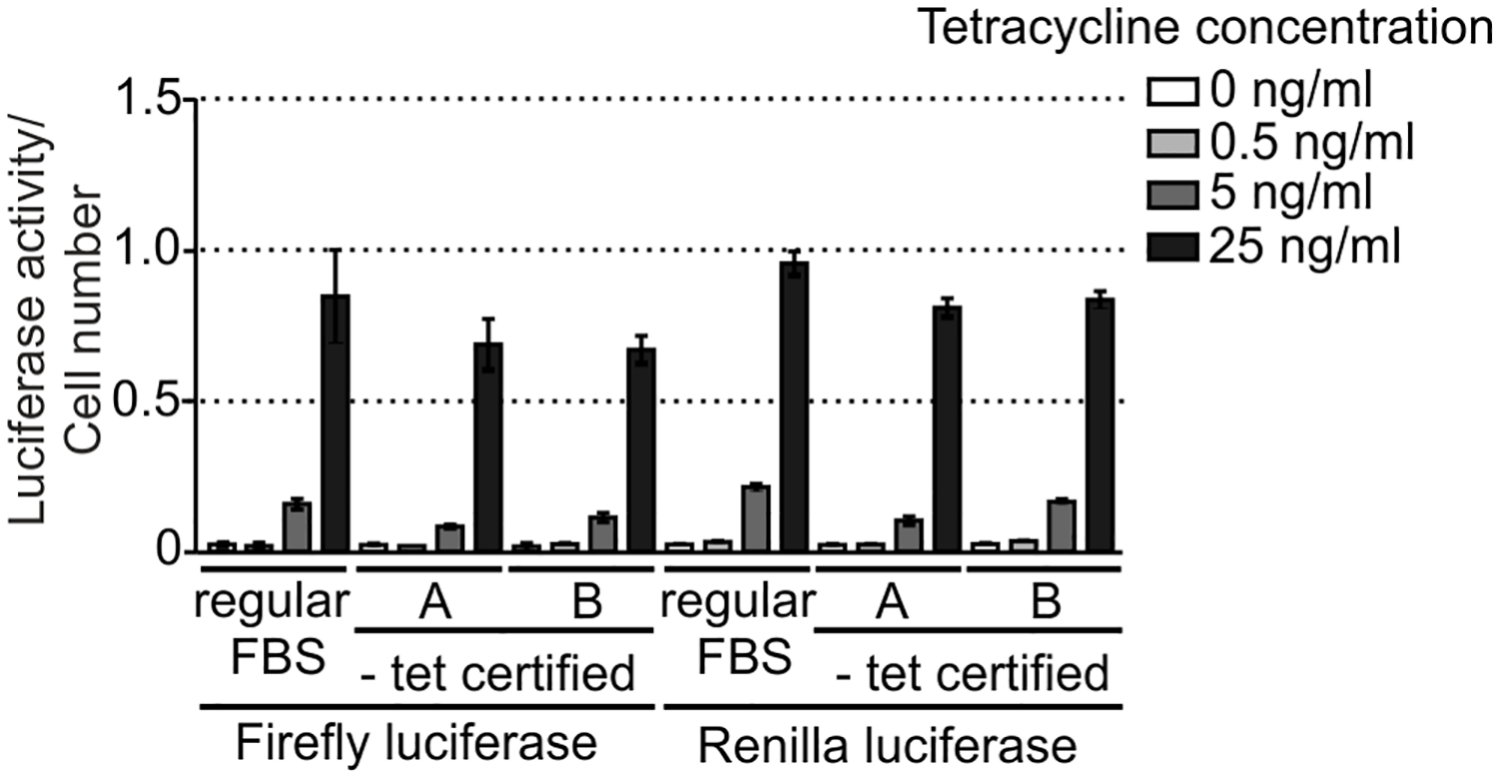
Influence of different FBS on transgene expression. 293 Flp-In T-REx cells stably transfected with a plasmid encoding firefly and renilla luciferase under control of a TetR-regulated bidirectional promoter were cultured in medium supplemented with different fetal bovine sera (FBS), and transgene expression was assessed by measurement of luciferase activity. Two FBS certified for absence of tetracycline or its derivatives were compared to regular FBS. Cells were treated with the indicated concentrations of tetracycline to measure induction response on different sera. Luciferase activity was normalized to the number of cells, which was assessed using AlamarBlue. Data are represented as mean ± SD.

The use of an inducible gene expression system is of great importance for studies of transgenes, the expression of which can affect cell fitness and viability. In these cases, it is crucial to keep transgene expression as low as possible under non-induced state. A common concern is that serum can contaminate culture media with tetracycline or its derivatives, and it is thus usually recommended that specially tested tetracycline-free grade (Tet-free) FBS is used. Such serum is much more expensive than regular one and can greatly increase the cost of prolonged or large-scale culture. We decided to test the alleged superiority of Tet-free FBS to regular FBS in terms of the basal level of transgene expression. To this end, we generated stable 293 Flp-In T-REx cells expressing firefly and renilla luciferase and assessed the activity of the enzymes in uninduced cells cultured in media prepared with regular FBS or certified Tet-free FBS obtained from two different vendors. As a positive control, we treated cells with three different concentrations of tetracycline, including 25 ng/ml, which resulted in a maximum transgene expression in our previous experiments. We did not observe any differences in basal expression of the transgenes (Fig 6), but we did find differences in induced expression: cells cultured in medium supplemented with regular FBS achieved higher transgene expression upon induction with 5 and 25 ng/ml tetratycline than likewise treated cells cultured in medium containing Tet-free FBS. The obtained results suggest that there is no clear benefit from using Tet-free FBS in terms of leaky expression. However, it is important to note that FBS can vary from batch to batch and it is prudent to pre-test every lot of standard FBS for its suitability for tetracycline-based expression systems.

### Vectors for simultaneous expression of mRNA and miRNA

One of the most important approaches in discovering gene function is its downregulation so that the respective protein product is depleted. This can be achieved by several experimental strategies that differ in downregulation efficiency and the effort required. A complete inactivation of a gene by its deletion or insertional inactivation has a strong advantage in terms of downregulation but, if the gene of interest is essential, requires a conditional–or inducible–knock-out, which is quite laborious. Alternatively, expression of the gene can be downregulated by RNA interference (gene silencing). This is a straightforward strategy, in which short RNA molecules complementary to a particular mRNA target it for degradation or repress its translation [99]. Unlike gene disruption, this approach can target specific isoforms but has a major drawback in the risk of off-target activity of the short RNA [100]. Therefore, in this kind of experiments it is very important to introduce controls that confirm that the observed phenotypes are *bona fide* effects of downregulation of the gene of interest. One such control is a “rescue” sample, in which an ectopic, RNAi-resistant allele of the gene in question is expressed, while the endogenous one is silenced. A simple rescue sample is obtained by transient co-transfection with siRNA and plasmid DNA, however, this may suffer from a range of problems, like irreproducibility, imperfect co-transfection, and transfection-related cell stress.

**Fig 7:**
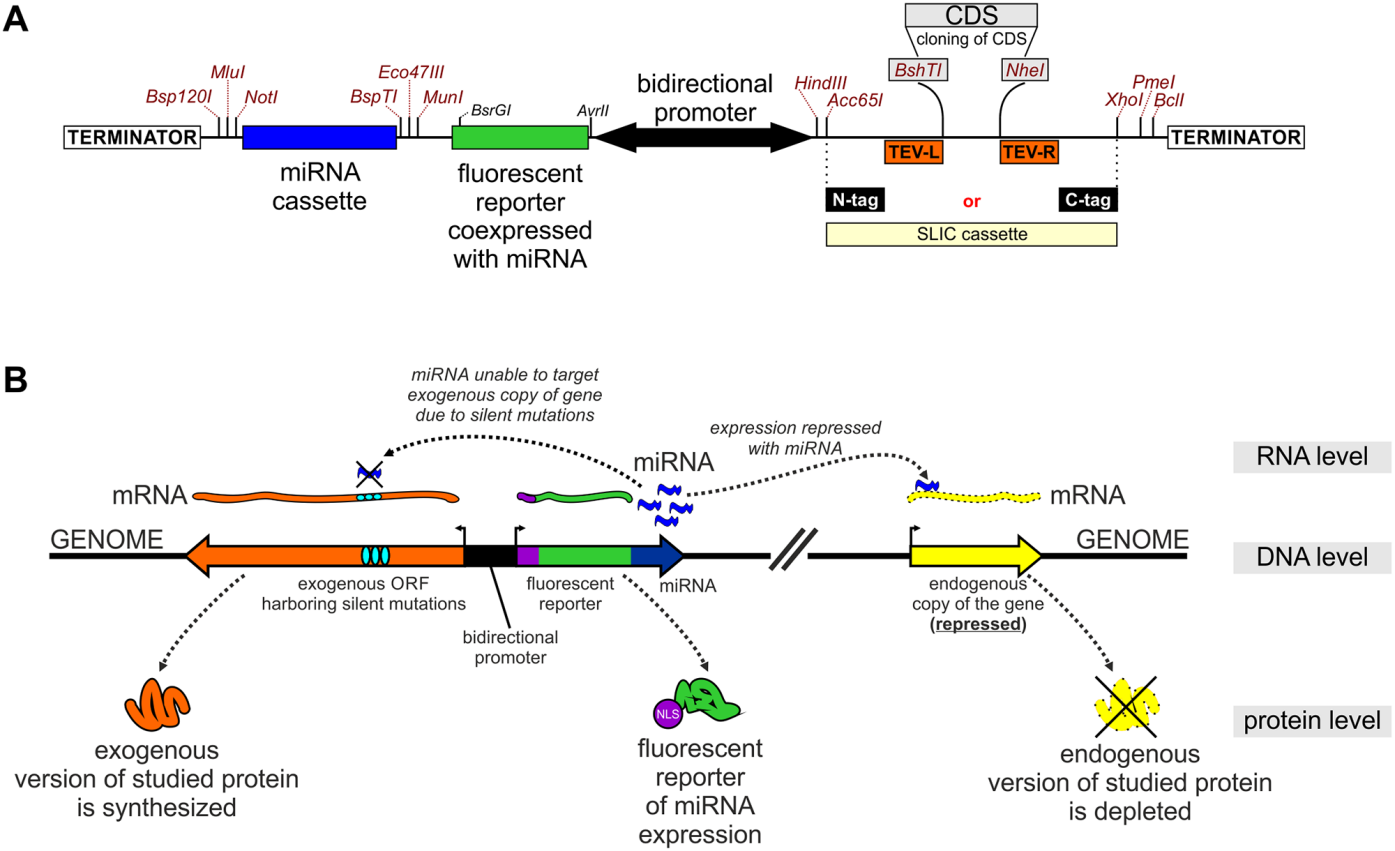
pKK-RNAi vectors as a tool for generation of a cellular model for functional studies. (A) Diagram of cloning regions. Potentially useful unique restriction sites are marked. (B) Principles of the approach. Three levels of gene expression are shown. The plasmid integrated into a genome contains: 1) a gene for miRNAs that target the mRNA of a gene of interest; 2) an allele of the gene of interest where the CDS contains silent mutations so that it is insensitive to the miRNAs. As a result the endogenous version of the protein of interest is depleted whereas its ectopic form expressed. NLS marks a nuclear localization signal.

To create a straightforward tool for RNAi-based rescue experiments, we designed the pKK-RNAi vector series (Table 1, Fig 7A), derived from pKK-BI16. The logic behind these vectors was previously described by us in studies concerning the catalytic subunits of the exosome complex [101], [102] and other nucleases [103], [104]. We use plasmids with a bidirectional promoter in order to concurrently express two genes: 1) a cassette encoding miRNAs that target the gene of interest, and 2) an allele of the gene of interest with the protein coding sequence harbouring silent mutations that make the mRNA insensitive to the miRNAs (Fig 7B). As a result, the endogenous alleles of the gene are silenced, whereas the ectopic copy is expressed (Fig 7B). Furthermore, the miRNAs are cotranscriptionally expressed with a fluorescent protein reporter so that analysis can be narrowed down to cells that express miRNA, provided that applied assay can distinguish cells expressing reporter (Fig 7).

**Fig 8:**
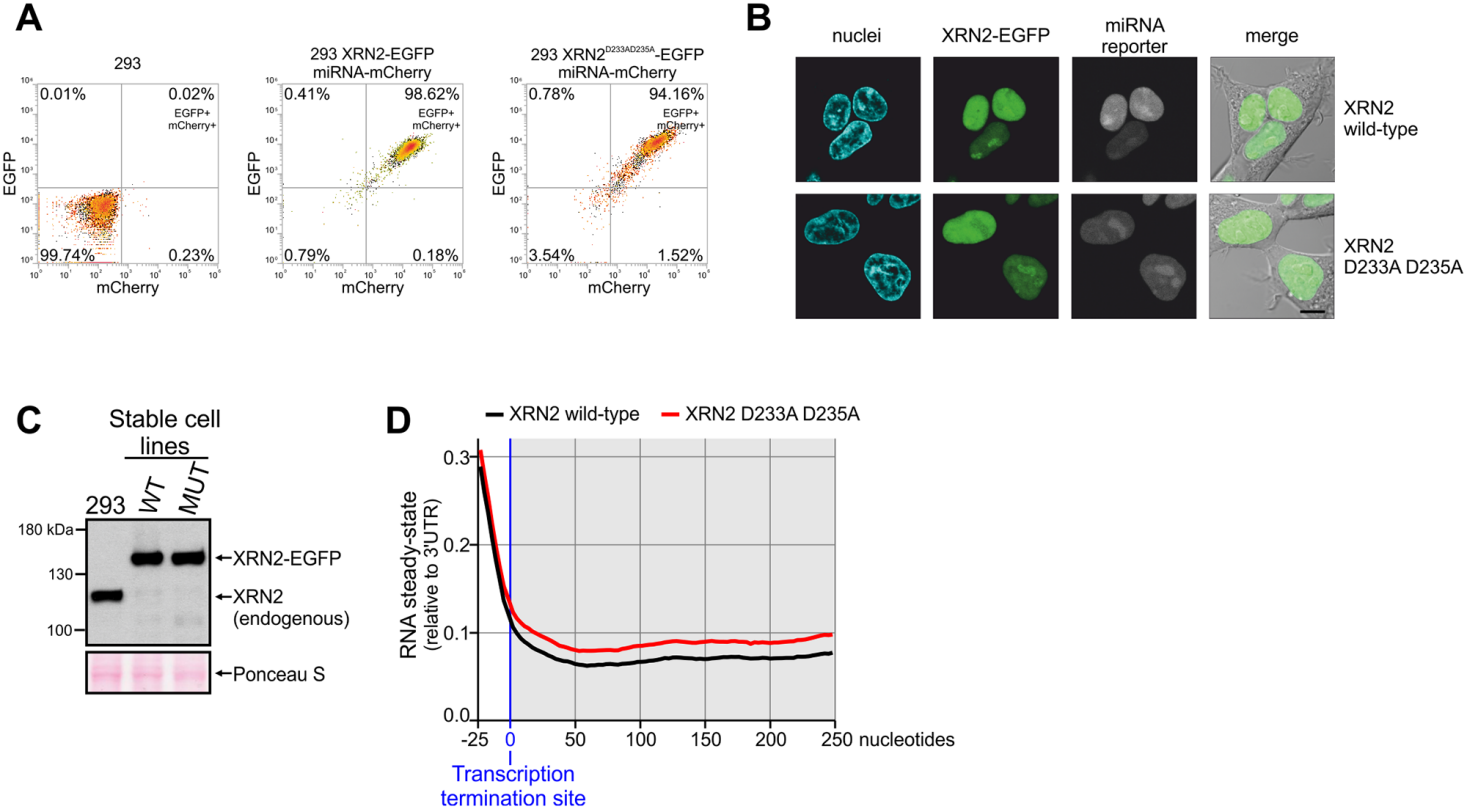
Involvement of XRN2 in transcription termination. (A) Flow cytometry measurement of transgenes expression after 24 hours of induction (EGFP tags XRN2, mCherry is a reporter of miRNA expression). (B) Confocal live cell imaging of EGFP tagged XRN2 and Hoechst 33342 stained nuclei. (C) Western blot analysis of XRN2 protein with anti-XRN2 antibodies. Parental 293 cells and their derivatives analyzed in panel A and B were treated with tetracycline for 72 hours and subjected to western blot. Ponceau S staining of the membrane was performed as a loading control. (D) Meta-gene analysis of transcriptional read-through in wild-type and mutant XRN2 cells. Strand-specific read densities were averaged across 250-bp genomic windows placed directly downstream of 3' ends of highly expressed (TPM > 10), spliced transcripts. The signal is normalized to the average expression detected in the last 250 nt of the analyzed transcripts (250-bp windows upstream to the expected termination site). The shaded part of the graph marks transcripts downstream of transcription termination site (products of transcriptional read-through). It is important to note that lines representing RNA steady-state levels overlay in the part of the graph which correspond to RNAs originating from the transcription upstream of the transcription termination site. This is in contrast to the part of the graph which represent RNA resulting from the unsuccessful transcription termination (shaded part of the graph).

The pKK-RNAi vectors apply a cloning procedure (S5 Fig and S3 Supporting Information) that is simpler than the previous one [101], which involved multiple steps and was strongly dependent on the target sequences. Both inconveniences were reduced as much as possible in the new vectors. Notably, the place where the coding sequence of interest is inserted is common to all pKK vectors, so that, once prepared, an insert can be cloned into any vector. We also improved the utility of the miRNA expression reporter by adding the SV40 nuclear localisation signal to the fluorescent reporter (Figs 7 and 8). It is now more concentrated and as such easier to detect with fluorescent microscopy (stronger fluorescent signal per area unit), and can be used as a nuclear marker which can be of great value for example for studies that involve image analysis. If necessary, the fragment encoding the miRNA expression reporter can be removed or substituted by restriction enzyme cloning.

We created several pKK-RNAi vectors with different miRNA reporters and fusion tags for the protein of interest (Table 1 and S7 Supporting Information). So far we have used these vectors to create 50 miRNA-encoding constructs, which were obtained by subcloning of the miRNA cassette, for 27 genes (Table 2). 45 of these constructs were further modified by inserting a CDS with silent mutations (Table 3). This step was performed by splice-PCR, which is thoroughly described in Supplementary Data 4. The majority of constructs were obtained on the first attempt (Tables 2 and 3), which highlights the high efficiency of our cloning procedure (S5 Fig and S3 Supporting Information). All subcloning of miRNA cassettes required only one attempt to obtain the correct construct (Table 2), whereas in the case of CDS cloning the first attempt was successful almost 90% of the time (Table 3). Notably, the number of plasmids that had to be sequenced to obtain the correct construct indicates that mutations introduced by PCR are not the rate-limiting step (Tables 2 and 3).

**Table 2:** Efficiency of the miRNA cassette subcloning into pKK-RNAi vectors.

Constructs attempted	First attempt successful (fraction)	Correct sequence on first clone checked (fraction)
50	50 (100%)	50 (100%)

**Table 3:** Efficiency of CDS cloning into pKK-RNAi vectors.

Constructs attempted	First attempt at CDS assembly successful (fraction)	First attempt at SLIC reaction successful (fraction)	Correct sequence on *n-*th clone checked (fraction)					
			n = 1	n = 2	n = 3	n = 4	n = 5	n = 6
45	40 (89%)	42 (93%)	34 (75.5%)	5 (11%)	2 (4.5%)	2 (4.5%)	1 (2.25%)	1 (2.25%)

Splice-PCR was used for *in vitro* assembly of miRNA-insensitive coding sequences that were subsequently cloned into the pKK-RNAi vector by our universal SLIC protocol.

To test the pKK-RNAi cellular model in functional studies we analysed the effect of depriving the cell of the catalytic activity of the nuclear 5’ to 3’ exoribonuclease XRN2. Catalytic amino acids in this protein had been defined previously, so it was possible to design a mutated catalytically inactive form of the protein (XRN2^D233A-D235A^) [105]. We created 293 Flp-In T-REx stable cell lines that induciby silence endogenous XRN2, and concomitantly express wild-type or inactive XRN2 in fusion with EGFP at the C-terminus. Thus, complementation of silencing of endogenous XRN2 with the expression of mutant version of the protein allows to directly link potential phenotypes with the lack of XRN2 enzymatic activity. Flow cytometry analysis showed that almost all cells expressed the miRNA reporter (mCherry) and the ectopic protein (EGFP signal) (Fig 8A). We confirmed correct subcellular localization of the ectopically expressed proteins by confocal microscopy. This analysis revealed their anticipated nuclear localization (Fig 8B). Subsequently, we examined the efficiency of XRN2 downregulation at the protein level. It showed very efficient depletion of XRN2, which was hardly detectable (Fig 8C), while the ectopic forms of XRN2 were expressed, importantly, at levels similar to that of endogenous XRN2 in parental cells (Fig 8C).

It was shown that human XRN2 is a *bona fide* component of the transcription termination machinery [106], [107]. We checked if it is possible to reproduce this observation using our experimental system. To this end we isolated total RNA from tetracycline-treated cells, depleted it from rRNA and conducted strand-specific deep sequencing. A meta-gene analysis of the transcriptional read-through was performed (Fig 8D). In agreement with other reports [106], [107], we found that the ribonucleolytic activity of XRN2 is required for canonical RNAPII transcription termination. A clear accumulation of RNA downstream to the expected transcription termination site resulting from unsuccessful termination events was observed in cells that expressed inactive, miRNA-insensitive XRN2 instead of the endogenous protein (Fig 8D). This class of transcripts is clearly less abundant in cells that express the catalytically active form of XRN2. This indicates that the cellular model which we obtained using pKK-RNAi vectors is functional and faithfully reproduces independent experiments obtained by others using different methods. Taken together, our analysis of cloning efficiency and the performed functional studies prove that pKK vectors and our associated procedures allow for easy creation of reliable cellular models that can be used in determining protein function.

## Conclusions

We have created a series of vectors that facilitate various functional and biochemical studies of human proteins. The combination of an efficient DNA cloning strategy with the Flp-In system for stable cell line generation guarantees high utility of the vectors. The Flp-In system is widely used in different research areas, like mitochondria [108], [109], [110], [111], [112], RNA metabolism [113], [114], [115], proteomic studies [116], [117], [118], cell signaling [119], [120] and others [121], which calls for the existence of compatible vectors that ensure straightforward cloning. The pKK-RNAi vectors have a high potential of being particularly useful in functional analyses. They provide a simple way to substitute a protein with its engineered version. This can help to elucidate the function of particular parts of the protein, confirm pathogenic nature of newly identified mutations, or simplify rescue experiments in studies involving gene silencing. Moreover, in *in vivo* protein-protein interaction studies it can prove beneficial to deplete the endogenous form of the protein, which competes for interactors [122]. Furthermore, expressing miRNAs, rather than transient transfection with siRNAs, can produce a more homogenous cell population and result in a higher overall silencing efficiency. Still, one should keep in mind that all RNAi techniques may be less efficient in some cases, *e*.*g*. with highly abundant mRNAs or extraordinarily stable proteins.

In agreement with a previous report [97] we observed that doxycycline is more effective in gene induction than tetracycline. This can be detrimental when fine-tuning transgene expression is required, as small differences in the inducer concentration can cause significant differences in transgene expression. Although the expressed transgenes respond to tetracycline in a concentration-dependent manner, the expression level can be transgene-specific, which is likely related to the stability of particular fusion proteins or their mRNAs. Therefore, the response of each transgene should be tested; the concentrations we used can serve as a guideline. A dose-dependent response can likely be achieved with doxycycline as well but lower concentrations would have to be tested.

Using our experimental approach, we confirmed the involvement of XRN2 in canonical RNAPII transcriptional termination.

## Supporting information

S1 Fig Components and principle of the Flp-In system. (PDF)

S2 Fig Intracellular localization of EGFP tagged proteins in 293 cells. (PDF)

S3 Fig Analysis of the stability of tetracycline solutions. (PDF)

S4 Fig Viability test of 293 cells treated with tetracycline or doxycycline. (PDF)

S5 Fig Cloning strategy for pKK-RNAi vectors. (PDF)

S1 Supporting Information Annotated vector sequences in GenBank format. (TXT)

S2 Supporting Information Detailed protocol for the SLIC procedure. (PDF)

S3 Supporting Information Detailed protocol for designing the miRNA cassette, splice-PCR and cloning into pKK-RNAi vectors. (PDF)

S4 Supporting Information List of primers used for construction of plasmids used in the manuscript for stable cell line generation. (XLSX)

S5 Supporting Information Detailed protocol for stable cell line generation. (PDF)

S6 Supporting Information Maps of all reported vectors. (PDF)

S7 Supporting Information Full description of vectors. (XLSX)
