# Supplementary material for: Versatile approach for functional analysis of human proteins and efficient stable cell line generation using FLP-mediated recombination system
Source: PLoS One. 2018 Mar 28;13(3):e0194887. doi: 10.1371/journal.pone.0194887 (PMC5874048; doi:10.1371/journal.pone.0194887)
Supplement: S1 Fig — (PDF) [file pone.0194887.s001.pdf]

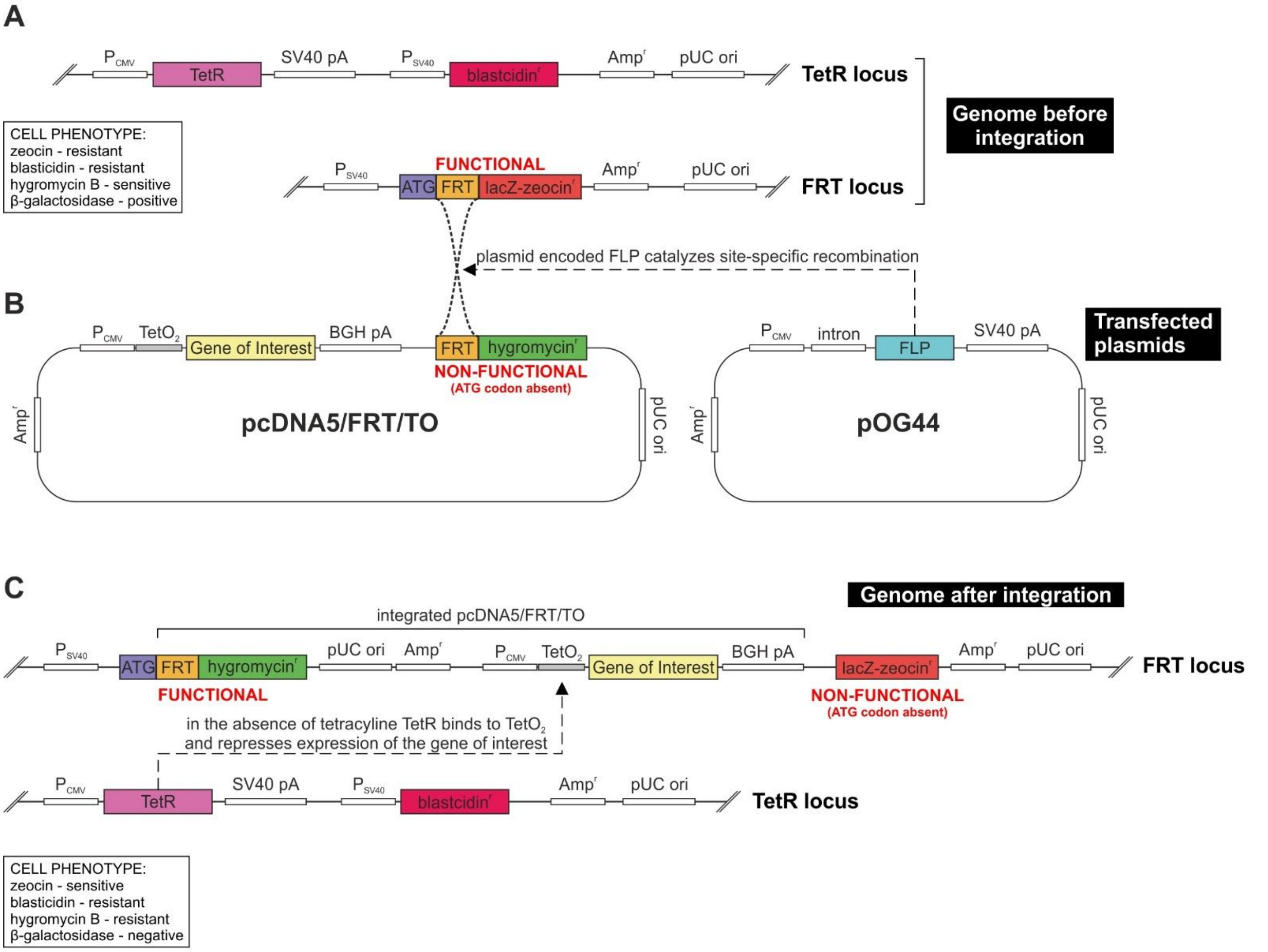

S1 Fig. Components and principle of the Flp-In system. (A) To enable FLP-mediated stable cell line generation and tetracycline regulation of transgene expression, the genome of the parental cells is pre-modified by stable integration of plasmids that bear the FRT sequence, which is recognized by FLP recombinase, and the tetracycline repressor (TetR) gene. (B) To generate a transgenic cell line the parental cells are transfected with pOG44 to express the FLP recombinase and a plasmid that contains the gene of interest and the FRT sequence. (C) After targeted integration into the genome expression of the gene of interest is repressed due to the activity of TetR, which binds to the promoter in the absence of tetracycline. This binding can be reversed by addition of tetracycline or doxcycline to the culture medium. (A, C) Note the differences in antibiotic resistance of the cells before and after targeted integration.
