# Supplementary material for: Versatile approach for functional analysis of human proteins and efficient stable cell line generation using FLP-mediated recombination system
Source: PLoS One. 2018 Mar 28;13(3):e0194887. doi: 10.1371/journal.pone.0194887 (PMC5874048; doi:10.1371/journal.pone.0194887)
Supplement: S2 Fig — (PDF) [file pone.0194887.s002.pdf]

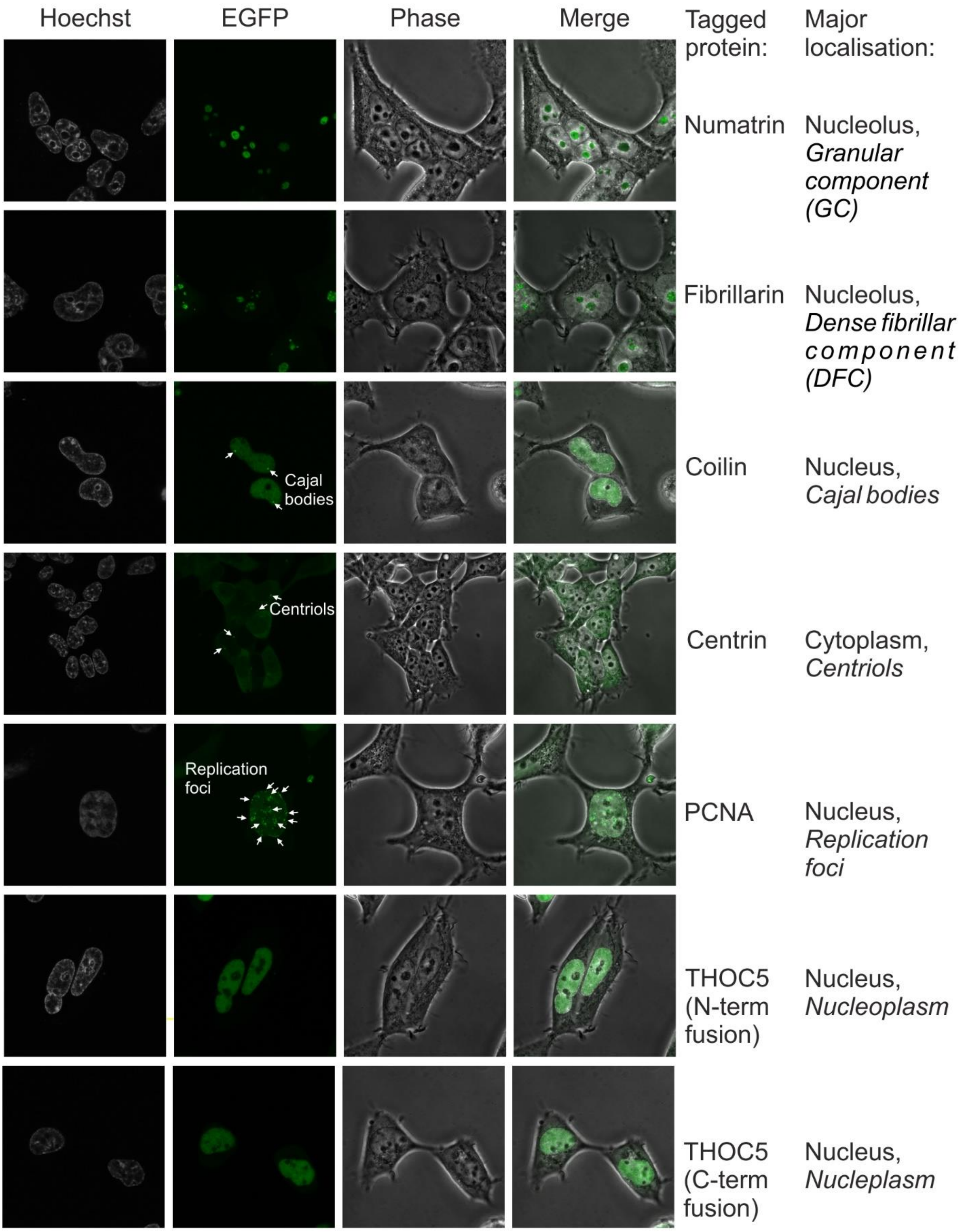

S2 Fig. Intracellular localization of EGFP tagged proteins in 293 cells. Live cell imaging of stable 293-derived cell lines, expressing EGFP fusions of the indicated proteins, by confocal microscopy. Nuclei were stained with Hoechst 33342. Major localizations of the proteins are noted.
