# Supplementary material for: Versatile approach for functional analysis of human proteins and efficient stable cell line generation using FLP-mediated recombination system
Source: PLoS One. 2018 Mar 28;13(3):e0194887. doi: 10.1371/journal.pone.0194887 (PMC5874048; doi:10.1371/journal.pone.0194887)
Supplement: S3 Fig — (PDF) [file pone.0194887.s003.pdf]

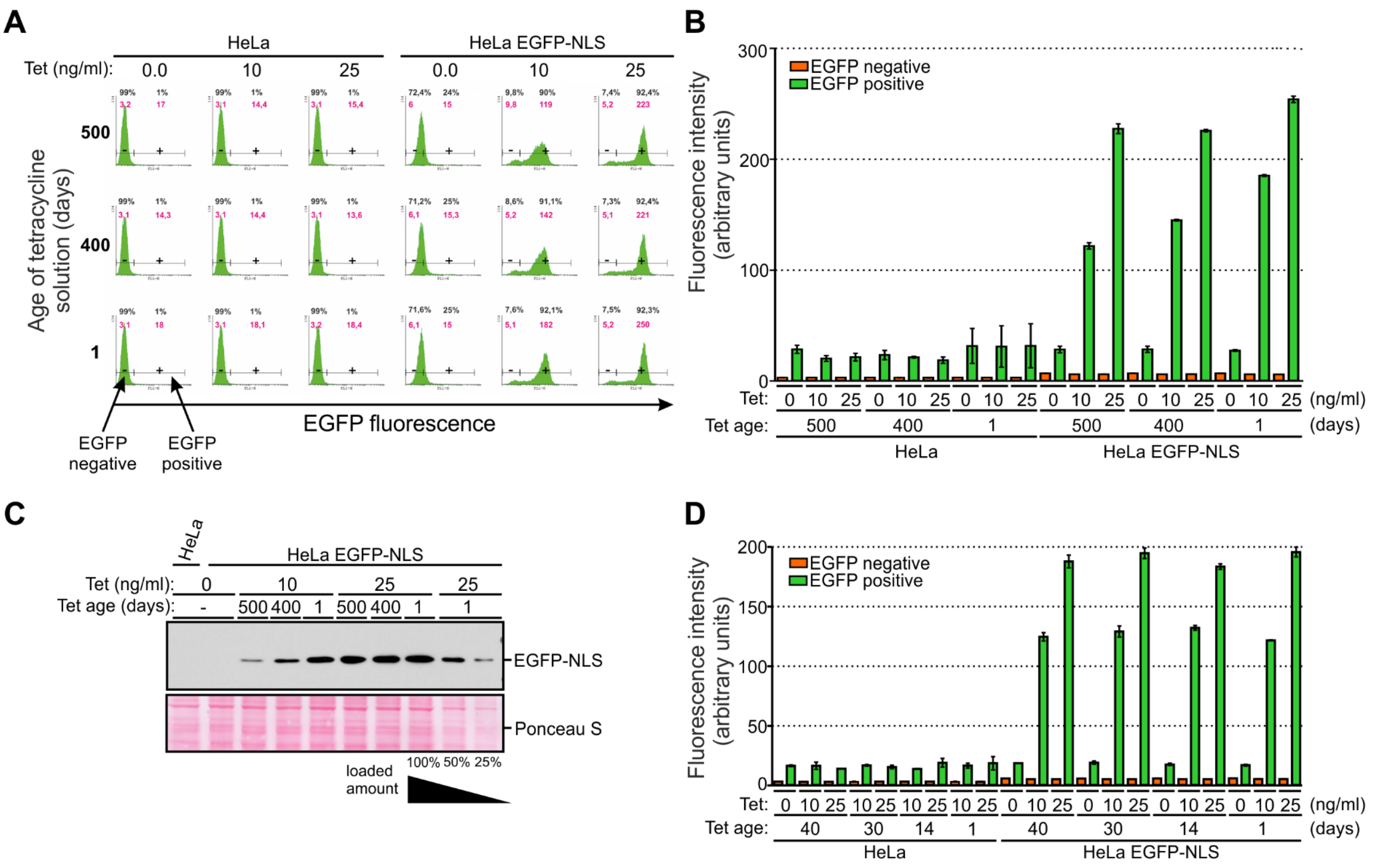

S3 Fig. Analysis of the stability of tetracycline solution. HeLa cells were stably transfected with a plasmid encoding nuclearly localized EGFP (EGFP-NLS) controlled by a Tet-responsive promoter. (A) Flow cytometry measurement of EGFP-NLS expression. Ethanol solutions of tetracycline were prepared 500, 400 or 1 day before use and stored at -20°C. (B) Quantitative representation of data shown in panel A. Error bars represent standard deviation. (C) Western blot analysis of EGFP-NLS level in samples analyzed in panel A and B. Different amounts of protein extracts from sample treated with 1 day old tetracycline (25 ng/ml) were loaded for comparison. Ponceau S staining of the membrane was performed as a loading control. (D) Analysis as in panel B but differently aged tetracycline solutions were examined.

The obtained results indicate that reproducible induction with tetracycline concentration lower than 25 ng/ml can be achieved with solutions as old as 40 days; otherwise differences in the level of transgenes induction can be expected – note differences in EGFP-NLS expression when 10 ng/ml tetracycline is used for induction (A, B, C).
