# Supplementary material for: Versatile approach for functional analysis of human proteins and efficient stable cell line generation using FLP-mediated recombination system
Source: PLoS One. 2018 Mar 28;13(3):e0194887. doi: 10.1371/journal.pone.0194887 (PMC5874048; doi:10.1371/journal.pone.0194887)
Supplement: S4 Fig — (PDF) [file pone.0194887.s004.pdf]

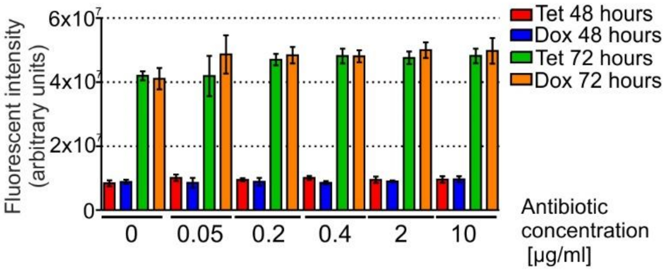


**S4 Fig. Viability test of 293 cells treated with tetracycline or doxycycline.** 293 cells were plated on a 96-well plate, treated with the indicated concentrations of inducers for the indicated period of time. The number of live cells was measured by the AlamarBlue fluorescence assay.
