# Supplementary material for: Versatile approach for functional analysis of human proteins and efficient stable cell line generation using FLP-mediated recombination system
Source: PLoS One. 2018 Mar 28;13(3):e0194887. doi: 10.1371/journal.pone.0194887 (PMC5874048; doi:10.1371/journal.pone.0194887)
Supplement: S5 Fig — (PDF) [file pone.0194887.s005.pdf]

1 miRNA cassette assembly by gene synthesis technology (external service).
Cassette is 486 bp long if three miRNAs are included.

2 Assembly of miRNA-insensitive CDS using highly efficient and reliable splice-PCR approach.
For coding sequences shorter than 1kb it is economically reasonable to consider gene synthesis (external service).

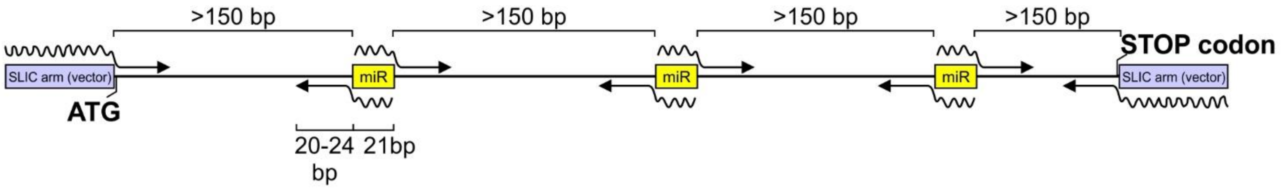

Scheme of splice-PCR if 3 different miRNAs are applied to silence the same gene

3 Cloning into vector.

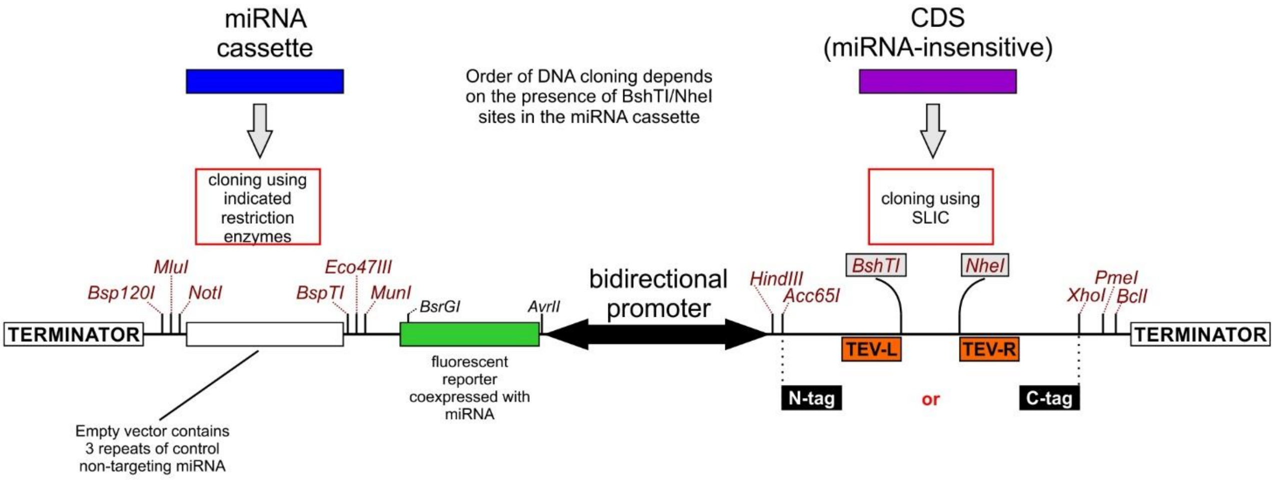

S5 Fig. Strategy of cloning into pKK-RNAi vectors.
