# Supplementary material for: Versatile approach for functional analysis of human proteins and efficient stable cell line generation using FLP-mediated recombination system
Source: PLoS One. 2018 Mar 28;13(3):e0194887. doi: 10.1371/journal.pone.0194887 (PMC5874048; doi:10.1371/journal.pone.0194887)
Supplement: S2 Supporting Information — (PDF) [file pone.0194887.s007.pdf]

# **S2 Supporting Information.** Detailed protocol for the SLIC procedure.

## **1.** Insert preparation by PCR

- Majority of inserts can be amplified using protocol below. If not, optimize your PCR.

*Reaction mixture:*

| | |
|---|---|
| $H_2O$ | 31.25 µl |
| Buffer for GC rich sequences (5x concentrated) | 10 µl |
| DMSO (100%) | 2.5 µl |
| Starter For (2.5 µM) | 2 µl |
| Starter Rev (2.5 µM) | 2 µl |
| dNTP (10 mM) | 1 µl |
| Template – cDNA or plasmid (10 ng of plasmid DNA/µl) | 1 µl |
| Phusion polymerase (2 U/µl, F530-L, Thermo Fisher Scientific) | 0.25 µl |

*Thermal cycles:*

| **temperature** | **time** | **cycling** |
|---|---|---|
| 98°C | 3 minutes | |
| 98°C | 10 seconds | 10 cycles<br>decrease (*) temperature by 1°C every cycle |
| 60°C * | 30 seconds | |
| 72°C | [adjust time to amplicon length] | |
| 98°C | 10 seconds | 25 cycles |
| 50°C | 30 seconds | |
| 72°C | [adjust time to amplicon length] | |
| 72°C | 7 minutes | |
| 10°C | indefinitely | |

- Primer overhangs for universal SLIC :

Forward: **GGATCC**gaaaacctgtacttccaagga**ACCGGT** *ATG (coding sequence)*
**BamHI** TEV-L **BshTI**

Reverse: **GATATC**accctgaaaatacaaattctc**GCTAGC** *coding sequence with (or without) termination codon*
**EcoRV** TEV-R **NheI**

- Purify PCR reaction with PCR clean-up kit (A&A Biotechnology, 021-50) or by agarose gel electrophoresis (Gel-out, A&A Biotechnology, 023-250). We use GelGreen dye (41005-1, Biotium) to stain DNA for gel-out procedure to avoid UV-mediated DNA damage.

## **2.** Vector preparation

- Digest DNA with restriction enzyme(s) and dephosphorylate. For universal SLIC approach described in paper use NheI and BshTI.

| | |
|---|---|
| DNA | 4 µg |
| Yellow Tango Buffer (10 x concentrated, Thermo Fisher Scientific) | 7 µl |
| NheI (10 U/µl, ER0972, Thermo Fisher Scientific) | 3.5 µl |
| BshTI (10 U/µl, ER1462, Thermo Fisher Scientific) | 2.5 µl |
| $H_2O$ | to 70 µl |

- Incubate 3 hours at 37°C, then add 1.5 µl of FastAP Thermosensitive Alkaline Phosphatase (1 U/µl, EF0651, Thermo Fisher Scientific) and continue incubation at 37°C for 30 minutes. Dephosphorylation of vector DNA reduces background in cloning (number of clones that harbor non-recombined vector) but is not mandatory.
- Inactivate enzymes by incubation at 75°C for 20 minutes.
- Run on agarose gel electrophoresis and purify from the gel (Gel-out, 023-250, A&A Biotechnology, elute DNA with water). We use GelGreen dye (41005-1, Biotium) and a blue light transilluminator to stain DNA in gel-out procedure to avoid UV-induced DNA damage.

*NOTE: We recommend using plasmid DNA purified on an anion exchange column (midi prep isolation).*

## **3.** SLIC reaction

*The procedure described below was adapted from Jeong et al., 2012 (PMID: 22610439).*

- prepare reaction on ice
- mix 100 ng of vector with insert at 1:4 molar ratio (vector : insert)
- add 1 µl of NEBuffer 2 (B7002S, NEB) and 1 µl of BSA (1 µg/µl, B9000S, NEB)
- add water to 10 µl
- add 0.5 µl of T4 DNA polymerase (3 U/µl; M0203L, NEB)
- incubate at room temperature for 2-5 minutes; DO NOT exceed 5 minutes
- put reaction on ice for ten minutes

## **4.** Transformation of chemocompetent bacteria

- thaw bacterial aliquot (100 µl) on ice
- add whole SLIC reaction, incubate on ice for 30 minutes
- incubate at 42°C for 90 seconds
- put on ice for 2 minutes
- add 400 µl of SOB medium
- incubate in Thermomixer R (Eppendorf) at 37°C, 900 rpm for 30 minutes
- plate to LB medium with 100 µg/ml ampicillin

*NOTE: We routinely use E. coli MH1 strain. Other strains suitable for DNA cloning (like DH5ά) can also be used. Remember to perform controls, e.g. SLIC reaction that contains pKK-RNAi vector without insert DNA.*

***LITERATURE***

*More information about SLIC cloning can be found in the following articles:*

Li MZ, Elledge SJ. Harnessing homologous recombination in vitro to generate recombinant DNA via SLIC. Nat Methods, 2007, 4(3):251-6 (PMID:17293868).

Jeong JY, Yim HS, Ryu JY, Lee HS, Lee JH, Seen DS, Kang SG. One-step sequence- and ligation-independent cloning as a rapid and versatile cloning method for functional genomics studies. Appl Environ Microbiol, 2012, 78(15):5440-3 (PMID: 22610439).
