# Supplementary material for: Versatile approach for functional analysis of human proteins and efficient stable cell line generation using FLP-mediated recombination system
Source: PLoS One. 2018 Mar 28;13(3):e0194887. doi: 10.1371/journal.pone.0194887 (PMC5874048; doi:10.1371/journal.pone.0194887)
Supplement: S3 Supporting Information — (PDF) [file pone.0194887.s008.pdf]

S3 Supporting Information. Detailed protocol for designing the miRNA cassette, splice-PCR and cloning into pKK-RNAi vectors.

General plan and order of DNA cloning:

1) Design miRNAs and decide on strategy of obtaining protein coding sequence (CDS) resistant to the miRNAs. We design miRNA with the approach described by Thermo Fisher Scientific.

2) Order miRNA cassette as an external service. The miRNA cassettes are difficult to construct by gene synthesis technology. Not all DNA synthesis companies are able to synthetize DNA which includes repeated sequences and hairpins. We order miRNA cassettes from BlueHeron company (http://blueheronbio.com) but other companies are also possible (e.g. GeneArt, ThermoFisher Scientific). Synthetized DNA is cloned in BlueHeron pUC-Kan Minus MCS vector. The vector lacks a multiple cloning site and its resistance marker (Kan^R) is different to that present in pKK-RNAi vectors (Amp^R), which simplifies subcloning. Time of delivery of synthetic DNA depends on its sequence. It usually takes between 2 and 4 weeks when standard procedure is applied. miRNA cassette is subcloned into one of pKK-RNAi vectors.

3) Create miRNA resistant CDS. Several methods for construction of CDS with silent mutations are possible (e.g. overlap PCR approach). If CDS is shorter than 1.0 kb it can be economically reasonable to order gene synthesis of the whole sequence. We describe splice-PCR which is simple and efficient.

4) Clone assembled or synthetic CDS into pKK-RNAi vector using SLIC or restriction enzyme-based cloning, respectively.

NOTE: In this protocol we assume that miRNA cassette is subcloned first and the CDS is cloned second. If the miRNA cassette contains BshTI or NheI restriction site(s), the order must be reversed as these enzymes are used in the universal SLIC-protocol. You can use other restriction enzymes for SLIC-cloning but the universal starters will not be compatible.

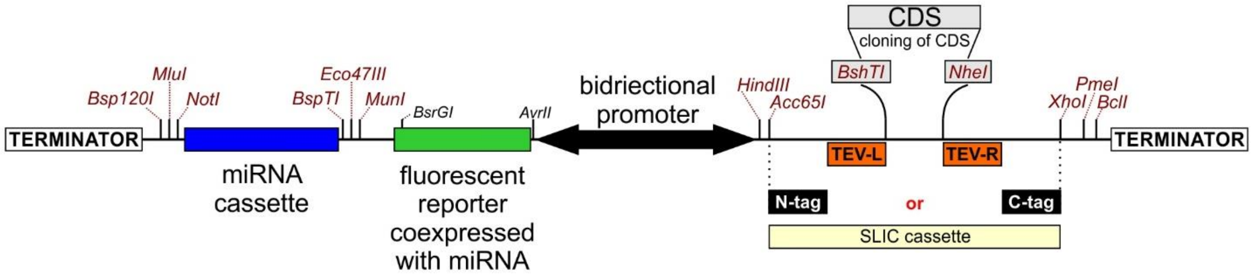

Scheme of a pKK-RNAi vector.

PROTOCOL

1. If you plan to rescue knockdown with a different version of the protein than wild-type, design the mutation first and note its nucleotide position. It will be helpful information when designing the miRNA cassette.

2. Check if CDS contains sites for enzymes belonging to group A and B (Tab. 1). It will determine what should be cloned into the vector first: the miRNA cassette or the CDS.

Tab. 1. Useful restriction enzymes (and their isoschizomers) that cut pKK-RNAi vectors

Group	Enzymes	Function
A	BspTI (AflII), Eco47III (AfeI), MunI (MfeI)	Cloning of miRNA cassette. Sites at the 5' end of the cassette.
B	NotI, MluI, Bsp120I (ApaI)	Cloning of miRNA cassette. Sites at the 3' end of the cassette.
C	BshTI, NheI	Cloning of CDS using universal SLIC. Other enzymes are also possible but are not compatible with universal SLIC approach.
D	AvrII (XmaJI), BspTI (AflII), Eco47III (AfeI), MunI (MfeI), BsrGI*	Exchange or deletion of miRNA expression reporter.<br>*NOTE: BsrGI can be used if it is necessary to exchange the reporter without removing the NLS, however, the sequence recognized by BsrGI is also present between TEV-L and TEV-R sequences (all vectors) and in some tags (vectors encoding EGFP or mCherry as a tag)
E	HindIII/Acc65I XhoI/PmeI/BclI,	Removal of the SLIC cassette, including tag.<br>This enables exchange of this fragment between plasmids.
F	EcoRV with HindIII/Acc65I/BamHI	Removal of the SLIC cassette, leaving the C-terminal tag.<br>This enables exchange of this fragment between plasmids.
G	BamHI with XhoI/PmeI/BclI	Removal of the SLIC cassette, leaving the N-terminal tag.<br>This enables exchange of this fragment between plasmids

3. Design of miRNA:

- Launch BLOCK-iT™ RNAi Designer from Thermo Fisher Scientific available at the following website: http://rnaidesigner.lifetechnologies.com/rnaiexpress/setOption.do?designOption=mirna
- Paste CDS sequence.
- Set following options: ORF, BLAST – Human – Homo sapiens, Minimum GC – 35%, Maximum GC – 55%.
- Click „RNAi design"

NOTE: If CDS contains many sequence repeats it can be impossible to design miRNA. If so, include untranslated regions for miRNA design. If miRNA targets sequence present in UTR, silent mutations do not need to be introduced into cloned CDS.

4. Choosing miRNA:

- miRNA ranked with at least 4 out of 5 stars are eligible. In most cases there are several 5-star miRNAs to choose from. So far we have not had to use miRNAs that ranked 3 stars or lower. Here, we describe a protocol that involves combination of three different miRNAs into one miRNA cassette. A smaller number of miRNAs can also be used, however, we have only ever used three.
- If possible we keep the distance between miRNA targeted sequences at least 150 nt. Even distribution of miRNA targeted sequences can be beneficial for splice-PCR but is not obligatory (see step 5.7).
- miRNA should not target region where catalytic (or any other) mutation is planned (see step 5.1). Otherwise, it should be taken into account during assembly of miRNA-insensitive CDS.
- miRNA should not contain all restriction sites for enzymes from groups A and B (Tab. 1). Otherwise it will be difficult to subclone the miRNA cassette into the vector. If the miRNA is cloned first and the CDS is to be cloned by universal SLIC, the miRNA must not contain BshTI and NheI restriction sites as these enzymes are used for vector linearization before CDS cloning (see step 5.2).

5. Design of miRNA cassette:

- Select chosen miRNAs and click „Design miR RNAi":

Select	No.	Start	Sequence(DNA)
☐	1	129	CGCAAGTACCCGTCCATCATA
☐	2	995	GTTTATCTTCCTTCGGCTTAA
☐	3	1235	AAGTGGTTATGTCAATCTGCA
☐	4	1443	GGTTCTCAAGTAGCCAGTAAT
☐	5	1609	CAGAGGATAATGTCAGGTTAT
☐	6	1668	TTTGATGTGGATGCAGCTGAT
☐	7	1685	TGATGAGAAATTCCGTCGGAA
☐	8	1947	ATGAGTGATCCTGATTCTAGT
☐	9	2623	GAAACTTATACAGGCCGCTTT
☐	10	2920	CACCCTCAGGAAGATACAATT

Design miR RNAi

- Sequences of miRNAs appear that include sequences incompatible with pKK-RNAi vectors (TGCTG, CCTG and C). Select and copy "Top Strand" downstream of the sequence TGCTG (see box sequence in figure below). Substitute "Neg miR" sequence (AAATGTACTGCGCGTGGAGACGTTTTGGCCACTGACTGACGTCTCCACGCAGTACATTT) in the pKK-RNAi vector with the sequence of the designed miRNA. Repeat for other miRNAs. Sequences of miRNAs contain a loop-forming sequence from murine miR-155 (GTTTTGGCCACTGACTGAC).

Sequence to be selected and copied

Select	No.	Start	Oligo Type	Oligo Sequence
☑	1	995	Top Strand	5'- TGCTGTTAAGCCGAAGGAAGATAAACGTTTTGGCCACTGACTGACGTTTATCTCTTCGGCTTAA -3'
			Bottom Strand	5'- CCTGTTAAGCCGAAGAGATAAACGTCAGTCAGTGGCCAAAACGTTTATCTTCCTTCGGCTTAAC -3'
			ds Oligo	5'- TGCTGTTAAGCCGAAGGAAGATAAACGTTTTGGCCACTGACTGACGTTTATCTCTTCGGCTTAA -3'<br>3'- CAATTCGGCTTCCTTCTATTTGCAAAACCGGTGACTGACTGCAAATAGAGAAGCCGAATTGTCC -5'

- After *in silico* assembly of the miRNA cassette check for presence of restriction sites for the enzymes that will be used for cloning. We use Bsp120I and MunI as the enzymes of first choice for subcloning the miRNA cassette.

NOTE: In our experience the cloning efficiency is markedly lower when ApaI (isoschizomer of Bsp120I) is used. We noticed that ApaI "sticks" to DNA during thermal inactivation.

- Check for presence of BshTI and NheI sites. If the miRNA cassette contains these sites the CDS must be cloned first.
- Select the sequence of miRNA cassette, copy and paste to some text editor software (like Microsoft Notepad) that will help you to prepare sequences for ordering. By default we span the miRNA cassette from 3 nucleotides upstream of the MunI site through 3 nucleotides downstream of the Bsp120I site (see picture below). This allows changing the cloning strategy if necessary. If the cassette includes 3 miRNAs it is 486 nucleotides long.

```
             MunI   Eco47III    BspTI                              NotI     MluI                          Bsp120I
Start (5') GTGCAATTGGAGCGCTAGTCTTAAGTGATCC..........AGATCTGCGGCCGCACGCGTCATATCTAGTGATCTAGAGGGCCCGCG (3') End
           CACGTTAACCTCGCGATCAGAATTCACTAGG..........TCTAGACGCCGGCGTGCGCAGTATAGATCACTAGATCTCCCGGGCGC
```

6. Ordering the miRNA cassette

So far we have been ordering synthesis of miRNA cassettes from BlueHeron (http://blueheronbio.com) but other vendors are also possible (e.g. GeneArt, ThermoFisher Scientific). Delivery time of synthetic DNA depends on its sequence. It usually takes 2 to 4 weeks when the standard procedure is applied. In the protocol we assume that synthetic DNA was delivered cloned into a vector that harbors a resistance marker different than the pKK-RNAi vectors and the backbone of the vector does not have restriction sites for enzymes that will be used for the cassette subcloning (*e.g.* pUC-Kan Minus MCS vector from BlueHeron). These features made subcloning easier but are not mandatory.

7. Subcloning the miRNA cassette

Our standard conditions are described. Because the plasmid that harbors the cassette has a different selection marker to the pKK-RNAi vector it is not necessary to gel-out the insert after restriction digestion. If so, it is prudent to replicate colonies obtained after ligation on a selective plate appropriate for the donor plasmid to ensure that desired recombinant molecule has been selected for further steps of the procedure.

DNA digestion:

DNA (pKK-RNAi vector or donor plasmid with miRNA)	– 2 µg
Blue Buffer (Thermo Fisher Scientific, 10x concentrated)	– 4 µl
Bsp120I (10 U/µl, ER0131, Thermo Fisher Scientific)	– 1 µl
MunI (10 U/µl, ER0751, Thermo Fisher Scientific)	– 1 µl
H_2O	– to 40 µl

Final DNA concentration: 50 ng/µl

- Incubate 3h at 37°C. 30 minutes before termination of the reaction add 1.5 µl phosphatase (1 U/µl, FastAP, EF0651, Thermo Fisher Scientific) to sample with pKK-RNAi vector and continue the incubation.
- Inactivation enzymes at 80°C for 20 minutes.
- It is not necessary to clean-up or gel-out DNA.

DNA ligation and bacterial transformation:

Digestion mixture of pKK-RNAi (50 ng/µl)	– 2 µl
Digestion mixture of miRNA donor plasmid (50 ng/µl)	– 3 µl
T4 DNA ligase buffer (Thermo Fisher Scientific, 10x)	– 2 µl
T4 DNA ligase (5 U/µl, EL0011, Thermo Fisher Scientific)	– 1.5 µl
H_2O	– 11.5 µl

- Incubate at least 1h at room temperature (we routinely incubate overnight, but 1h reaction also gives satisfactory results).
- Transform bacteria with 7 µl of the reaction. Routinely we use E. coli MH1 strain. Other strains suitable for DNA cloning (like DH5alfa) can also be used. Select on medium with ampicillin (100 µg/ml).
- Remember to perform controls, e.g. ligation reaction that contains pKK-RNAi vector without insert DNA.

8. Primer design for splice-PCR:

- Mark miRNA targeted sequences in CDS.
- Introduce silent mutations within miRNA targeted sequences. We change at least 7 nucleotides. Avoid rare codons. You may find the C.U.R.R.F software helpful in designing the mutations (PMID:22161280). After uploading human codon usage information (available *e.g.* from http://www.kazusa.or.jp/codon/) the software displays the frequency of a given codon. The software used to be available at https://tu-dresden.de/die_tu_dresden/fakultaeten /fakultaet_mathematik_und_naturwissenschaften/ fachrichtung_biologie/mikrobiologie/allgemeine_mikrobiologie/currf
- Using the modified CDS, create primer sequences. They should include 21 nucleotides of the miRNA targeted sequence as overhang and cover 20-24 adjacent nucleotides (see picture below).

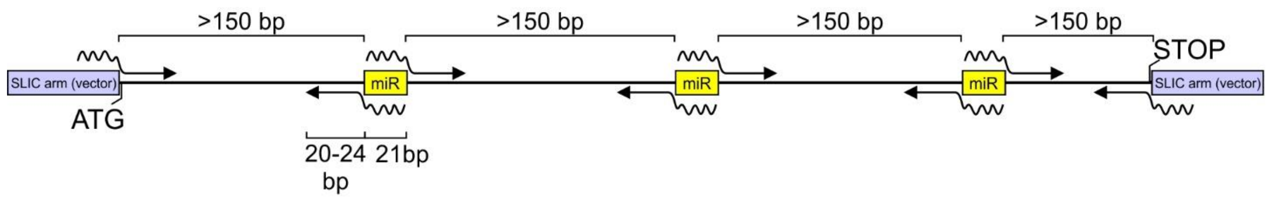

- The outer starters must have overhangs that are complementary to pKK-RNAi vectors. The following overhangs are used with universal SLIC:

Forward:
GGATCCgaaaacctgtacttccaagga**ACCGGT** *ATG (coding sequence)*
BamHI TEV-L **BshTI**

Reverse:
GATATCaccctgaaaatacaaattctc**GCTAGC** *coding sequence with (or without) termination codon*
EcoRV TEV-R **NheI**

9. Assembly of CDS using splice-PCR

- Amplify fragments of CDS (4 fragments if 3 miRNAs are used). Our standard conditions are as follows:

Reaction mixture:

H_2O	– 31.25 µl
Buffer for GC rich sequences (5x concentrated)	– 10 µl
DMSO (100%)	– 2.5 µl
Starter For (2.5 µM)	– 2 µl
Starter Rev (2.5 µM)	– 2 µl
dNTP (10 mM)	– 1 µl
template (10 ng of plasmid DNA/µl)	– 1 µl
Phusion polymerase (2 U/µl, F530-L, Thermo Fisher Scientific)	– 0.25 µl

PCR protocol (takes about 1.5 h):

98°C – 3 min
98°C – 10 s
60°C→50°C – 30 s
72°C – 1 min
(10 cycles, decrease of 1°C per cycle)
98°C – 10 s
50°C – 30 s
72°C – 1 min
(25 cycles)
72°C – 7 min
10°C – ∞

You may need to extend time of elongation if a product longer than 3 kbs is to be amplified.

- Run agarose gel electrophoresis and gel-out the product. We use the „Gel-out" kit from A&A Biotechnology (023-250, elution with 35 µl H_2O). We use GelGreen dye (41005-1, Biotium) to stain DNA and visualize it with a blue light transilluminator.

- Run first round of splice-PCR:

Reaction mixture:

H_2O	– 37 µl
DNA fragment "1" (~10-20 ng/µl)	– 2 µl
DNA fragment "2" (~10-20 ng/µl)	– 2 µl
Buffer with $MgSO_4$ (10 x concentrated)	– 5 µl
dNTP (2 mM)	– 3 µl
Pfu polymerase (2.5 U/µl, EP0501, Thermo Fisher Scientific)	– 1 µl

If you are unsuccessful with Pfu polymerase, try using Phusion.

PCR protocol:

95°C – 3 min
95°C – 30 s
58°C – 30 s
72°C – 4 min
(4 cycles)
72°C – 7 min
10°C – pause

After cooling down stop thermocycler program, add 5 µl (2.5 µM) of each starter and continue the program:

95°C – 10 s
95°C – 30 s
58°C – 1 min
72°C – 3 min
(25 cycles)
72°C – 7 min
10°C – ∞

You may need to extend time of elongation if a product is longer than 2.5 kb is to be amplified.

- Run agarose electrophoresis and gel-out the product. We use „Gel-out" A&A Biotechnology (023-250, elution with 35 µl H_2O). We use GelGreen dye is used to stain DNA and visualize it with a blue light transilluminator.
- Run second round of splice-PCR. The same reaction conditions as in the first round of splice-PCR are applied. Alternatively, you can perform a SLIC reaction that joins 3 DNA molecules (two PCR products and the vector).

10. Cloning of CDS using SLIC

Vector preparation:

- Digest DNA with restriction enzyme(s) and dephosphorylate. For the universal SLIC approach described in paper use NheI and BshTI

DNA – 4 µg
Yellow Tango Buffer (10 x concentrated, Thermo Fisher Scientific) – 7 µl
NheI (10 U/µl, ER0972, Thermo Fisher Scientific) – 3,5 µl
BshTI (10 U/µl, ER1462, Thermo Fisher Scientific) – 2,5 µl
H_2O – add water to 70 µl

NOTE: We recommend using plasmid DNA purified on an anion exchange column (midi prep isolation)

- Incubate 3 hours at 37°C then add 1.5 µl of FastAP Thermosensitive Alkaline Phosphatase (1 U/µl, EF0651, Thermo Fisher Scientific) and continue incubation at 37°C for 30 minutes. Dephosphorylation of vector DNA reduces background in cloning (number of clones that harbors non-recombinereed vector) but is not mandatory.
- Inactivate enzymes by incubation at 75°C for 20 minutes

- Resolve by agarose gel electrophoresis and purify from the gel (Gel-out, 023-250, A&A Biotechnology, elute DNA with water). We use GelGreen dye (41005-1, Biotium) to stain DNA in gel-out procedure and visualize it with a blue light transilluminator to avoid UV-induced DNA damage.

<u>SLIC reaction</u>

- Prepare reaction on ice
- Mix 100 ng of vector with insert at 1:4 molar ratio (vector:insert)
- Add 1 µl of NEBuffer 2 (B7002S, NEB) and 1 µl of BSA (1 µg/µl, B9000S, NEB)
- Add water to 10 µl
- Add 0.5 µl of T4 DNA polymerase (3 U/µl; M0203L, NEB)
- Incubate at room temperature for 2-5 minutes; DO NOT exceed 5 minutes
- Put reaction on ice for ten minutes

<u>Transformation of chemocompetent bacteria</u>

- Thaw bacterial aliquot (100 µl) on ice
- Add whole SLIC reaction, incubate on ice for 30 minutes
- Incubate at 42°C for 90 seconds
- Put on ice for 2 minutes
- Add 400 µl of SOB medium
- Incubate in Thermomixer R (Eppendorf) at 37°C, 900 rpm for 30 minutes
- Plate to LB medium with 100 µg/ml ampicillin

NOTE: We routinely use E. coli MH1 strain. Other strains suitable for DNA cloning (like DH5ά) can also be used. Remember to perform controls, e.g. SLIC reaction that contains pKK-RNAi vector without insert DNA

11. Starters for Sanger sequencing:

Sequencing of miRNA cassette			
**Name**	**Sequence**	**Tag**	**Description**
RSZ839	AACGACCCAACACCCGTGCG	Any	Anneals to TK poly(A) signal. Sufficient to sequence the cassette that is 486 bp in length.

Sequencing of CDS			
RSZ840	CAATGCGATGCAATTTCCTC	FLAG at the C-terminus (or other short tag), any tag present at the N-terminus	Anneals to BGH poly(A) signal. Sequencing reaction results in sequence of 70 bp vector backbone, sequence of tag (if present), 20 bp including TEV-R and the 3' end of CDS.
RSZ841	CTTGCCGGTGGTGCAGA	EGFP at the C-terminus	Sequencing reaction results in sequence of about 70 bp of EGFP, 20 bp including TEV-R and the 3' end of CDS.
RSZ842	TCGCCGACCACTACCAGC	EGFP at the N-terminus	Sequencing reaction results in sequence of about 70 bp of EGFP, 20 bp including TEV-L and the 5' end of CDS.
RSZ843	GGCCACCCTTGGTCACCT	mCherry at the C-terminus	Sequencing reaction results in sequence of about 70 bp of mCherry, 20 bp including TEV-R and the 3' end of CDS.
RSZ844	ACTACGACGCTGAGGTCA	mCherry at the N-terminus	Sequencing reaction results in sequence of about 80 bp of mCherry, 20 bp including TEV-L and the 5' end of CDS.
