# Supplementary material for: Versatile approach for functional analysis of human proteins and efficient stable cell line generation using FLP-mediated recombination system
Source: PLoS One. 2018 Mar 28;13(3):e0194887. doi: 10.1371/journal.pone.0194887 (PMC5874048; doi:10.1371/journal.pone.0194887)
Supplement: S5 Supporting Information — (PDF) [file pone.0194887.s010.pdf]

S5 Supporting Information. Detailed protocol for stable cell line generation.

Stable Cell Line Generation – Day by Day Protocol

General guidelines

Healthy parental cells are of utmost importance. Poor cell health can impede stable line generation or be carried over to derivatives. Use fresh cells, no more than 10 passages from unfreezing (8-15 days after unfreezing). Do not plate cells from an overly dense culture, maintain a maximum confluence of 90%. Cells plated for transfection should be replated a day or (optimally) two days before. Do not use cells replated three or more days before.

Cells can be cultured in medium supplemented with standard antibiotics (penicillin/streptomycin), however, it is not advised. On the one hand, omitting antibacterial agents runs the risk of culture contamination but on the other hand you will know immediately whether your work is sterile. Maintaining a stable cell line with a latent contamination is a poor idea as an antibiotic-sensitive application may present itself in the future, for which the cells line will be useless.

At our laboratory we do not use antibacterial/antifungal agents in routine culture and only add penicillin/streptomycin at later stages of expanding large-scale cultures, *e.g.* ten to twenty 145 mm dishes for protein purification, when cells are soon to be harvested and a contamination can be quite costly.

The Flp-In/FRT locus and the TetR gene present in Thermo Fisher Scientific Flp-In System cell lines are maintained by zeocin and blasticidin S selection, respectively. You may want to add these selection agents to the medium for culture of parental cells. Note, that after FRT-directed plasmid integration cells become sensitive to zeocin. They are still resistant to blasticidin S and become insensitive to hygromycin B.

Blasticidin S is thermolabile and/or sensitive to freeze-thaw cycles. We make small aliquots and use it 1-2 times.

Hygromycin B from different suppliers can vary substantially. We have used Hygromycin B from Invitrogen (currently Thermo Fisher Scientific, 10687010) and Hygromycin B Gold from Invivogen (ant-hg). We have found that they differ in purity and activity. Therefore, we recommend that you test your parental cell line for sensitivity. pOG44, the plasmid encoding the FLP recombinase, is crucial to generate Flp-In cell lines. We have seen this plasmid undergo spontaneous recombination in *E. coli*. If you transfect your parental cell line with different FLP-competent plasmids and get no colonies, most likely your pOG44 is to blame. We strongly suggest you make one large-scale preparation, check the restriction pattern well, and aliquot it.

NOTE: We use 293 cells from Thermo Fisher Scientific (Flp-In™ 293 T-REx) and HeLa cells described by Castello et al. (PMID: 22658674). There are several other Flp-In competent derivatives of the original 293 cells described by Graham and Smiley in 1977 (PMID: 886304). Other HeLa derivatives were described as well. If you use parental 293 or HeLa Flp-In cells different to ours, it can be necessary to modify some conditions, like number of cells plated.

PROTOCOL

Day -2: cell plating

Plate 500,000 (293) or 400,000 (HeLa) cells in 2 ml of medium to a 6-well plate.
Optimally they should be 50-60% confluent the following day.

Day -1: cell transfection

Prepare transfection complexes by adding 1.0 µg of pOG44, 0.3 µg of targeting construct in 250 µl of OptiMEM (51985-026, Thermo Fisher Scientific). Add 2 µl of TransIT 2020 transfection reagent (Mirus, MIR5400). Mix by tapping tube with finger. Incubate at room temperature for 20 minutes and add whole mixture to cells.

Two controls can be set up:

1) <u>transfection efficiency:</u> cells transfected with 1.3 µg of DNA construct encoding a fluorescent reporter (like pKK-TEV-EGFP, pEGFP-N1 from Clontech, etc.)
   Inspect the culture on a fluorescence microscope or analyse with flow cytometry on the next day (cells should be induced upon transfection, if applicable). We usually get transfection efficiencies of around 70% (293) or 40-50% (HeLa), which translates to a satisfactory efficiency of stable cell line generation.
2) <u>selection control:</u> non-transfected cells cultured in selection medium
   Expose this culture to the selection medium and observe loss of viability and cell death. At the beginning of the selection process untransfected cells may appear far better than the transfected ones. This survival advantage is probably due to the lack of transfection related stress. Eventually, though, the untransfected cells will die whereas the transfected ones will grow.

Day 0: begin selection

a. **Day 0** – Replate cells to 5 ml of medium in 60 mm dish. Add selection antibiotics:
   293 cells: 50 µg/ml hygromycin B (Invitrogen) and 10 µg/ml blasticidin S (Invivogen);
   HeLa cells: 175 µg/ml hygromycin B (Invitrogen) and 10 µg/ml blasticidin S (Invivogen).

NOTE: The death will be quite prolonged: its early sign, gradual detachment from the dish and taking on an oval shape, will become noticeable on day 1 but some cells carry on through day 3. Figure below shows selection process.

b. **Day 3** – replace selection medium.
c. **Day 6** – replace medium of 293 and most probably replate HeLa to the same dish (if HeLa cultures are of >40% confluency or dense colonies are observed cells should be replated).

   <u>Replating to the same dish:</u> remove medium, rinse with 3 ml of PBS, drop 2-4 drops of trypsin, trying to target colonies, incubate for 3 minutes at 37°C, add 1 ml of selection medium and resuspend with an automatic pipette. Add 4 ml of selection medium, disperse by rocking dish and place in incubator.

NOTE: Expect a new wave of cell death due to breaking up dense cells as selection works poorly at high cell densities.

d. **Day 7** – if HeLa were replated, most likely it is necessary to replace medium as there will be a lot of dead cells floating (alternatively you can postpone to **Day 8**).
e. **Day 9** – add 1-1.5 ml of selection medium to 293. If many dead cells floating in HeLa, replace medium. Well attached cells should appear by this point, perhaps even colonies (especially in HeLa).
f. **Day 13** – if there are many dead cells in HeLa, replace medium. Colonies should be visible by now but in extreme cases may appear as late as **Day 20**, especially for 293.
g. **Day 14-20** – wait for HeLa to grow to at least 60-70% confluence and then replate to 100 mm dish. If there were few colonies, then by the time they reach 60-70% confluence the cells in the middle of colonies will begin dying, in which case replate cells to a 60 mm dish, and later to a 100 mm dish.
h. **Day 14-20** – observe 293 and wait for them to grow to large colonies but not until they begin dying in the middle. If there are more than 2 colonies, replate cells to the same 60 mm dish and continue expanding the cell culture similarly as for HeLa. If you only get 1-2 original colonies, replate them to a smaller dish, e.g. a 6-well plate and then increase culture scale as cells multiply.

Having expanded the culture, freeze your cells in aliquots, 3-4 per confluent 100 mm dish. DO NOT terminate your original culture at this point! Should anything go wrong with freezing, you will lose your line it took you a month to

establish. Always continue the culture until you verify your frozen cells can be unfrozen and cultured for a few days in selection medium.

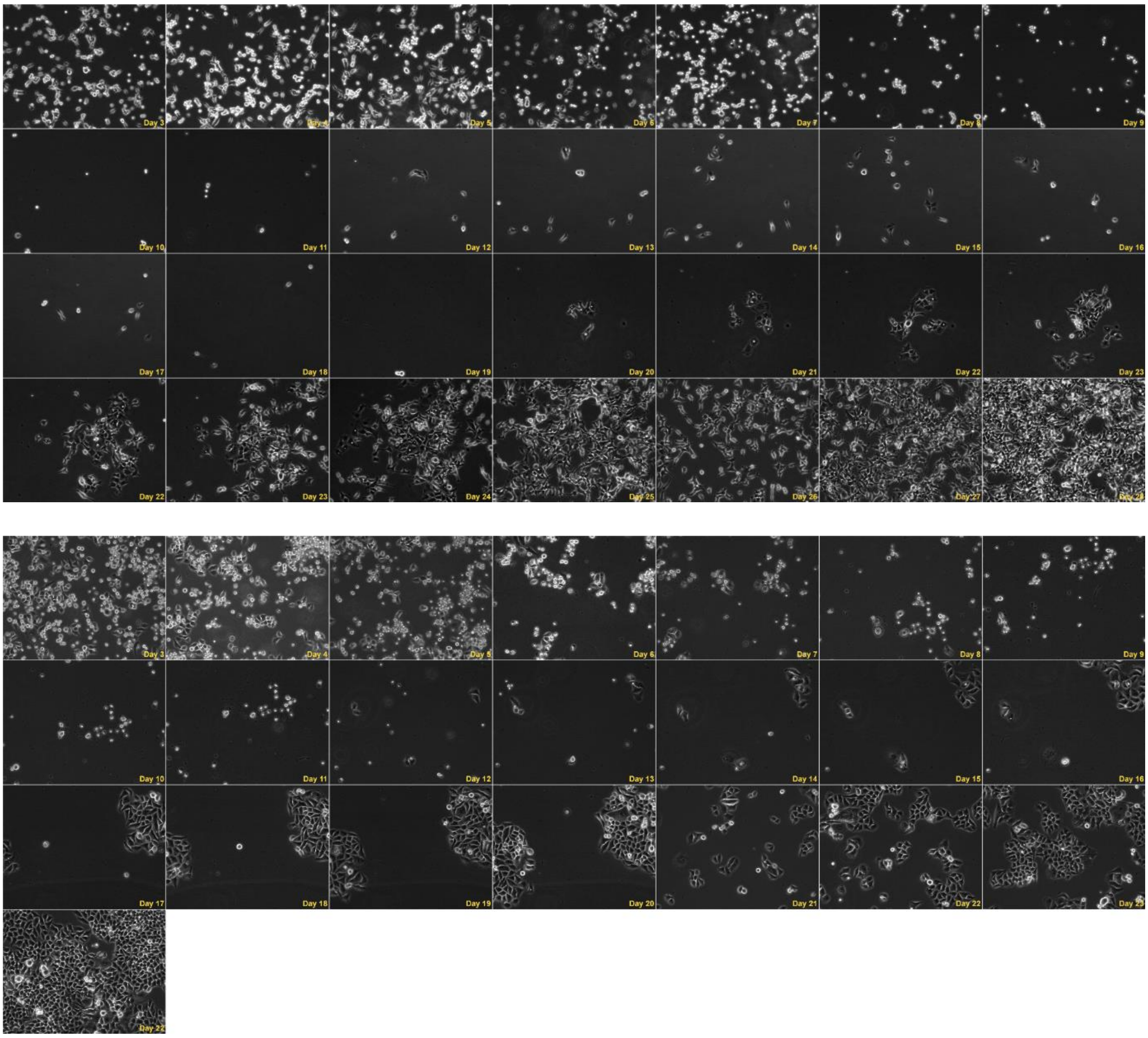

Figure 1. Day by day photos of the selection process. 293 (upper panel) and HeLa (lower panel). First photo was taken on **Day3**. Cells were replated on Days 25 and 28 (293) and Days 20 and 22 (HeLa).
