# Supplementary material for: Versatile approach for functional analysis of human proteins and efficient stable cell line generation using FLP-mediated recombination system
Source: PLoS One. 2018 Mar 28;13(3):e0194887. doi: 10.1371/journal.pone.0194887 (PMC5874048; doi:10.1371/journal.pone.0194887)
Supplement: S6 Supporting Information — (PDF) [file pone.0194887.s011.pdf]

S6 Supporting Information. Maps of all reported vectors.

series pKK

Created with SnapGene®

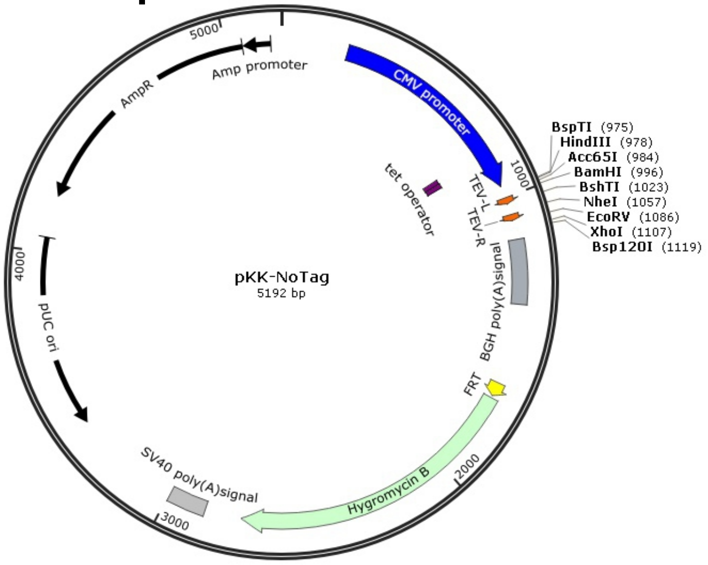

Created with SnapGene®

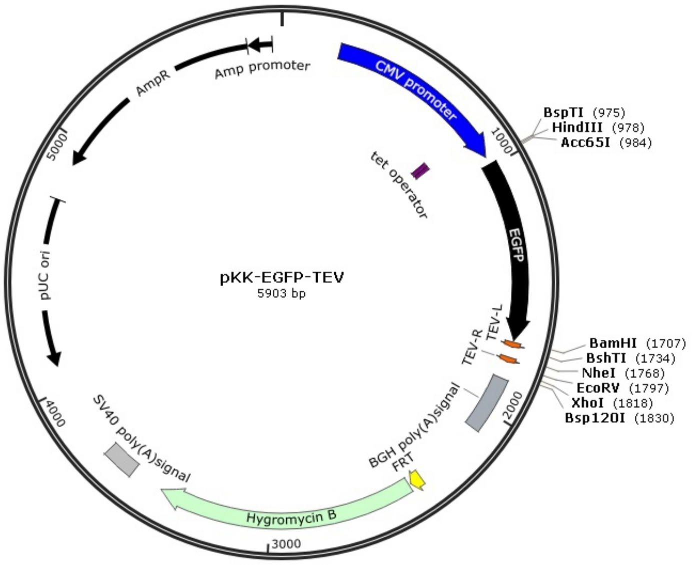

Created with SnapGene®

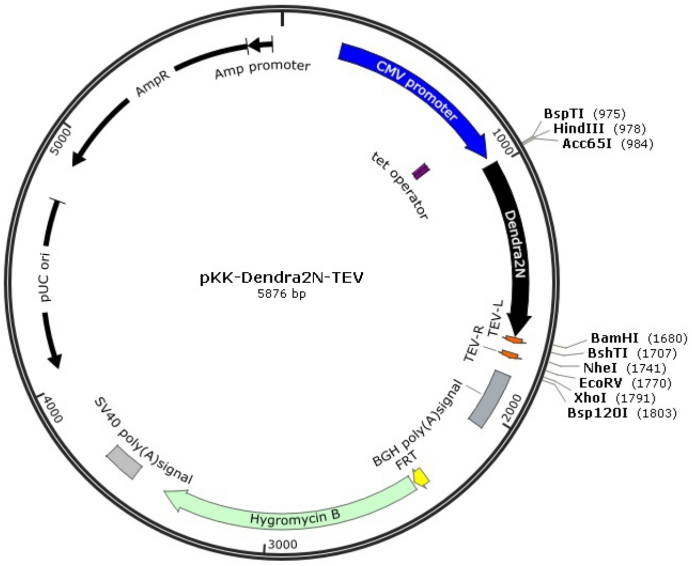

Created with SnapGene®

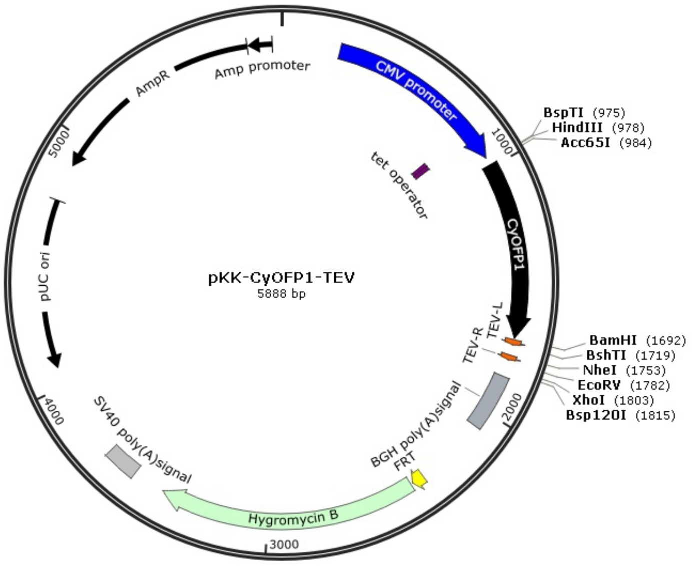

Created with SnapGene®

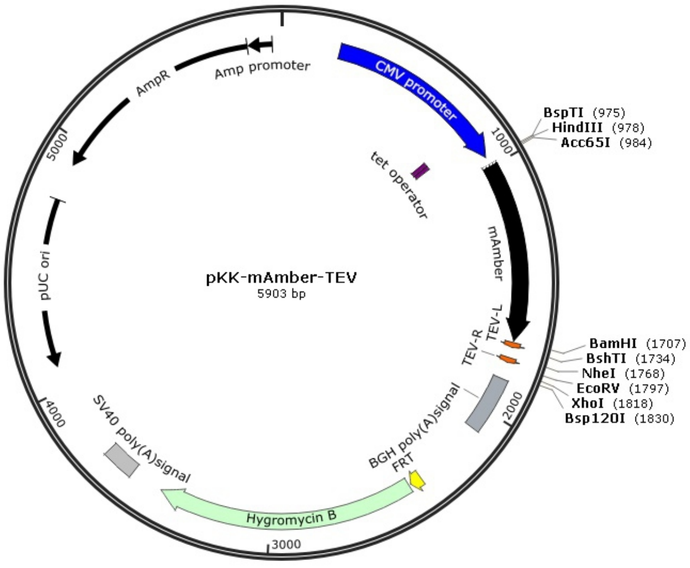

Created with SnapGene®

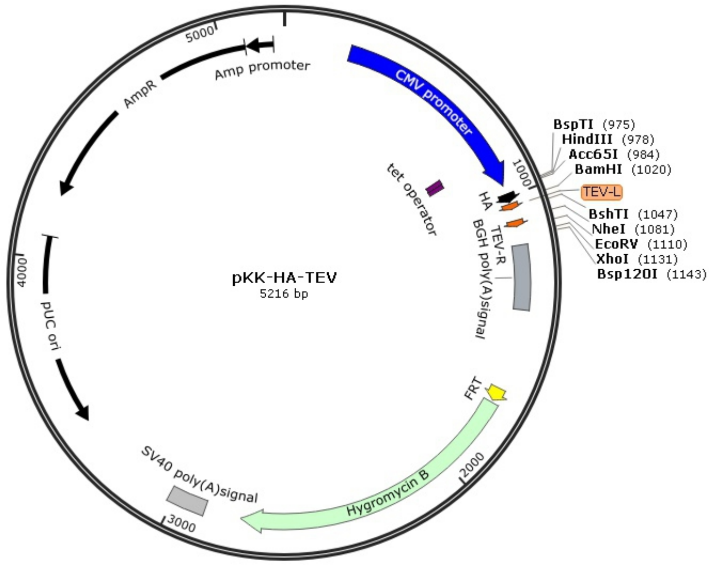

series pKK

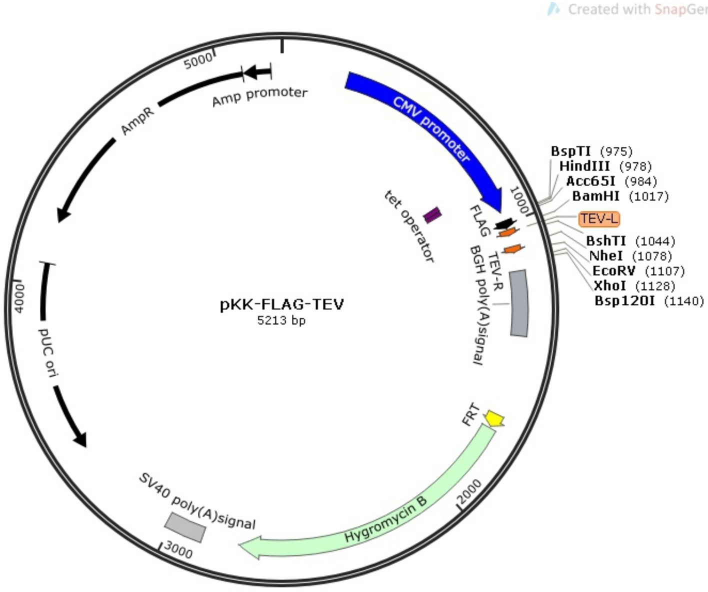
Created with SnapGene®
pKK-FLAG-TEV
5213 bp
CMV promoter
Amp promoter
AmpR
pUC ori
SV40 poly(A)signal
Hygromycin B
FRT
BGH poly(A)signal
TEV-R
TEV-L
FLAG
tet operator
BspTI (975)
HindIII (978)
Acc65I (984)
BamHI (1017)
BshTI (1044)
NheI (1078)
EcoRV (1107)
XhoI (1128)
Bsp120I (1140)
1000
2000
3000
4000
5000

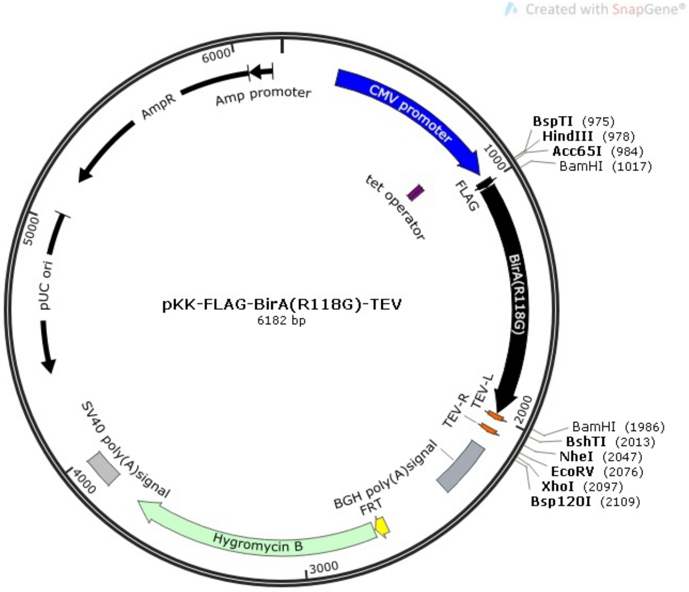
Created with SnapGene®
pKK-FLAG-BirA(R118G)-TEV
6182 bp
CMV promoter
Amp promoter
AmpR
pUC ori
FLAG
BirA(R118G)
tet operator
TEV-R
TEV-L
BGH poly(A)signal
FRT
Hygromycin B
SV40 poly(A)signal
BspTI (975)
HindIII (978)
Acc65I (984)
BamHI (1017)
BamHI (1986)
BshTI (2013)
NheI (2047)
EcoRV (2076)
XhoI (2097)
Bsp120I (2109)
1000
2000
3000
4000
6000

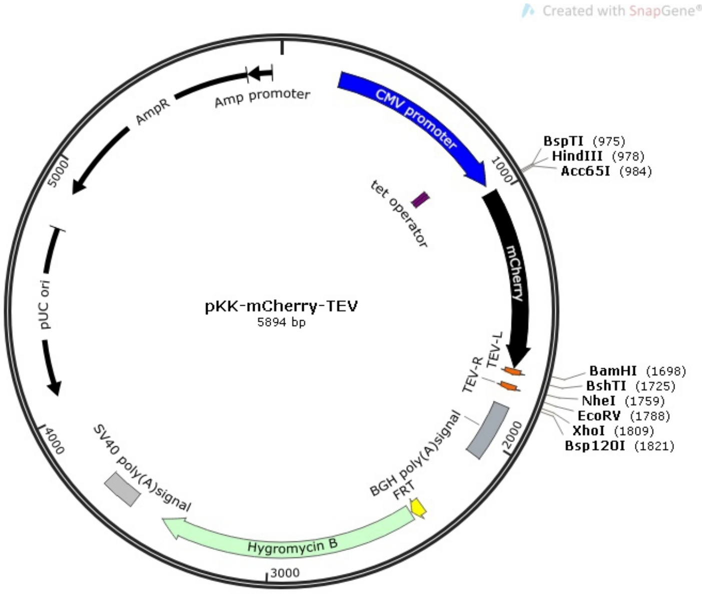
Created with SnapGene®
pKK-mCherry-TEV
5894 bp
CMV promoter
Amp promoter
AmpR
pUC ori
tet operator
mCherry
TEV-L
TEV-R
BGH poly(A)signal
FRT
Hygromycin B
SV40 poly(A)signal
BspTI (975)
HindIII (978)
Acc65I (984)
BamHI (1698)
BshTI (1725)
NheI (1759)
EcoRV (1788)
XhoI (1809)
Bsp120I (1821)
1000
2000
3000
4000
5000

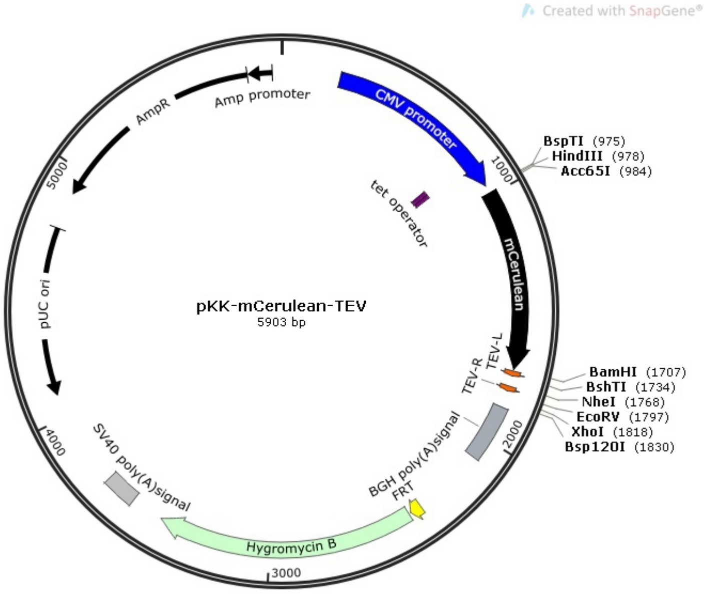
Created with SnapGene®
pKK-mCerulean-TEV
5903 bp
CMV promoter
Amp promoter
AmpR
pUC ori
tet operator
mCerulean
TEV-L
TEV-R
BGH poly(A)signal
FRT
Hygromycin B
SV40 poly(A)signal
BspTI (975)
HindIII (978)
Acc65I (984)
BamHI (1707)
BshTI (1734)
NheI (1768)
EcoRV (1797)
XhoI (1818)
Bsp120I (1830)
1000
2000
3000
4000
5000

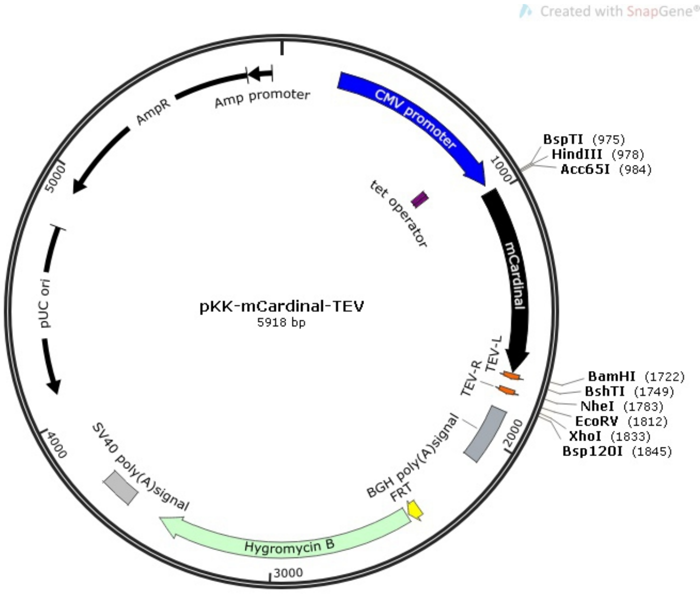
Created with SnapGene®
pKK-mCardinal-TEV
5918 bp
CMV promoter
Amp promoter
AmpR
pUC ori
tet operator
mCardinal
TEV-L
TEV-R
BGH poly(A)signal
FRT
Hygromycin B
SV40 poly(A)signal
BspTI (975)
HindIII (978)
Acc65I (984)
BamHI (1722)
BshTI (1749)
NheI (1783)
EcoRV (1812)
XhoI (1833)
Bsp120I (1845)
1000
2000
3000
4000
5000

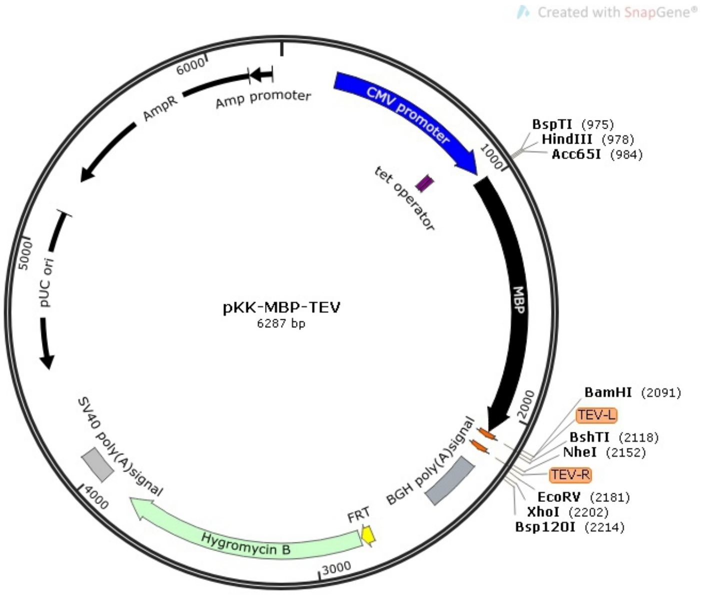
Created with SnapGene®
pKK-MBP-TEV
6287 bp
CMV promoter
Amp promoter
AmpR
pUC ori
tet operator
MBP
BGH poly(A)signal
FRT
Hygromycin B
SV40 poly(A)signal
BspTI (975)
HindIII (978)
Acc65I (984)
BamHI (2091)
TEV-L
BshTI (2118)
NheI (2152)
TEV-R
EcoRV (2181)
XhoI (2202)
Bsp120I (2214)
1000
2000
3000
4000
5000
6000

series pKK

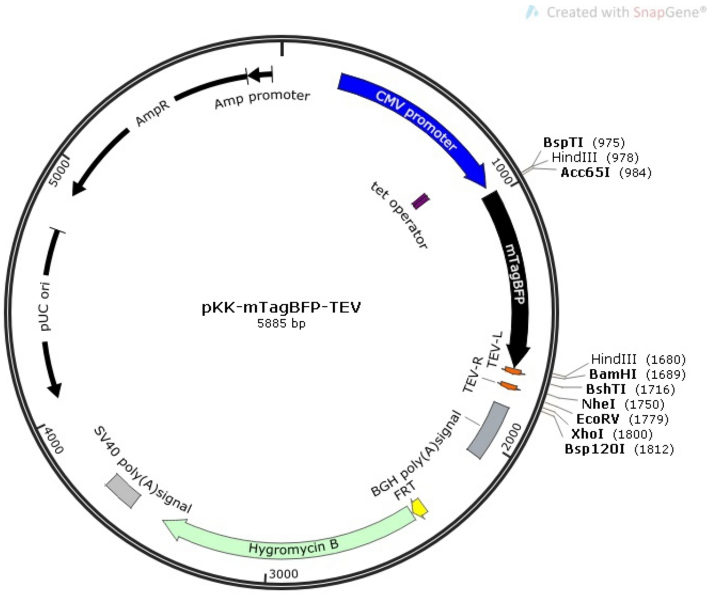
Created with SnapGene®
pKK-mTagBFP-TEV
5885 bp
Amp promoter
AmpR
CMV promoter
BspTI (975)
HindIII (978)
Acc65I (984)
tet operator
mTagBFP
TEV-L
TEV-R
HindIII (1680)
BamHI (1689)
BshTI (1716)
NheI (1750)
EcoRV (1779)
XhoI (1800)
Bsp120I (1812)
BGH poly(A)signal
FRT
Hygromycin B
SV40 poly(A)signal
pUC ori
1000
2000
3000
4000
5000

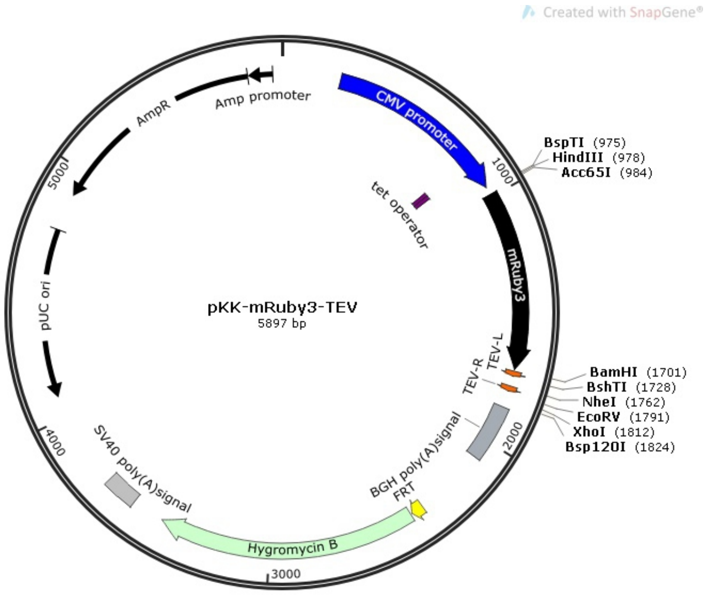
Created with SnapGene®
pKK-mRuby3-TEV
5897 bp
Amp promoter
AmpR
CMV promoter
BspTI (975)
HindIII (978)
Acc65I (984)
tet operator
mRuby3
TEV-L
TEV-R
BamHI (1701)
BshTI (1728)
NheI (1762)
EcoRV (1791)
XhoI (1812)
Bsp120I (1824)
BGH poly(A)signal
FRT
Hygromycin B
SV40 poly(A)signal
pUC ori
1000
2000
3000
4000
5000

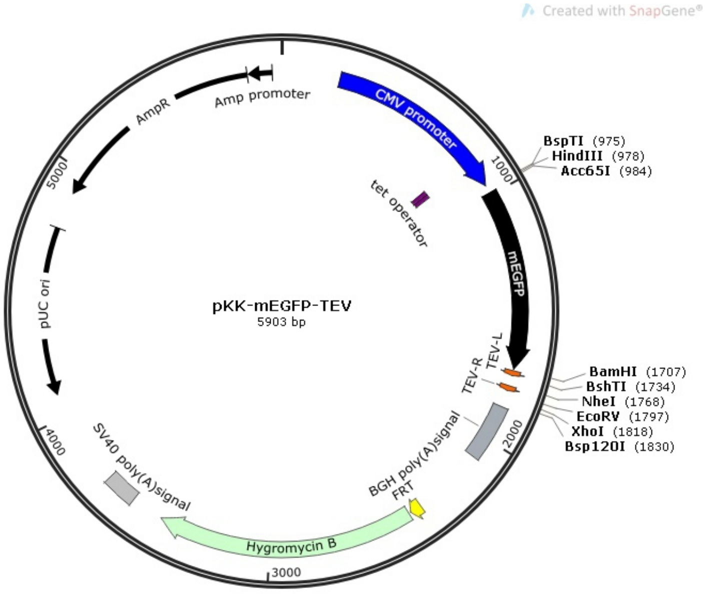
Created with SnapGene®
pKK-mEGFP-TEV
5903 bp
Amp promoter
AmpR
CMV promoter
BspTI (975)
HindIII (978)
Acc65I (984)
tet operator
mEGFP
TEV-L
TEV-R
BamHI (1707)
BshTI (1734)
NheI (1768)
EcoRV (1797)
XhoI (1818)
Bsp120I (1830)
BGH poly(A)signal
FRT
Hygromycin B
SV40 poly(A)signal
pUC ori
1000
2000
3000
4000
5000

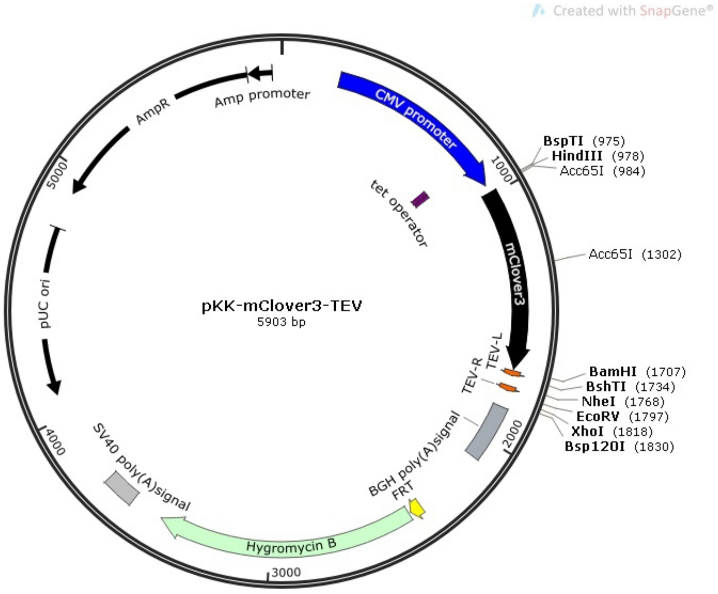
Created with SnapGene®
pKK-mClover3-TEV
5903 bp
Amp promoter
AmpR
CMV promoter
BspTI (975)
HindIII (978)
Acc65I (984)
tet operator
mClover3
Acc65I (1302)
TEV-L
TEV-R
BamHI (1707)
BshTI (1734)
NheI (1768)
EcoRV (1797)
XhoI (1818)
Bsp120I (1830)
BGH poly(A)signal
FRT
Hygromycin B
SV40 poly(A)signal
pUC ori
1000
2000
3000
4000
5000

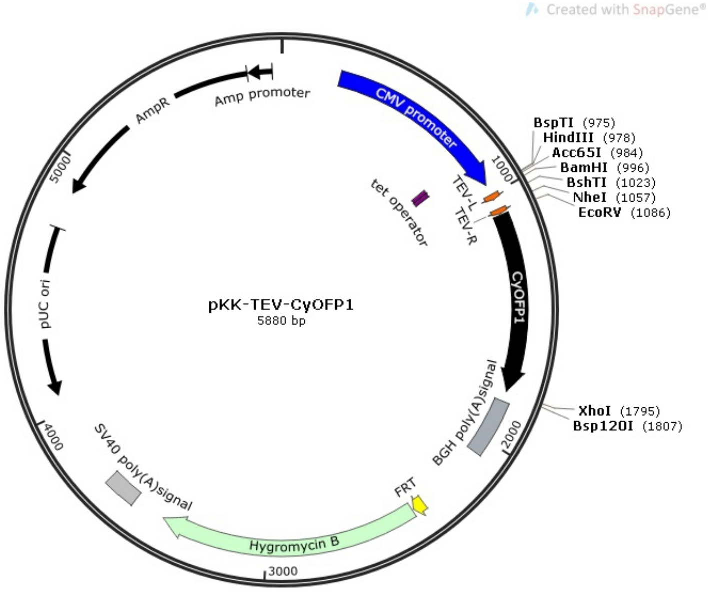
Created with SnapGene®
pKK-TEV-CyOFP1
5880 bp
Amp promoter
AmpR
CMV promoter
BspTI (975)
HindIII (978)
Acc65I (984)
BamHI (996)
BshTI (1023)
NheI (1057)
EcoRV (1086)
tet operator
TEV-L
TEV-R
CyOFP1
XhoI (1795)
Bsp120I (1807)
BGH poly(A)signal
FRT
Hygromycin B
SV40 poly(A)signal
pUC ori
1000
2000
3000
4000
5000

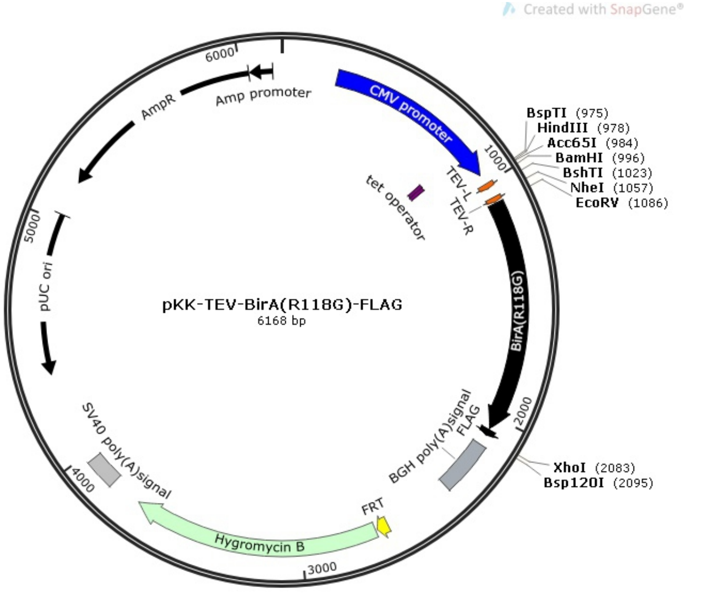
Created with SnapGene®
pKK-TEV-BirA(R118G)-FLAG
6168 bp
6000
Amp promoter
AmpR
CMV promoter
BspTI (975)
HindIII (978)
Acc65I (984)
BamHI (996)
BshTI (1023)
NheI (1057)
EcoRV (1086)
tet operator
TEV-L
TEV-R
BirA(R118G)
FLAG
XhoI (2083)
Bsp120I (2095)
BGH poly(A)signal
FRT
Hygromycin B
SV40 poly(A)signal
pUC ori
1000
2000
3000
4000
5000

series pKK

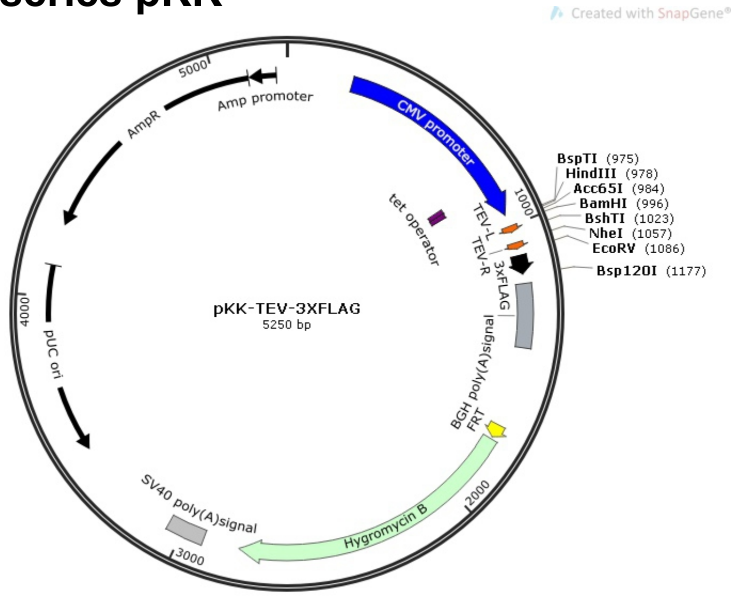

Created with SnapGene®
pKK-TEV-3XFLAG
5250 bp
CMV promoter
BspTI (975)
HindIII (978)
Acc65I (984)
BamHI (996)
BshTI (1023)
NheI (1057)
EcoRV (1086)
Bsp120I (1177)
tet operator
TEV-L
TEV-R
3xFLAG
BGH poly(A)signal
FRT
Hygromycin B
SV40 poly(A)signal
pUC ori
AmpR
Amp promoter
1000
2000
3000
4000
5000

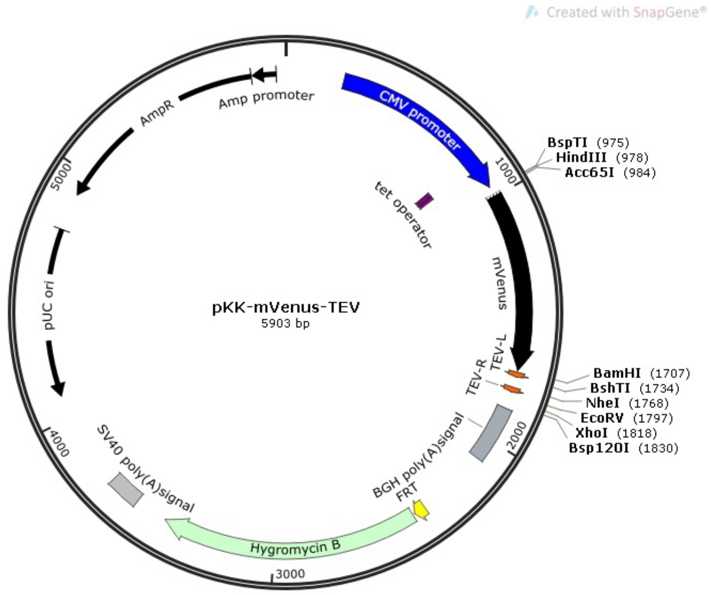

Created with SnapGene®
pKK-mVenus-TEV
5903 bp
CMV promoter
BspTI (975)
HindIII (978)
Acc65I (984)
tet operator
mVenus
TEV-L
TEV-R
BamHI (1707)
BshTI (1734)
NheI (1768)
EcoRV (1797)
XhoI (1818)
Bsp120I (1830)
BGH poly(A)signal
FRT
Hygromycin B
SV40 poly(A)signal
pUC ori
AmpR
Amp promoter
1000
2000
3000
4000
5000

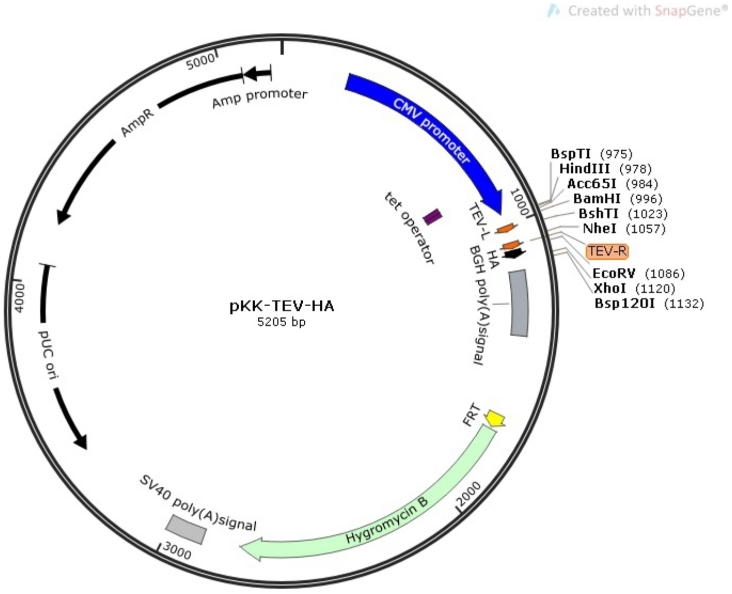

Created with SnapGene®
pKK-TEV-HA
5205 bp
CMV promoter
BspTI (975)
HindIII (978)
Acc65I (984)
BamHI (996)
BshTI (1023)
NheI (1057)
TEV-R
EcoRV (1086)
XhoI (1120)
Bsp120I (1132)
tet operator
TEV-L
HA
BGH poly(A)signal
FRT
Hygromycin B
SV40 poly(A)signal
pUC ori
AmpR
Amp promoter
1000
2000
3000
4000
5000

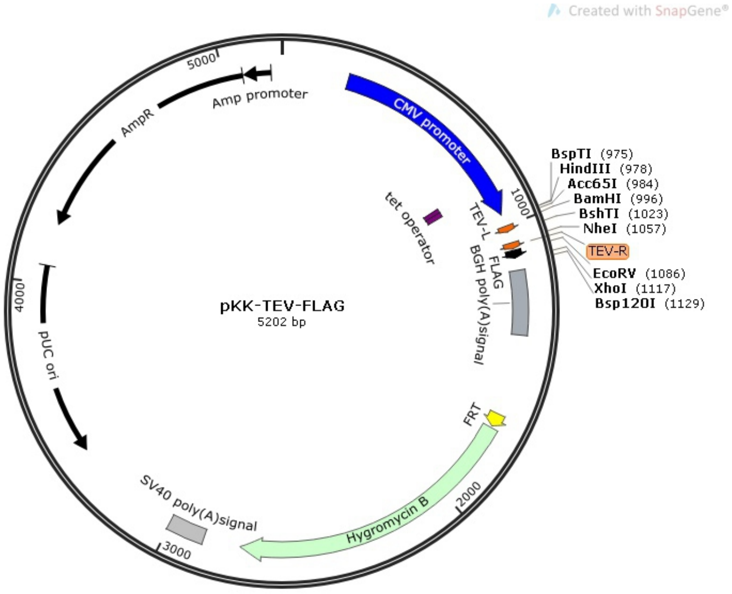

Created with SnapGene®
pKK-TEV-FLAG
5202 bp
CMV promoter
BspTI (975)
HindIII (978)
Acc65I (984)
BamHI (996)
BshTI (1023)
NheI (1057)
TEV-R
EcoRV (1086)
XhoI (1117)
Bsp120I (1129)
tet operator
TEV-L
FLAG
BGH poly(A)signal
FRT
Hygromycin B
SV40 poly(A)signal
pUC ori
AmpR
Amp promoter
1000
2000
3000
4000
5000

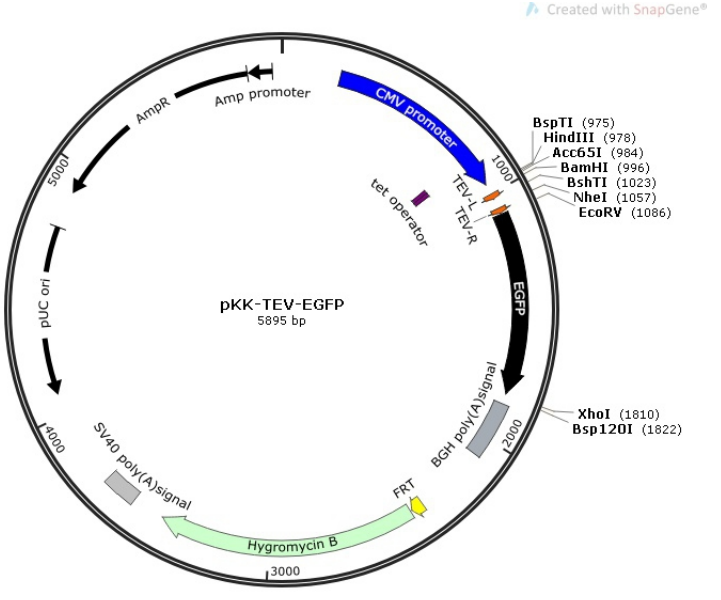

Created with SnapGene®
pKK-TEV-EGFP
5895 bp
CMV promoter
BspTI (975)
HindIII (978)
Acc65I (984)
BamHI (996)
BshTI (1023)
NheI (1057)
EcoRV (1086)
tet operator
TEV-L
TEV-R
EGFP
XhoI (1810)
Bsp120I (1822)
BGH poly(A)signal
FRT
Hygromycin B
SV40 poly(A)signal
pUC ori
AmpR
Amp promoter
1000
2000
3000
4000
5000

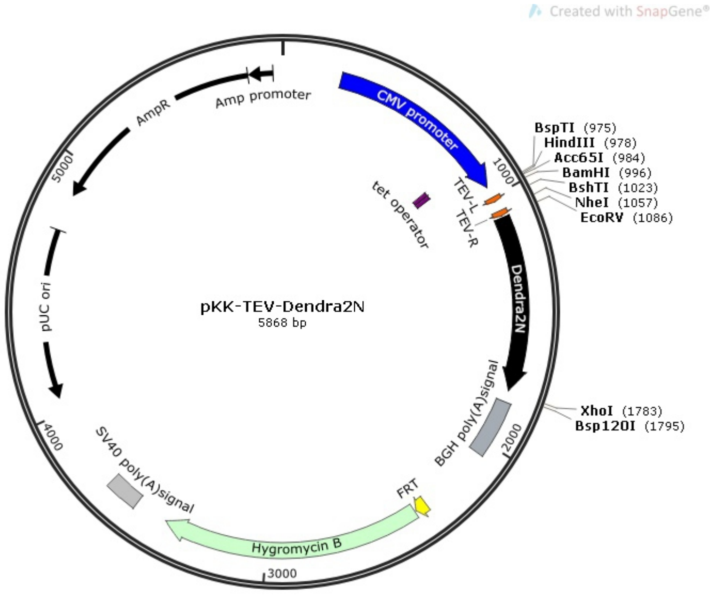

Created with SnapGene®
pKK-TEV-Dendra2N
5868 bp
CMV promoter
BspTI (975)
HindIII (978)
Acc65I (984)
BamHI (996)
BshTI (1023)
NheI (1057)
EcoRV (1086)
tet operator
TEV-L
TEV-R
Dendra2N
XhoI (1783)
Bsp120I (1795)
BGH poly(A)signal
FRT
Hygromycin B
SV40 poly(A)signal
pUC ori
AmpR
Amp promoter
1000
2000
3000
4000
5000

series pKK

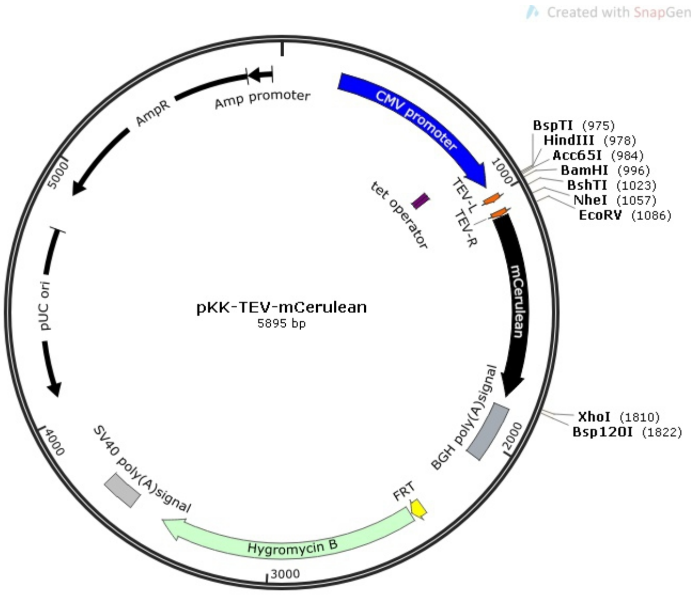
Created with SnapGene®
pKK-TEV-mCerulean
5895 bp
CMV promoter
Amp promoter
AmpR
pUC ori
SV40 poly(A)signal
Hygromycin B
FRT
BGH poly(A)signal
mCerulean
TEV-L
TEV-R
tet operator
BspTI (975)
HindIII (978)
Acc65I (984)
BamHI (996)
BshTI (1023)
NheI (1057)
EcoRV (1086)
XhoI (1810)
Bsp120I (1822)
1000
2000
3000
4000
5000

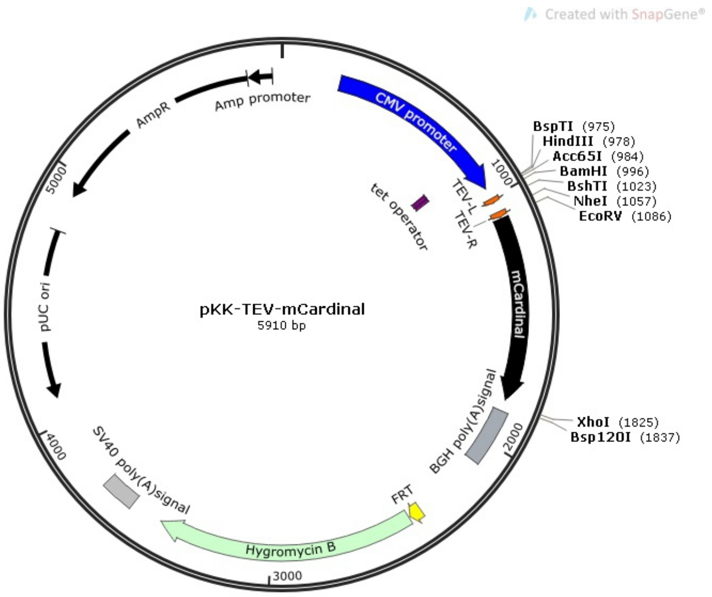
Created with SnapGene®
pKK-TEV-mCardinal
5910 bp
CMV promoter
Amp promoter
AmpR
pUC ori
SV40 poly(A)signal
Hygromycin B
FRT
BGH poly(A)signal
mCardinal
TEV-L
TEV-R
tet operator
BspTI (975)
HindIII (978)
Acc65I (984)
BamHI (996)
BshTI (1023)
NheI (1057)
EcoRV (1086)
XhoI (1825)
Bsp120I (1837)
1000
2000
3000
4000
5000

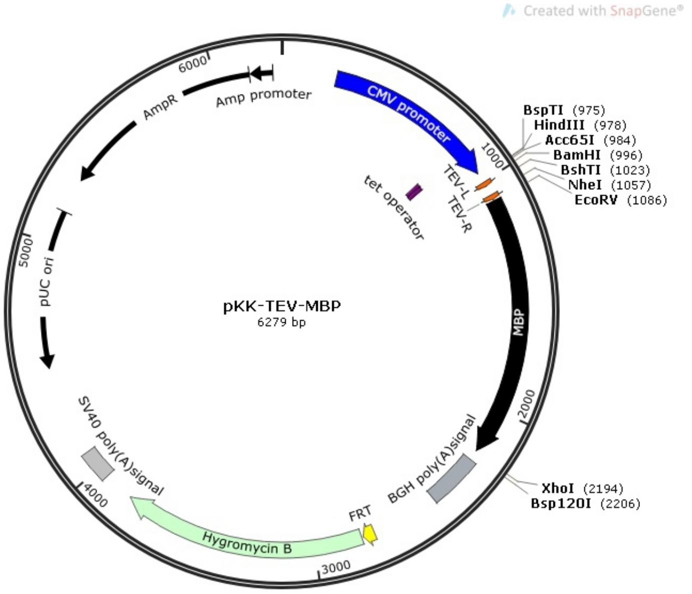
Created with SnapGene®
pKK-TEV-MBP
6279 bp
CMV promoter
Amp promoter
AmpR
pUC ori
SV40 poly(A)signal
Hygromycin B
FRT
BGH poly(A)signal
MBP
TEV-L
TEV-R
tet operator
BspTI (975)
HindIII (978)
Acc65I (984)
BamHI (996)
BshTI (1023)
NheI (1057)
EcoRV (1086)
XhoI (2194)
Bsp120I (2206)
1000
2000
3000
4000
5000
6000

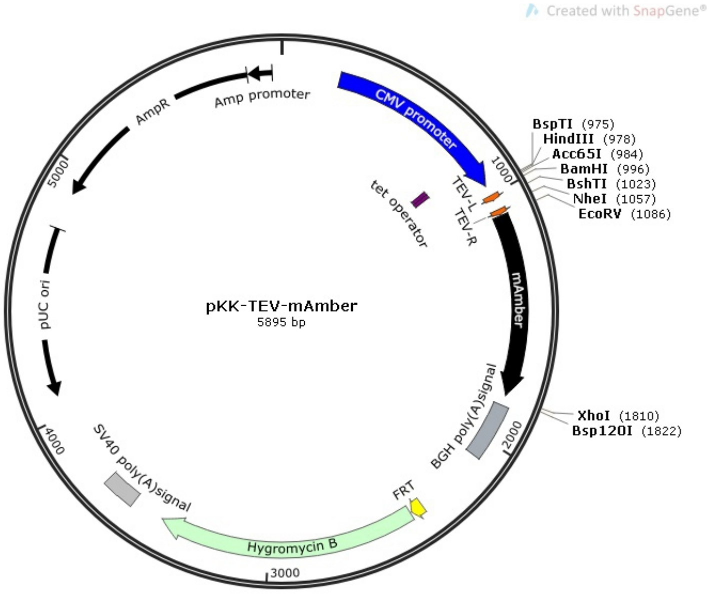
Created with SnapGene®
pKK-TEV-mAmber
5895 bp
CMV promoter
Amp promoter
AmpR
pUC ori
SV40 poly(A)signal
Hygromycin B
FRT
BGH poly(A)signal
mAmber
TEV-L
TEV-R
tet operator
BspTI (975)
HindIII (978)
Acc65I (984)
BamHI (996)
BshTI (1023)
NheI (1057)
EcoRV (1086)
XhoI (1810)
Bsp120I (1822)
1000
2000
3000
4000
5000

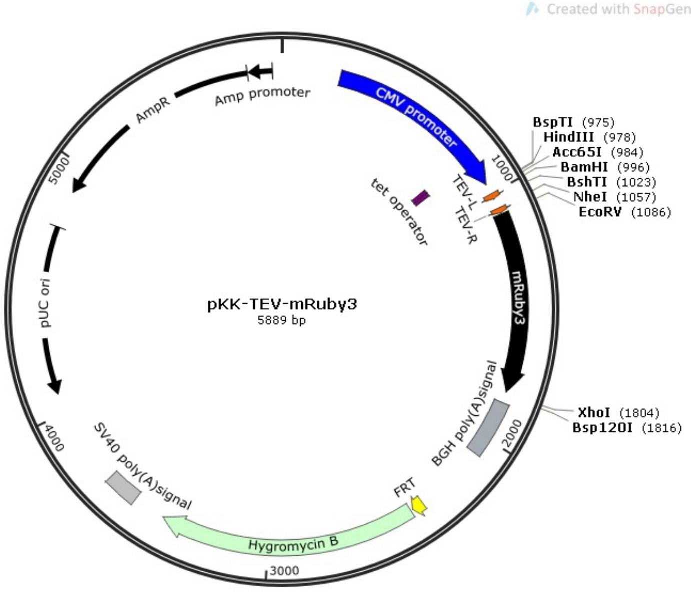
Created with SnapGene®
pKK-TEV-mRuby3
5889 bp
CMV promoter
Amp promoter
AmpR
pUC ori
SV40 poly(A)signal
Hygromycin B
FRT
BGH poly(A)signal
mRuby3
TEV-L
TEV-R
tet operator
BspTI (975)
HindIII (978)
Acc65I (984)
BamHI (996)
BshTI (1023)
NheI (1057)
EcoRV (1086)
XhoI (1804)
Bsp120I (1816)
1000
2000
3000
4000
5000

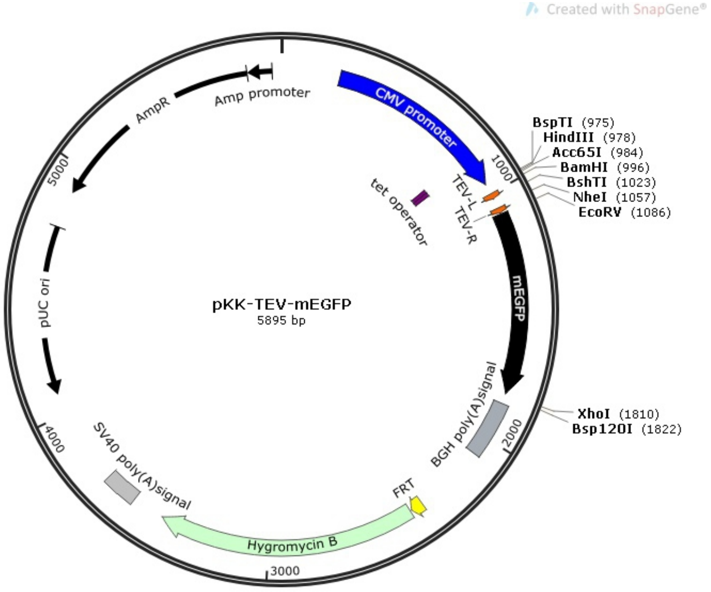
Created with SnapGene®
pKK-TEV-mEGFP
5895 bp
CMV promoter
Amp promoter
AmpR
pUC ori
SV40 poly(A)signal
Hygromycin B
FRT
BGH poly(A)signal
mEGFP
TEV-L
TEV-R
tet operator
BspTI (975)
HindIII (978)
Acc65I (984)
BamHI (996)
BshTI (1023)
NheI (1057)
EcoRV (1086)
XhoI (1810)
Bsp120I (1822)
1000
2000
3000
4000
5000

series pKK

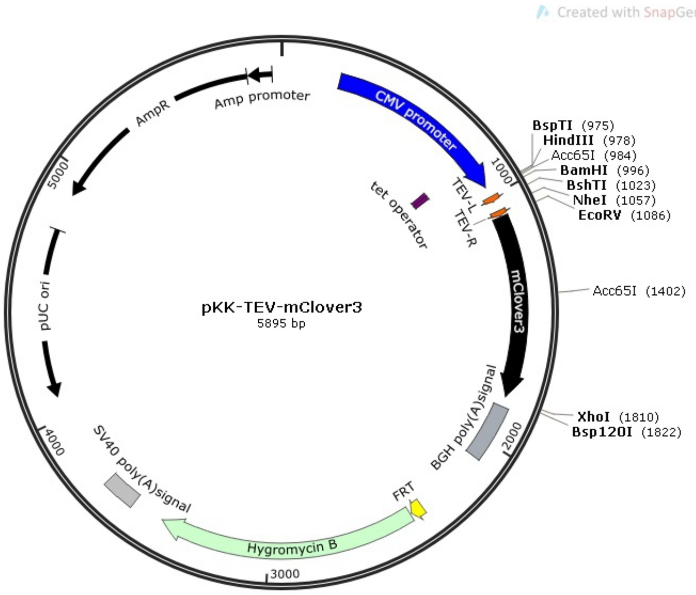

Created with SnapGene®
pKK-TEV-mClover3
5895 bp
CMV promoter
BspTI (975)
HindIII (978)
Acc65I (984)
BamHI (996)
BshTI (1023)
NheI (1057)
EcoRV (1086)
TEV-L
TEV-R
tet operator
mClover3
Acc65I (1402)
XhoI (1810)
Bsp120I (1822)
BGH poly(A)signal
FRT
Hygromycin B
SV40 poly(A)signal
pUC ori
AmpR
Amp promoter
1000
2000
3000
4000
5000

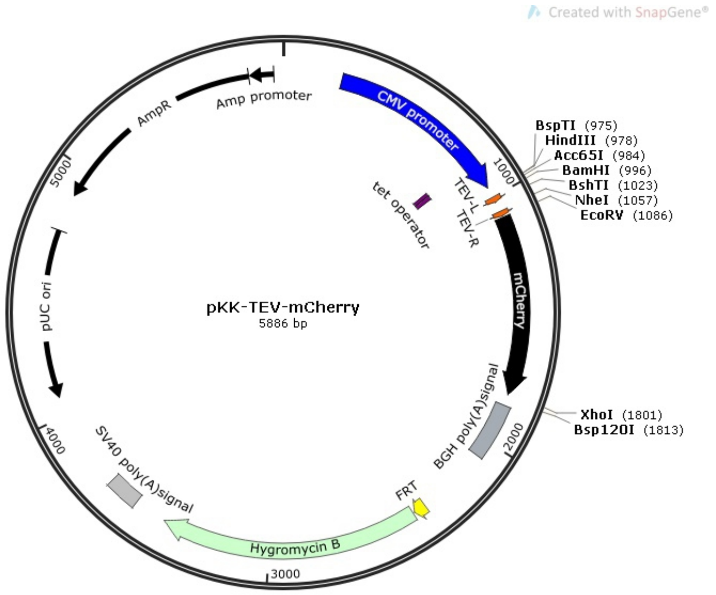

Created with SnapGene®
pKK-TEV-mCherry
5886 bp
CMV promoter
BspTI (975)
HindIII (978)
Acc65I (984)
BamHI (996)
BshTI (1023)
NheI (1057)
EcoRV (1086)
TEV-L
TEV-R
tet operator
mCherry
XhoI (1801)
Bsp120I (1813)
BGH poly(A)signal
FRT
Hygromycin B
SV40 poly(A)signal
pUC ori
AmpR
Amp promoter
1000
2000
3000
4000
5000

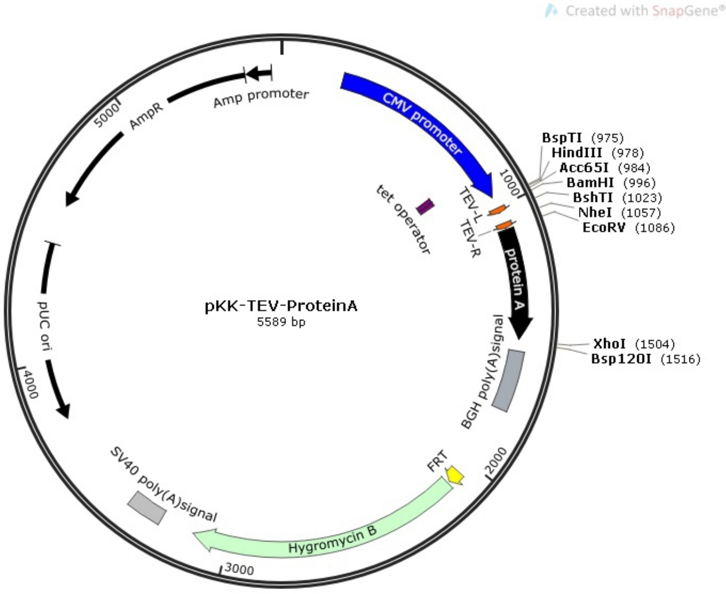

Created with SnapGene®
pKK-TEV-ProteinA
5589 bp
CMV promoter
BspTI (975)
HindIII (978)
Acc65I (984)
BamHI (996)
BshTI (1023)
NheI (1057)
EcoRV (1086)
TEV-L
TEV-R
tet operator
protein A
XhoI (1504)
Bsp120I (1516)
BGH poly(A)signal
FRT
Hygromycin B
SV40 poly(A)signal
pUC ori
AmpR
Amp promoter
1000
2000
3000
4000
5000

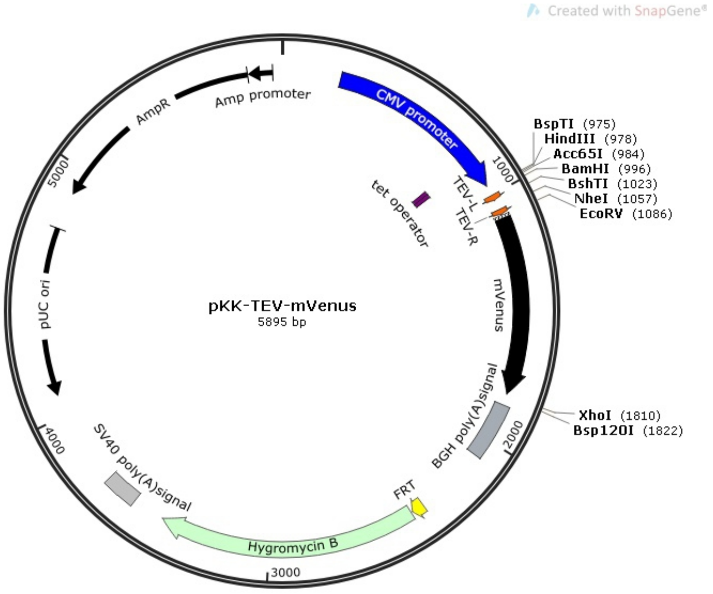

Created with SnapGene®
pKK-TEV-mVenus
5895 bp
CMV promoter
BspTI (975)
HindIII (978)
Acc65I (984)
BamHI (996)
BshTI (1023)
NheI (1057)
EcoRV (1086)
TEV-L
TEV-R
tet operator
mVenus
XhoI (1810)
Bsp120I (1822)
BGH poly(A)signal
FRT
Hygromycin B
SV40 poly(A)signal
pUC ori
AmpR
Amp promoter
1000
2000
3000
4000
5000

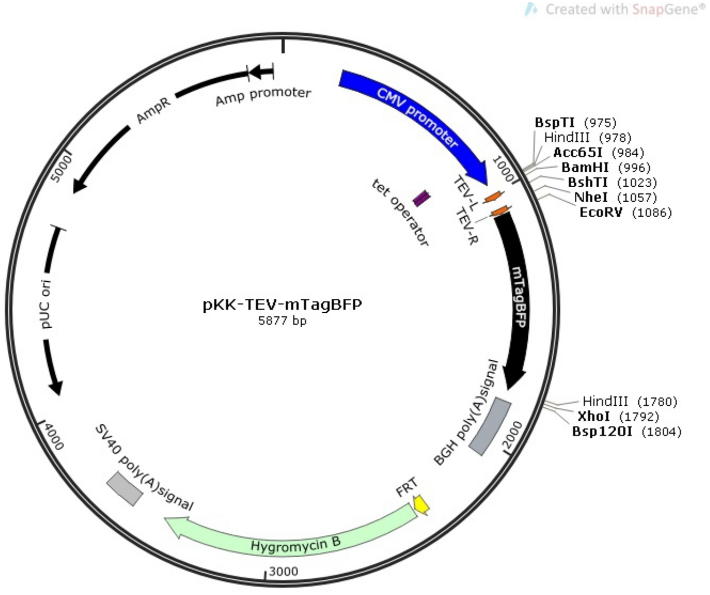

Created with SnapGene®
pKK-TEV-mTagBFP
5877 bp
CMV promoter
BspTI (975)
HindIII (978)
Acc65I (984)
BamHI (996)
BshTI (1023)
NheI (1057)
EcoRV (1086)
TEV-L
TEV-R
tet operator
mTagBFP
HindIII (1780)
XhoI (1792)
Bsp120I (1804)
BGH poly(A)signal
FRT
Hygromycin B
SV40 poly(A)signal
pUC ori
AmpR
Amp promoter
1000
2000
3000
4000
5000

series pKK-BI16

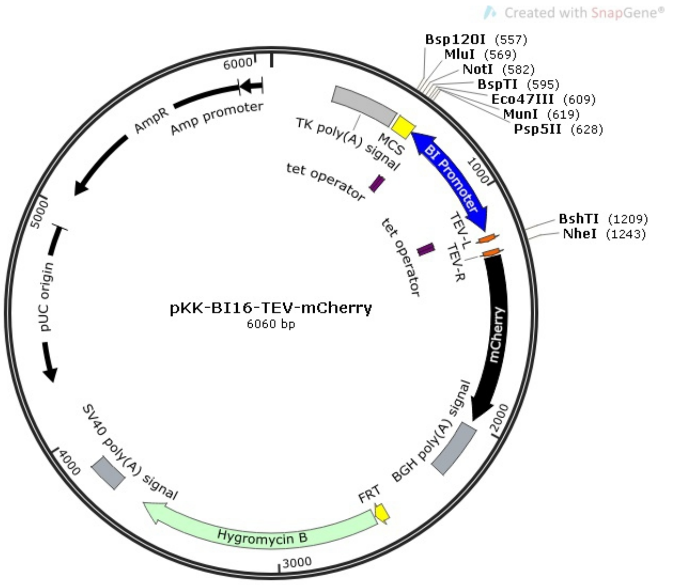

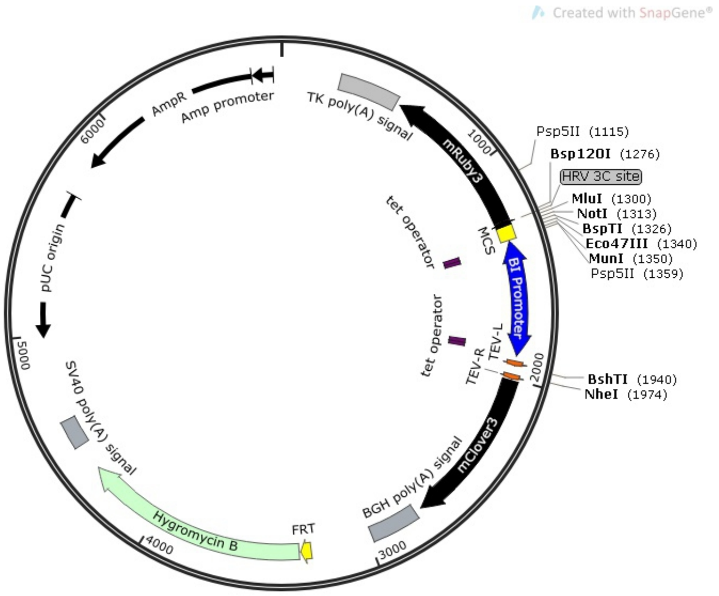

pKK-BI16-ORF1-C3-mRuby3_ORF2-TEV-mClover3
6800 bp

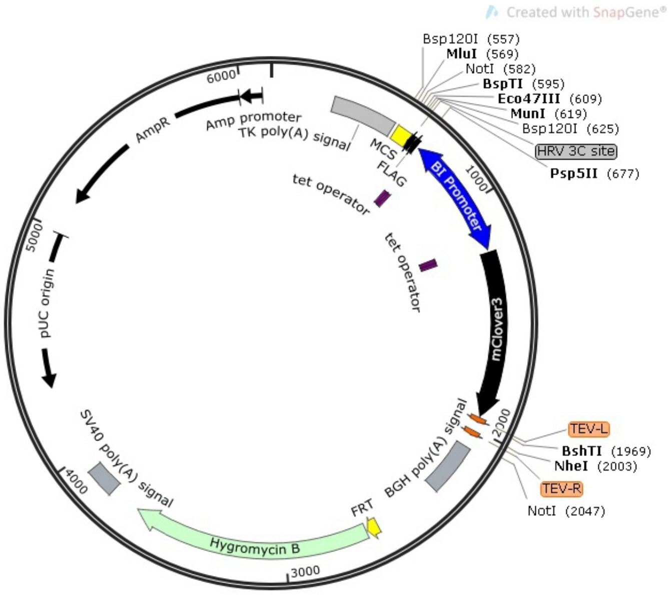

pKK-BI16-FLAG-C3-ORF1_mClover3-TEV-ORF2
6126 bp

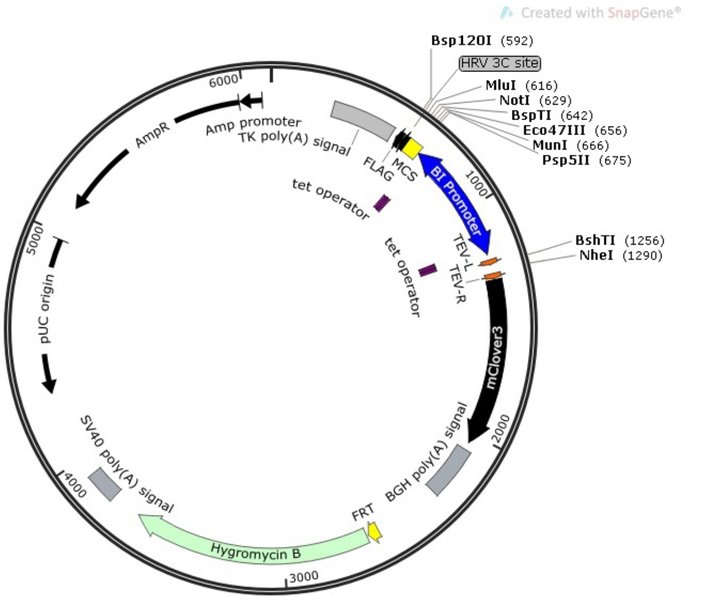

pKK-BI16-ORF1-C3-FLAG_ORF2-TEV-mClover3
6116 bp

series pKK-BiFC

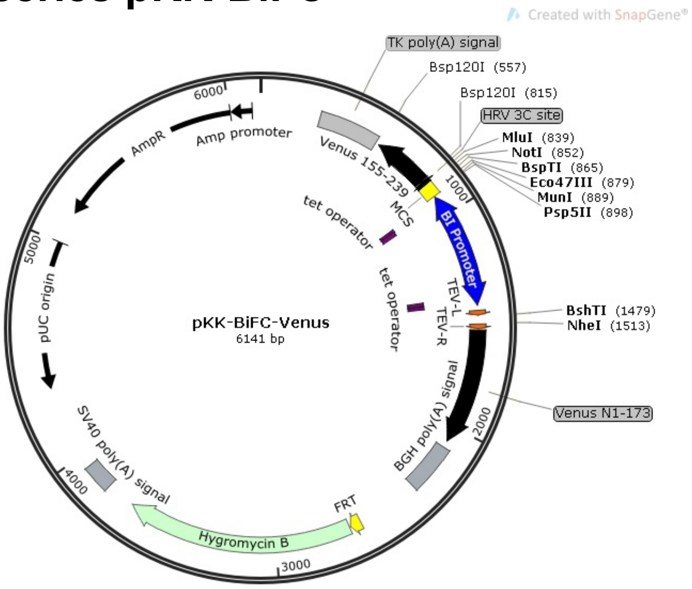

series pKK-FRET

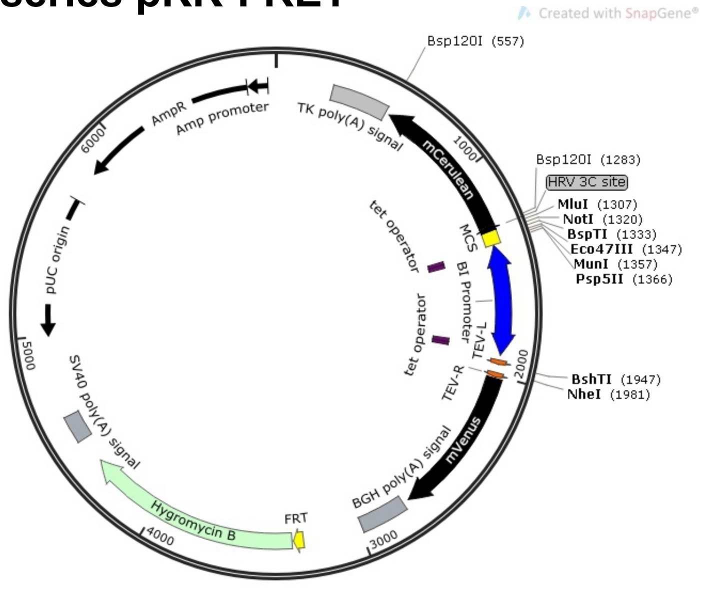

pKK-FRET-Orf1-C3-mCerulean_Orf2-TEV-mVenus
6807 bp

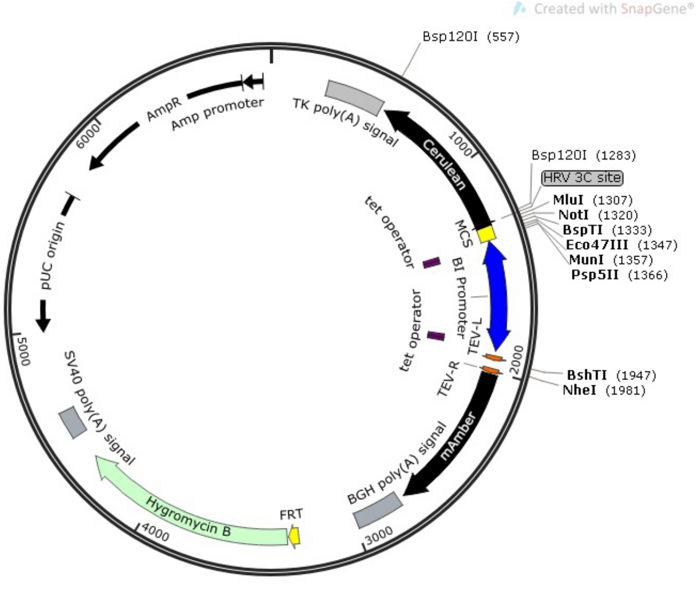

pKK-FRET-ORF1-C3-mCerulean_ORF2-TEV-mAmber
6807 bp

series pKK-RNAtag

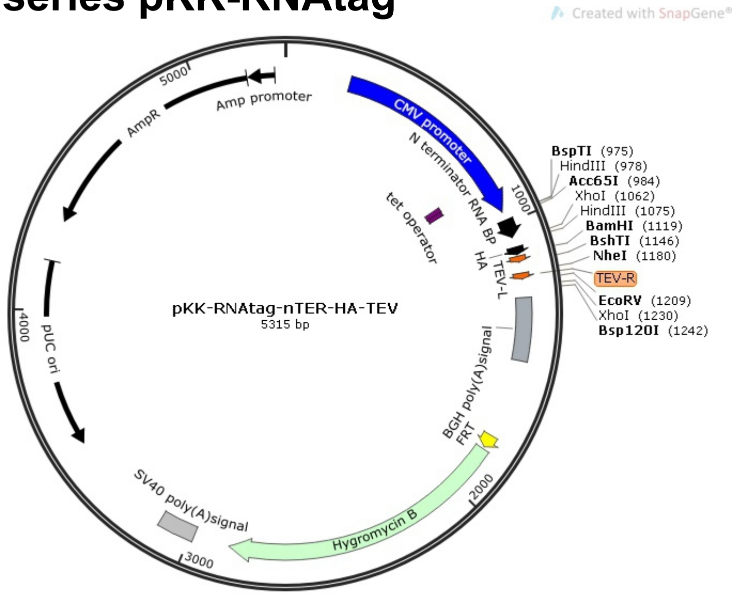

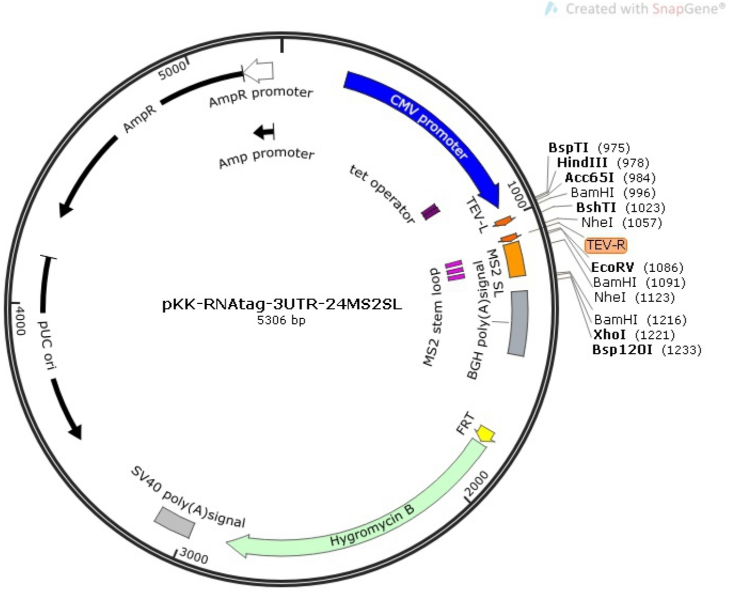

series pKK-RNAi

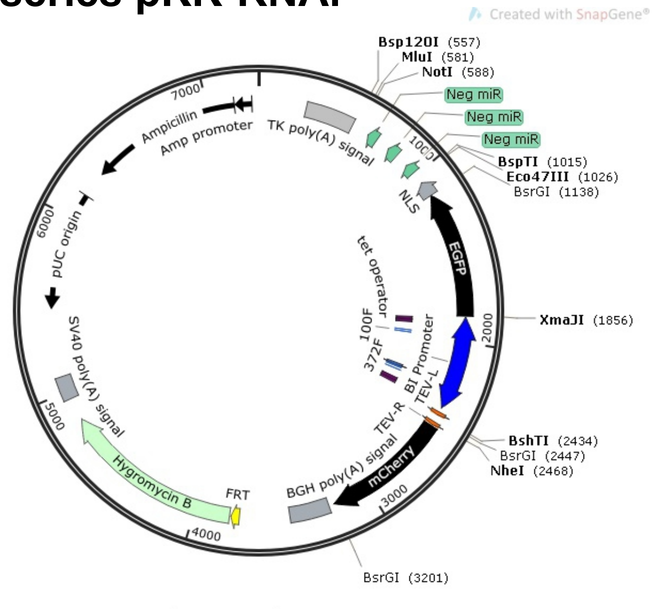

pKK-RNAi-nucEGFPmiR-TEV-mCHERRY
7285 bp

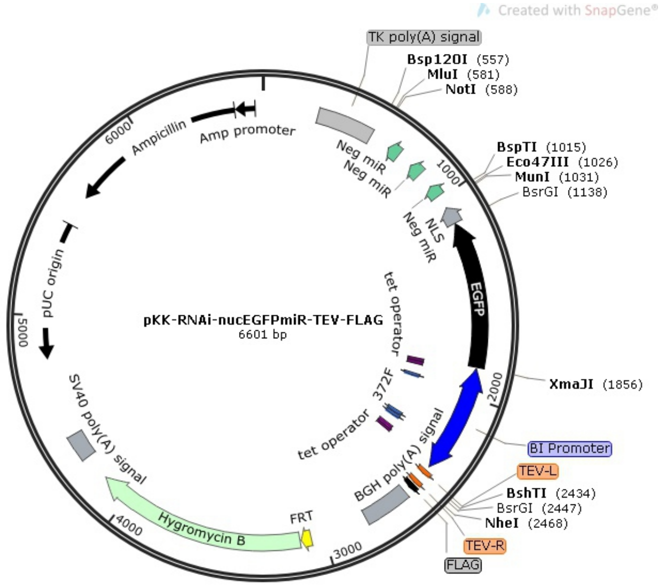

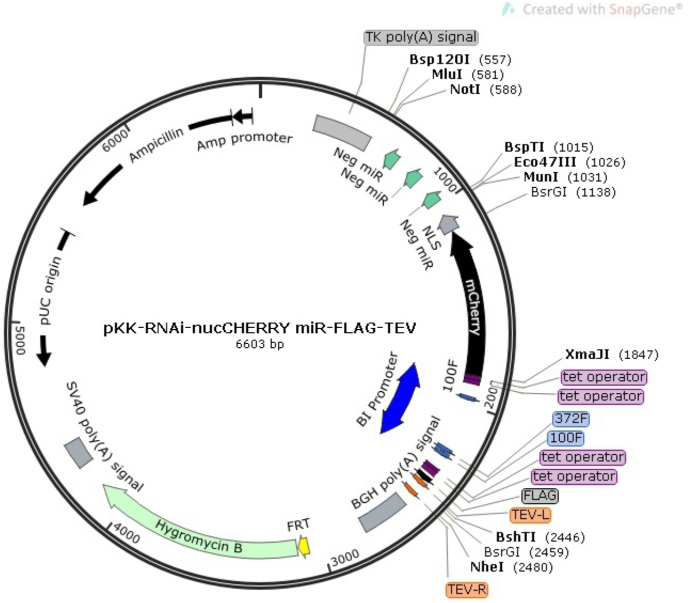

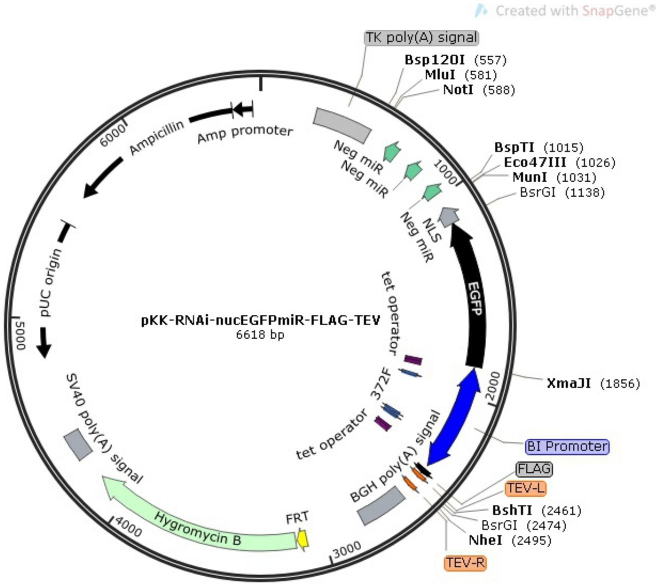

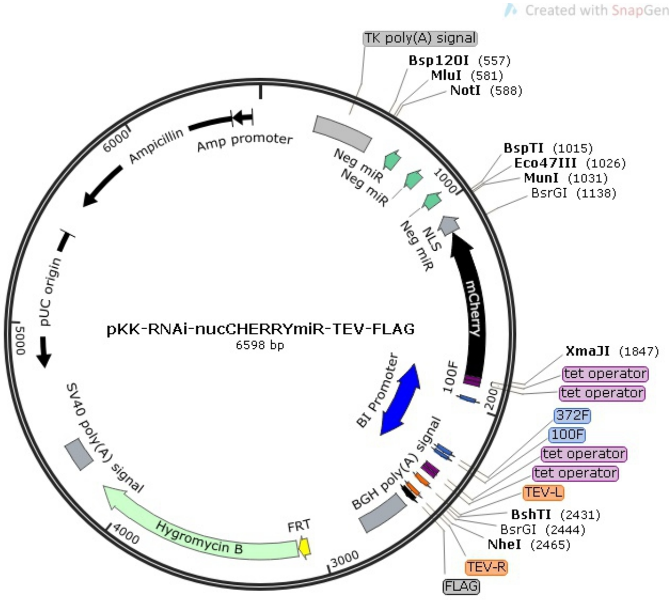

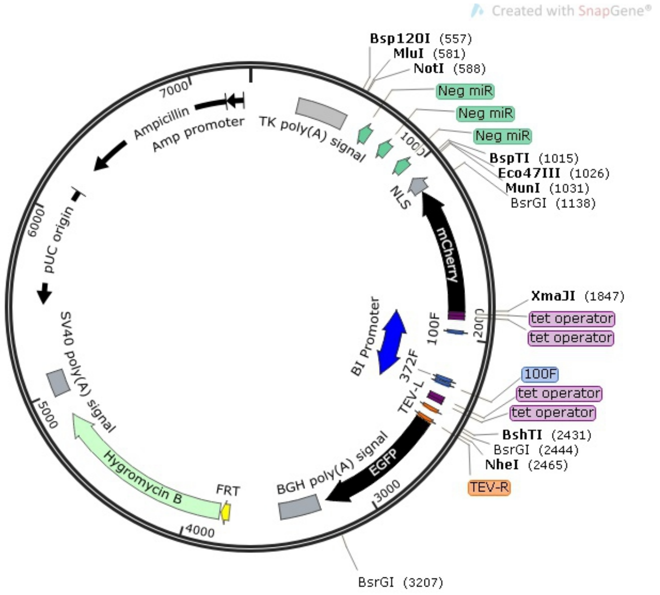

pKK-RNAi-nucCHERRYmiR-TEV-EGFP
7304 bp

series pKK-RNAi

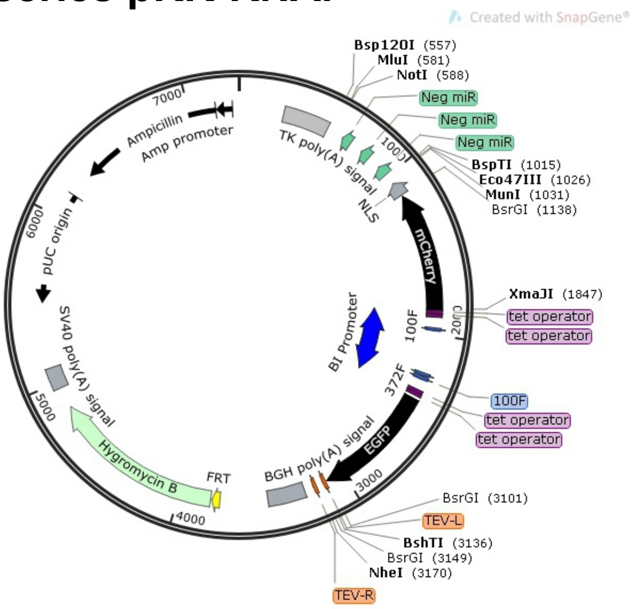

pKK-RNAi-nucCHERRYmiR-EGFP-TEV
7293 bp

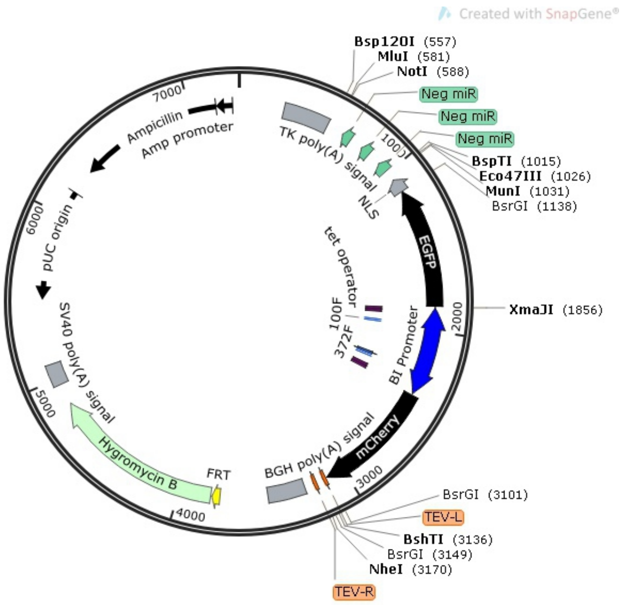

pKK-RNAi-nucEGFPmiR-mCherry-TEV
7293 bp
